# In Vivo Evaluation of Pam_2_Cys‐Modified Cancer‐Testis Antigens as Potential Self‐Adjuvanting Cancer Vaccines

**DOI:** 10.1002/psc.70022

**Published:** 2025-05-06

**Authors:** Salwa Aljohani, Alex G. Edmonds, Valeria Castelletto, Jani Seitsonen, Ian W. Hamley, Peter Symonds, Victoria A. Brentville, Lindy G. Durrant, Nicholas J. Mitchell

**Affiliations:** ^1^ School of Chemistry, University of Nottingham University Park Nottingham UK; ^2^ School of Chemistry, Pharmacy and Food Biosciences University of Reading Reading UK; ^3^ Nanomicroscopy Center Aalto University Espoo Finland; ^4^ Scancell, Biodiscovery Institute, University of Nottingham University Park Nottingham UK

**Keywords:** adjuvant, cancer‐testis antigen, peptide, vaccine

## Abstract

Peptide‐based vaccines, formulated with an appropriate adjuvant, offer a versatile platform for targeted cancer immunotherapy. While adjuvants are usually coadministered for nucleic acid and protein vaccines, synthetic peptide antigens afford a more effective opportunity to covalently and regioselectively graft immunostimulatory motifs directly onto the antigen scaffold to yield *self‐adjuvanting* vaccines. Herein, we explore the synthesis of two tissue‐restricted cancer‐testis antigens (CTAs); New York oesophageal cell carcinoma 1 (NY‐ESO‐1) and B melanoma antigen 4 (BAGE4), both carrying the toll‐like receptor (TLR) agonist, Pam_2_Cys. These constructs were evaluated in vivo along with a lipid nanoparticle (LNP) preparation of the underexplored BAGE4 melanoma antigen.

## Graphical abstract

Peptide‐based vaccines, formulated with an appropriate adjuvant, offer a versatile platform for targeted cancer immunotherapy. Herein, we explore the synthesis of two tissue‐restricted cancer‐testis antigens; (NY‐ESO‐1 and BAGE4) covalently linked to the toll‐like receptor agonist, Pam_2_Cys. These constructs were evaluated in vivo along with a lipid nanoparticle formulation of BAGE4.

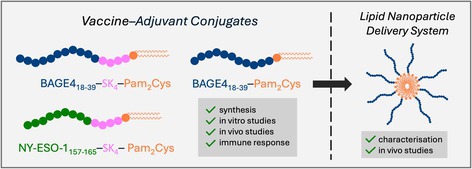

## 1 Introduction

Immunotherapy has emerged in recent years as an alternative cancer treatment that can be administered without the drawbacks of more conventional approaches such as chemotherapy, radiotherapy and surgery [1]. The production of tumour‐associated antigens (TAAs) provides an opportunity to direct the patient's immune system to target cancer via vaccination [1]. Cancer‐testis antigens (CTAs), a class of highly tissue‐restricted TAA, expressed in male germ‐line cells and aberrantly expressed across a broad range of cancer types, have been explored as promising targets for vaccine development [2, 3, 4]. Due to the existence of the blood‐testis barrier, and the lack of human leukocyte antigen (HLA) class I expression on the surface of germ cells, CTAs do not activate the autoimmune response; a process that may hinder the viability of cancer immunotherapy treatments [5]. The most promising CTA candidate to‐date is New York oesophageal cell carcinoma 1 (NY‐ESO‐1) [6]; this TAA demonstrates strong spontaneous humoral and cell‐mediated immune responses [2]. Clinical trials have been conducted for this antigen employing a range of strategies, including peptide (epitope), protein, nucleic acid (DNA and mRNA), dendritic cell (DC) and whole‐tumour cell therapy via viral, bacterial and lipid nanoparticle (LNP) delivery vectors [7, 8, 9, 10]. While these trials have produced promising initial results, many have not progressed to Phase III. Unfortunately, the results of those trials that were progressed to completion have been ultimately disappointing [2, 11].

Despite the robust immune response TAAs such as NY‐ESO‐1 can elicit, few antigens produce a strong enough response to challenge cancer without coadministration of an adjuvant—a substance that recruits antigen‐presenting cells (APCs), increasing the delivery of antigens to APCs or activating APCs to produce cytokines and trigger T‐cell responses [12]. In both academic and clinical settings, CTAs have been coadministered with several effective adjuvants such as incomplete Freund's adjuvant (IFA), ISCOMATRIX, cytokines and toll‐like receptor (TLR) agonists such as monophosphoryl lipid A and CpG oligonucleotides (ODN) [12]. TLR agonists in particular have been shown to be effective adjuvants for a range of vaccines [13]. Ten TLR variants are present in humans; the TLR family display affinities for particular pathogen‐associated molecular patterns (PAMPs), that is, bacterial and viral components [14, 15]. TLR2 (activated with TLR6) recognises lipopeptides derived from the bacterial cell wall. Pam_2_Cys, a synthetic analogue of the lipid component of macrophage‐activating lipopeptide‐2 (MALP‐2), is a potent TLR2/6 agonist [14, 15]. Pam_2_Cys (as well as Pam_3_Cys) are among the most common lipid moieties used in the production of lipopeptide vaccines [16], several of which have advanced to human clinical trials, showing a high level of protection with little to no side effects reported [17, 18, 19]. This adjuvant can be effectively coadministered as PEG‐Pam_2_Cys [20] or as the palmitoylated cysteine residue along with other lipids (and cholesterol) formulated into a LNP as a delivery vehicle for DNA/mRNA vaccines [21].

While coadministration of the adjuvant has proven to be effective, the ability to covalently graft the adjuvant moiety onto the antigen scaffold enables the preparation of a *self‐adjuvanting* vaccine. Such multicomponent constructs may circumvent undesired immune responses and have been demonstrated to be promising immunotherapy tools [22]. In this regard, peptide‐based vaccines [23] (sequences that represent highly immunogenic epitopes of protein antigens) are ideal antigen scaffolds. Noncanonical amino acids bearing adjuvant groups can be incorporated into the peptide sequence during solid‐phase synthesis. Alternatively, chemo‐ and regio‐selective chemical modification of the synthesised peptide using established bioconjugate chemistry can be applied. Using the former strategy, multiple‐component Pam_2_Cys‐peptide vaccines have been prepared and shown to produce robust immune responses against (among others) cancer [24], *Mycobacterium tuberculosis* [25] and SARS‐CoV‐2 [26].

Due to the small size of many CTAs (several under 100 amino acids in length), it is possible to produce the whole CTA synthetically via solid‐phase peptide synthesis (SPPS), coupled with native chemical ligation if required [27]. Recently, Brimble and co‐workers employed an NY‐ESO‐1 epitope bearing Pam_2_Cys and relevant analogues to illustrate the importance of the Pam_2_Cys stereochemistry (following on from earlier studies demonstrating that the *R* stereoisomer in the glyceryl moiety is significantly more active than the *S*) [28] and ester linkages, using an in vitro reporter system [17, 18, 19]. These structure–activity relationship studies demonstrated that the C16 chain is optimal for activity; although homologues of Pam_2_Cys are still active, short‐chain fatty acid alternatives induce a very weak response [19]. Replacement of the fatty acid chains with unsaturated groups, polyether or polyamine functionalities also decreases potency [19]. Additionally, the ester linkage has been shown to be essential for activity [17]. While the NY‐ESO‐1 epitope carrying Pam_2_Cys was shown to be the most efficacious of the analogues, this product was not evaluated in vivo during these studies.

**FIGURE 1:**
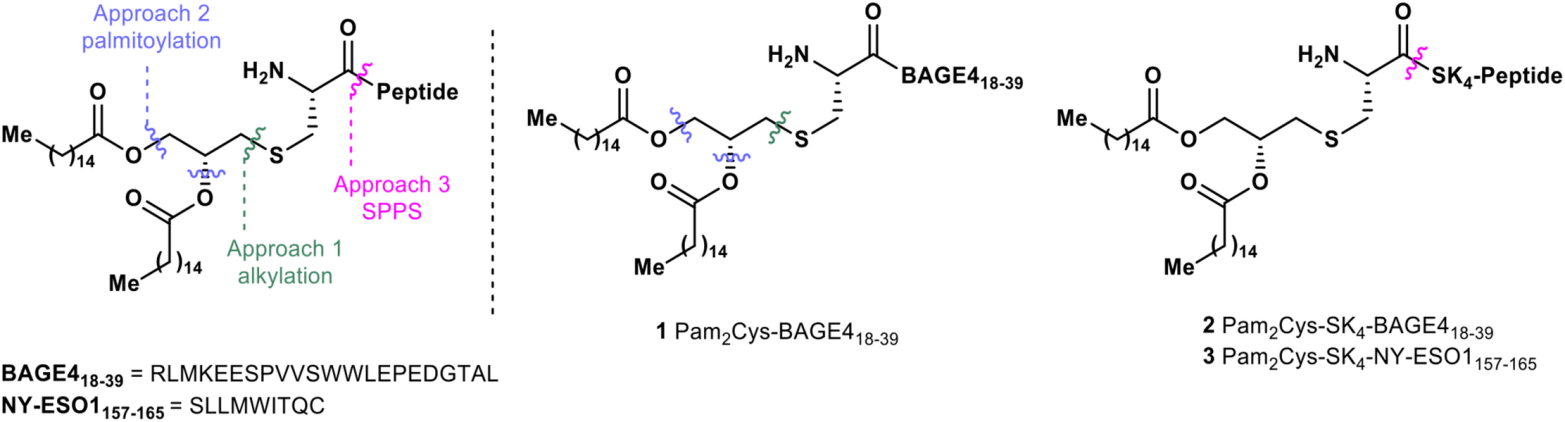
Approaches to the construction of vaccine‐adjuvant conjugates **1**–**3**.

Due to the initial promise of NY‐ESO‐1 as a vaccine candidate, the lack of in vivo evaluation of the palmitoylated analogue of this antigen, and the lack of in vivo data for many other CTAs, including the B melanoma antigen 4 (BAGE4) sequence [29], further evaluation of the self‐adjuvanting efficacy of these scaffolds warrants additional exploration. Herein, we describe the synthesis, formulation and in vivo evaluation in healthy models of two CTA epitope peptides covalently attached to the lipid adjuvant, Pam_2_Cys; BAGE4_18–39_ [29] a small (22 amino acid, excluding the signal peptide) underexplored melanoma antigen, which has yet to be evaluated in vivo [29], and the NY‐ESO‐1 epitope, NY‐ESO‐1_157‐165_ (SLLMWITQC) [30]. Three approaches were explored for the preparation of the Pam_2_Cys modified antigens **1**–**3** (Figure 1); (1) alkylation of antigen peptide bearing an N‐terminal cysteine (Cys) residue with a bespoke iodinated ester moiety; (2) palmitoylation of glycerol installed onto the Cys sidechain, on‐resin; (3) synthesis and coupling of the Fmoc‐Pam_2_Cys‐OH (**4**) building block into the peptide sequence, on‐resin.

Vaccine constructs with and without a peptide solubility tag (SK_4_) [31] were successfully synthesised, and the constructs Pam_2_Cys‐BAGE4_18–39_ **1**, Pam_2_Cys‐SK_4_‐BAGE4_18–39_ **2** and Pam_2_Cys‐SK_4_‐NY‐ESO‐1_157‐165_ **3** were evaluated in vitro. In vivo evaluation of the immune response elicited by candidates **2** and **3** was then conducted by measuring the T‐cell responses to these epitopes in healthy mice. Furthermore, LNPs formulated from BAGE4 candidate **2** were compared to LNPs carrying the BAGE4 peptide conjugated to the outer envelope of the LNP via in vivo evaluation.

At the outset of our investigation, Pam_2_Cys‐CTAs had not been explored as vaccine candidates. However, during the course of our studies, Brimble et al. described the synthesis of Pam_2_Cys‐NY‐ESO peptide via approach 1 and conducted in vitro testing of the human and mouse TLR2 agonistic activities of lipopeptide homologues of Pam_2_Cys using HEK293 cells [17]. Approaches 2 and 3 to Pam_2_Cys‐functionalised peptides have been previously described by Jackson et al. and Brimble et al., respectively [19, 32].

## 2 Materials and Methods

### 2.1 General Methods, Reagents and Chemical Synthesis

General methods for all chemical syntheses are included in the Supporting Information. All chemicals were of commercial quality and were used without additional purification. All commercially available reagents and reagent‐grade solvents were purchased from Merck, Fluorochem or Fisher and used as received unless otherwise stated. Amino acids, coupling reagents and resins were obtained from Novabiochem, Fluorochem or GL Biochem. Antibodies were purchased from Sigma Aldrich; DSPC and DOTAP were purchased from Avanti Polar Lipids Inc. Experimental procedures for the synthesis, purification and characterization of the novel maleimide lipid (MalLipid **5**) are described in the Supporting Information and in earlier reported work [33]. All aqueous solutions were prepared using deionised water. Dry solvents were used when indicated in the procedure.

### 2.2 Formulation of LNP With Vaccine Construct 2 (2‐LNP)

The constituent lipids (DSPC and DOTAP) and cholesterol were dissolved in chloroform to a conc. of 2 mM; Pam_2_Cys‐SK_4_‐BAGE4_18–39_ **2** was dissolved in methanol to a conc. of 2 mM. The lipid and cholesterol components were mixed in a DSPC:DOTAP:cholesterol:**2** ratio of 40:15:35:10. Solvents were evaporated in vacuo to form a continuous lipid film, which was dried in vacuo overnight. Milli‐Q water (0.9 mL) was added to hydrate the film with vortexing at 55°C. The resulting mixture was sonicated for 10 min, no pulsing, amplitude at 90% (SONICS Vibra‐Cell, CPX 130) in an ice bath and 0.1 mL of 10× PBS buffer added to the formulation to give a final conc. of 2 mM.

### 2.3 Formulation of Antigen‐Conjugated LNP (CysBAGE4‐MalLNP)

The constituent lipids (DSPC, DOTAP, MalLipid **5**) and cholesterol were dissolved in chloroform to a conc. of 2 mM. The lipid and cholesterol components were mixed in a DSPC:DOTAP:MalLipid **5**:cholesterol ratio of 40:15:10:35. The solvent was evaporated in vacuo to form a continuous lipid film, which was dried in vacuo for 4 h. PBS was added to hydrate the film at a final conc. of 2 mM with vortexing at 55°C for 1–2 min. The resulting mixture was sonicated for 10 min, no pulsing, amplitude at 90%, using an ice bath; 1200 μL of 2 mM MalLNP formulation (containing 0.24 μmol MalLipid **5**) was conjugated to Cys‐BAGE4_18–39_ (0.48 μmol, 2.0 eq.) in the presence of an excess of TCEP (2 eq. over peptide), at pH 7. The reaction mixture was agitated at rt for 3 h. The sample was loaded into Slide‐A‐Lyzer Dialysis Cassette (10K MWCO) then placed in dialysate buffer (PBS), 500× the volume of loaded sample. The dialysate buffer was changed after 2 h, 3 times, and then allowed to dialyse overnight. The sample was recovered and analysed by UV–Vis spectroscopy at 280 nm.

### 2.4 LNP Characterization

CysBAGE4‐MalLNP and **2**‐LNP formulations were analysed using DLS, circular dichroism (CD), and TEM. DLS analysis: CysBAGE4‐MalLNP and **2**‐LNP formulations were diluted with Milli‐Q water and transferred to disposable cuvettes before measurement at 25°C with a 173° light scattering angle, wavelength range: 180–280 nm. CD analysis: CysBAGE4‐MalLNP and **2**‐LNP formulations were diluted with Milli‐Q water and analysed using a quartz cell with a 0.01 mm path length. Spectra were measured using a 0.5 nm step, 1 nm bandwidth and 1 s collection time per step. TEM analysis: CysBAGE4‐MalLNP and **2**‐LNP formulations were diluted in Milli‐Q water and applied to glow‐discharged carbon‐coated copper 200 mesh grids and negative‐stained with 2% uranyl acetate.

### 2.5 ELISA and Competitive ELISA Assays

General methods for the ELISA and competitive ELISA assays can be found in the Supporting Information.

### 2.6 In Vivo Evaluation

Animal experiments were carried out with ethical approval from University of Nottingham ethical review board and under a Home Office approved project license (PP2706800). Mice were dosed with 10 nmol of material in 50 μL at Days 1, 8 and 15 (*n* = 3). IFNϒ ELISpot assays on splenocytes were conducted on termination at Day 21. General methods for the ELISpot can be found in the Supporting Information. Preparation of media and buffers and steps performed on Days 1 and 2 post‐termination; the ELISpot assays were performed using a laminar flow cabinet and aseptic techniques to ensure the sterility of media, reagents and plates at stages before development of the ELISpot. Development of the ELISpot on Day 4 posttermination was performed on the laboratory bench.

## 3 Results and Discussion

### 3.1 Synthesis of Pam_2_Cys‐Modified CTAs (1–3)

Two CTA epitopes; NY‐ESO‐1_157‐165_ and BAGE4_18–39_ were employed as the antigenic components in our studies. NY‐ESO‐1_157‐165_ is spontaneously immunogenic and able to bind to HLA‐A2, expressed by a wide range of cancers [34, 35, 36]. This epitope is known to reactivate T‐cell responses (CD8+ cells) in models vaccinated against NY‐ESO‐1 [34, 35, 36]. BAGE4_18–39_ represents the entire BAGE4 CTA minus the signal peptide; this antigen is expressed in 22% of melanomas and 30% of infiltrating bladder carcinomas [29]; to our knowledge, it has not been evaluated in vivo. Inclusion of the SK_4_ solubility tag within our vaccine constructs enables formulation of the Pam_2_Cys peptides in buffer for administration [31, 37].

In light of Brimble's SAR studies on Pam_2_Cys, and to avoid possible compromises in activity, no modifications or deviations from the Pam_2_Cys moiety were explored, and the essential ester linkage was used in the construction of palmitoylated antigens. Native (*R*)‐stereochemistry has been retained throughout the routes applied as the (*S*)‐analogue of the Pam_2_Cys adjuvanting moiety exhibits a hundredfold decrease in potency compared to the (*R*)‐analogue [19]. An additional benefit of using palmitoyl functionality is the ability of the fatty acid chains to be incorporated into nanoparticles via self‐assembly, which we have utilised in this study. The three synthetic approaches explored (Figure 1) enable the effective on‐resin synthesis of the vaccine (approaches 2 and 3) as well as investigations into the use of an electrophilic moiety that could be employed to introduce the Pam_2_Cys adjuvant into peptide and protein antigens via late‐stage conjugation (approach 1).

**SCHEME 1:**
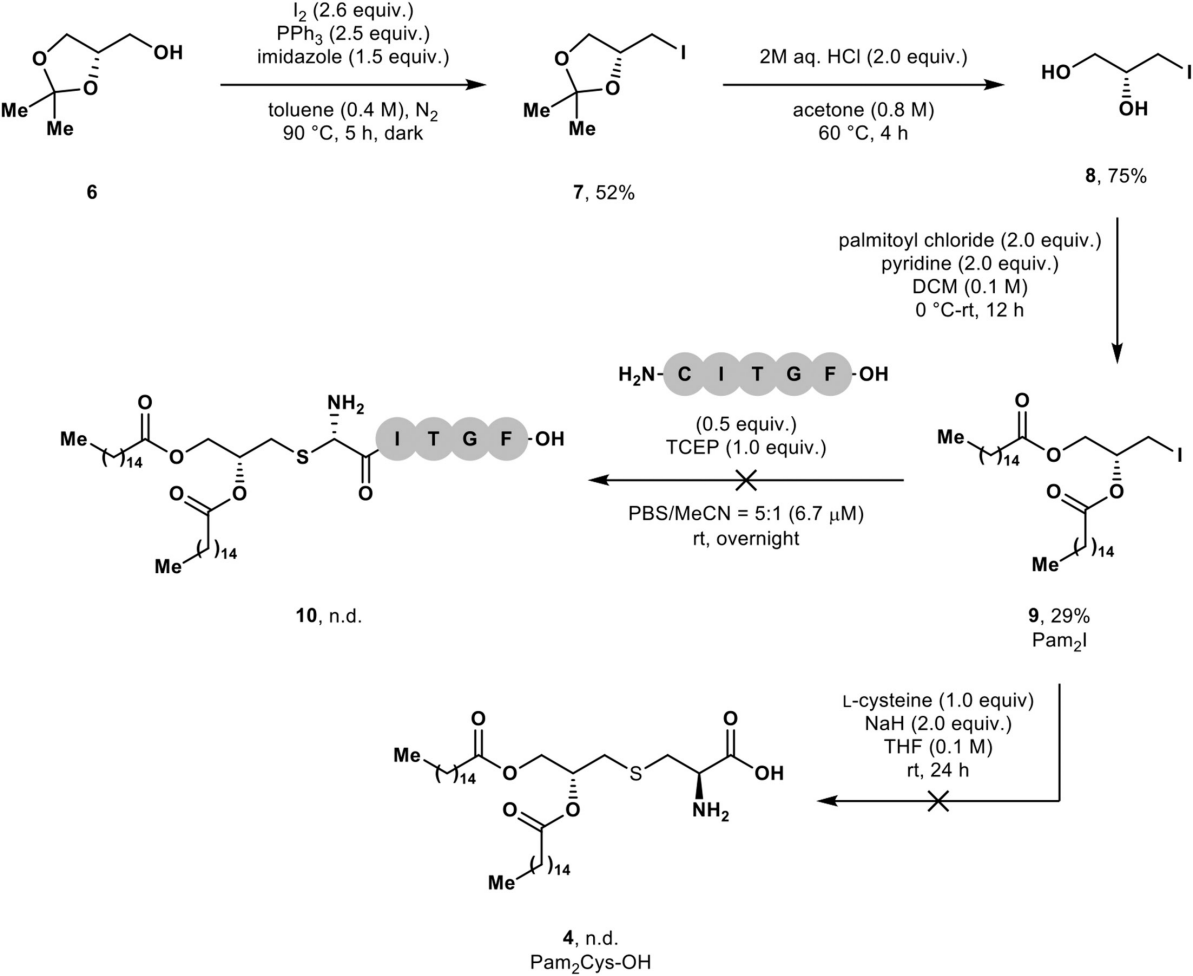
Attempted synthesis of peptide‐adjuvant conjugate **10** via alkylation approach.

Our initial approach towards the target vaccine constructs involved the synthesis of alkylating agent, Pam_2_I **9**, outlined in Scheme 1. Briefly, Pam_2_I **9** was prepared from solketal **6** in three steps, beginning with the preparation of alkyl iodide **7** under Garegg–Samuelsson conditions [38]. The acetal protecting group was then removed under acidic conditions to furnish diol **8**, which was palmitoylated to yield Pam_2_I **9** in an 11% yield over three steps. Compound **9** could potentially be a powerful reagent for the installation of the Pam_2_Cys adjuvant into peptides and proteins carrying an N‐terminal Cys residue. Unfortunately, the alkylation of model peptide H‐CITGF‐OH was unsuccessful; no conversion of the starting peptide was observed. This is attributed to the mismatch in solubility between alkyl iodide **9** and the peptide in both organic and organic/aqueous solvent mixtures. Attempts to prepare key building block **4** via this route were also unsuccessful. In addition to the recalcitrant nature of the conjugation reaction, isomerisation of the alkyl iodide, akin to that observed by Brimble et al. for similar substrates [17], cannot be ruled out, which would further complicate the utilisation of building block **9**. Any production of the *S* isomer of the glyceryl unit either in the peptide conjugate or amino acid **4** would diminish the activity of the vaccine construct [19, 28].

**SCHEME 2:**
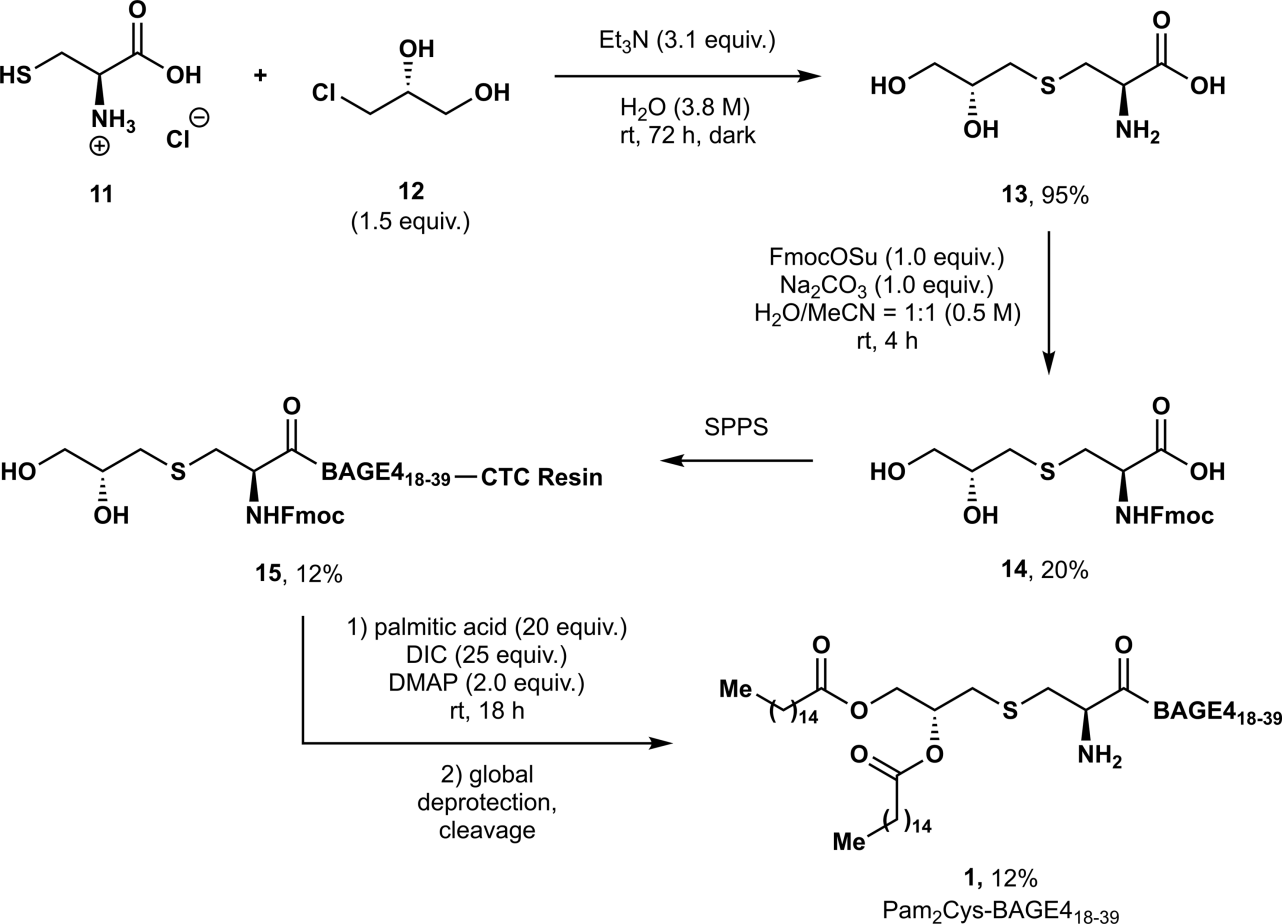
Synthesis of Pam_2_Cys‐BAGE4_18–39_ **1**.

An alternative approach to the synthesis of the palmitoylated antigen targets via late‐stage esterification on‐resin was also considered (Scheme 2). Alkylation of cysteine hydrochloride **11** with α‐chlorohydrin **12** yielded diol **13** in an excellent yield of 95%, which was taken to the next step without further purification. Treatment of **13** with Fmoc‐OSu produced Fmoc‐protected amino acid **14**, which was then installed at the N‐terminus of antigenic peptide BAGE4_18–39_ via standard SPPS (**15**). Installation of the palmitoyl chains was completed using a Steglich esterification between the resin‐bound peptide and palmitic acid [39]. The adjuvant‐peptide conjugate was cleaved from the resin and globally deprotected to furnish the desired product Pam_2_Cys‐BAGE4_18–39_ **1** in a 12% yield over the final coupling and esterification steps.

**SCHEME 3:**
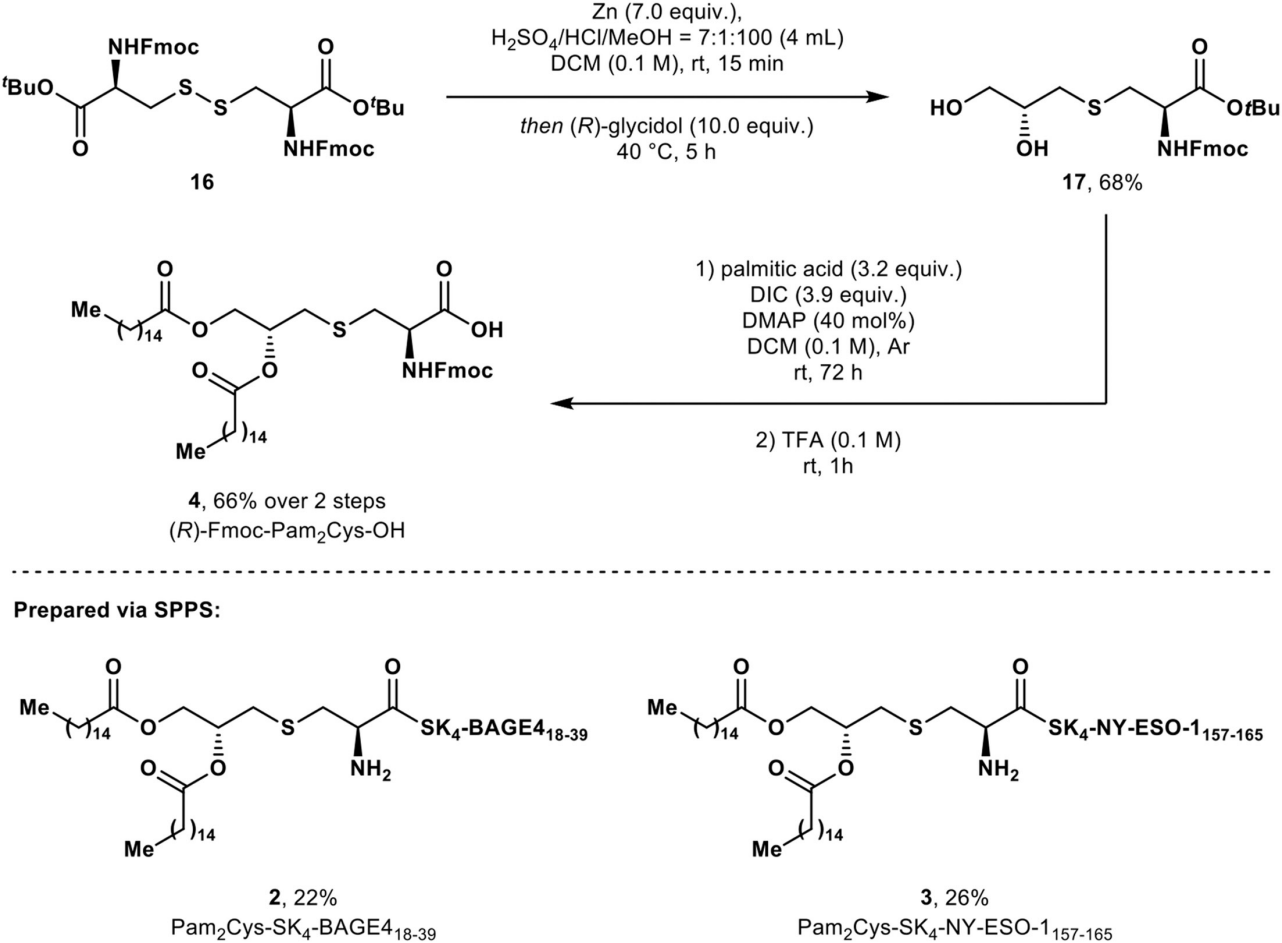
Top: Synthesis of (*R*)‐Fmoc‐Pam_2_Cys‐OH **4**. Bottom: vaccine‐adjuvant conjugates **2** and **3** prepared from amino acid **4**.

This successful route to Pam_2_Cys‐BAGE4_18–39_ **1** was applied to the synthesis of adjuvant‐vaccine conjugates **2** and **3**, both of which carry the solubility sequence SK_4_; however, the final palmitoylation step tended to yield mixtures of the desired *bis*‐palmitoylated product with impurities consistent with the formation of *mono*‐palmitoylated products. To avoid this complication, targets **2** and **3** were prepared via the installation of palmitoylated amino acid Fmoc‐Pam_2_Cys‐OH **4**. Since the synthesis of **4** using Pam_2_I **9** was unsuccessful, an alternative route was sought (Scheme 3).

The preparation of (*R*)‐Fmoc‐Pam_2_Cys‐OH **6** was completed in three steps, beginning with *S*‐alkylation of Fmoc‐Cys‐O^*t*^Bu **16** with (*R*)‐glycidol to yield diol **17**. Palmitoylation and deprotection of **17** afforded the desired product **4** in a 45% yield over three steps. Although routes to Fmoc‐Pam_2_Cys‐OH **4** have been reported from Fmoc‐Cys‐OH [19, 40], the chosen route from commercially available Fmoc‐Cys‐O^*t*^Bu **16** shortens the synthetic route by two steps. Using amino acid **4**, two vaccine‐adjuvant constructs were prepared—Pam_2_Cys‐SK_4_‐BAGE4_18–39_ **2** and Pam_2_Cys‐SK_4_‐NY‐ESO‐1_157‐165_ **3**. Rink amide resin (yielding a C‐terminal primary amide) was used in the synthesis of vaccine‐adjuvant constructs **2** and **3**, after improved overall yields for SPPS were achieved compared to synthesis on 2‐chlorotrityl chloride (2‐CTC) resin (yielding the carboxylate). Moreover, C‐terminal amidation, a common posttranslational modification (PTM) observed widely across the proteome, confers enhanced stability in vivo due to resistance to enzymatic degradation and, in many cases, enhances binding affinity [41].

### 3.2 Evaluation of Vaccine Secondary Structure and Fibril Formation

Interrogation of the structure of peptide‐adjuvant constructs **2** and **3** was performed using CD spectroscopy as well as modelling studies (PEP‐FOLD) [42, 43]. The CD spectra (Figures S12 and S13) show that both compounds form α‐helical structures, however, NY‐ESO‐1_157‐165_ **3** exhibits significantly more α‐helical character (*f*_α_ = 0.41) than Pam_2_Cys‐SK_4_‐BAGE4_18–39_ **2** (*f*_α_ = 0.07), which PEP‐FOLD indicates has a disordered C‐terminal domain (see Supporting Information for details). SAXS and cryo‐TEM studies (Figures S16–S18) show that both constructs form fibrils (Supporting Information). Fibril formation by α‐helical peptides is typically observed due to lateral association of coiled‐coils [44]. Here, this may play a role, although lateral interaction of the hydrophobic alkyl chains is likely to be the essential driver for the fibril formation. The fibril core radius 14.0–18.5 Å from SAXS (Table S1) is consistent with the length of an extended Pam lipid chain.

### 3.3 In Vitro Evaluation of BAGE4_18–39_ Antigen and Pam_2_Cys‐SK_4_‐BAGE4_18–39_ (2)

**FIGURE 2:**
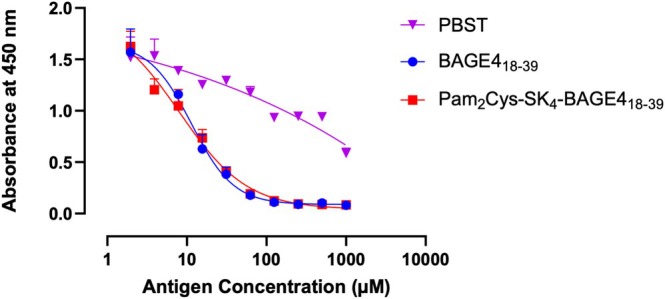
ELISA data to compare the binding affinity of BAGE4_18–39_ antigen IC_50_ = 11.41 μM +/−3.52, and Pam_2_Cys‐SK_4_‐BAGE4_18–39_ **2** IC_50_ = 8.04 μM +/−6.04, PBST buffer with 0.05% Tween 20 as negative control, absorbance measured at 450 nm.

To confirm antibody recognition of the BAGE4_18–39_ antigen when incorporated into the vaccine construct **2** the primary antibody to this antigen (anti‐BAGE4 antibody, produced in rabbit; Sigma Aldrich SAB4301150) was incubated with Pam_2_Cys‐SK_4_‐BAGE4_18–39_ **2** and peptide BAGE4_18–39_ at 11 different concentrations between 1000 and 0.98 ng/mL (PBST as the negative control). Antibody–antigen complexes were then added to 384‐well plates which were precoated with 10 ng/mL of antigen. The secondary antibody, specific to the primary and conjugated to horseradish peroxidase (HRP) enzyme (produced in rabbit), was added, followed by TMB for colour development. The binding affinity of Pam_2_Cys‐SK_4_‐BAGE4_18–39_ **2** gave an IC_50_ value of 8.04 μM; BAGE_18–39_ alone gives an IC_50_ value of 11.41 μM (Figure 2). Thus, we can be confident that the covalent grafting of the Pam_2_Cys adjuvant does not affect recognition of the antigen.

### 3.4 In Vivo Evaluation of BAGE4 Peptide‐Adjuvant Construct

To compare the T‐cell responses induced by the BAGE4_18–39_ peptide (administered with IFA as the adjuvant) and Pam_2_Cys‐SK_4_‐BAGE4_18–39_ **2**, groups of healthy mice were immunized on three occasions (1, 8 and 15 days) with 10 nmol of each conjugate via s.c injection (0.2 mM dose concentration). The response was measured by count of peptide‐specific IFNγ‐secreting T‐cells by ELISpot assay. Since no studies to date have addressed T‐cell responses to BAGE4 in conventional or HLA‐A2 (HHDII/DR1) transgenic mice, conventional mice were initially selected. The H‐2d haplotype (BALB/c) strain was selected for this study as the epitope predictions for peptides that bind to MHC I and II are effective for this strain (IEDB Analysis Resource). Splenocytes from the immunised mice were tested against three predicted epitope peptides from BAGE4_18–39_, which should reactivate T‐cell responses in this strain of mice when immunised with BAGE4_18–39_ vaccine candidates. Lipopolysaccharide (LPS; 5 μg/mL) was used as the positive control (non‐specific stimulus of the immune system).

**FIGURE 3:**
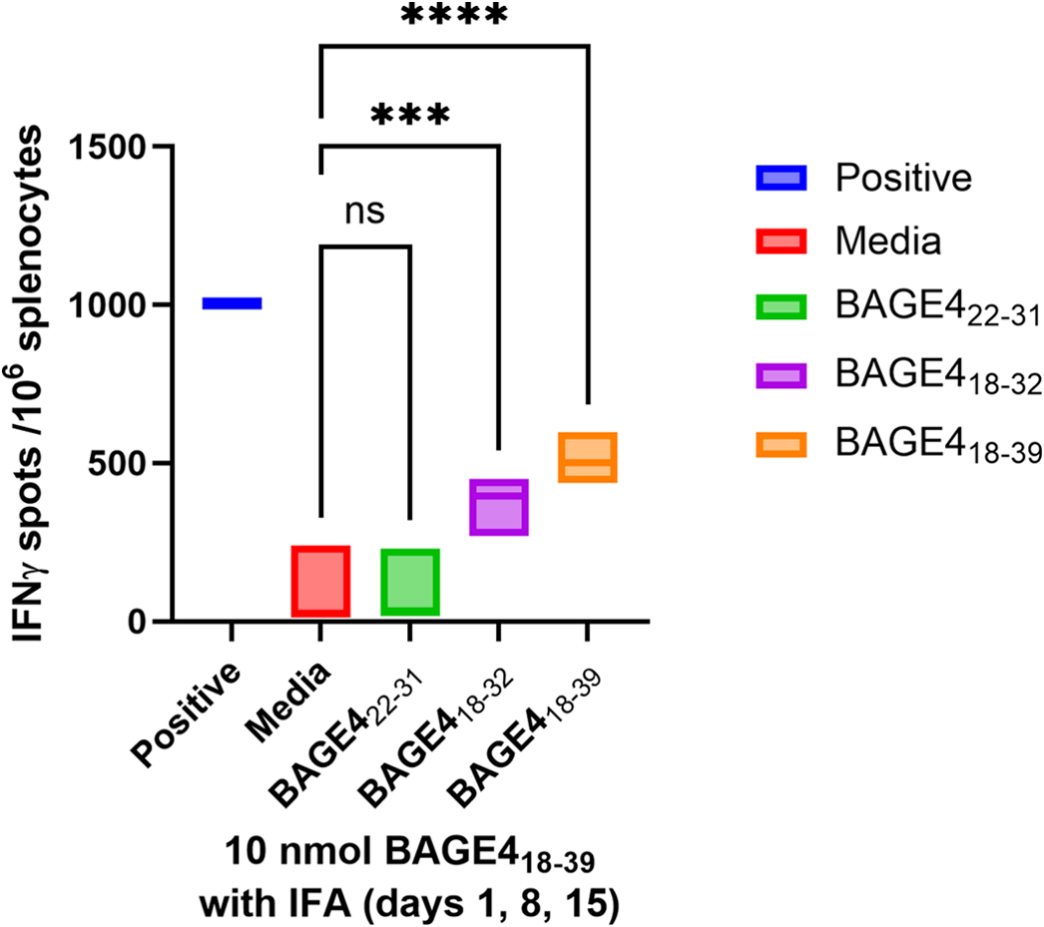
Immune responses induced in BALB/c mice immunized with BAGE4_18–39_ peptide mixed with IFA. Isolated splenocytes from the immunised mice tested against three peptides from the BAGE4_18–39_ peptide and measured in IFNγ ELISpot assay.

The BAGE4_18–39_ sequence was observed to be immunogenic in conventional BALB/c mice, stimulating T‐cell responses that recognise BAGE4 with good responses to the whole native BAGE4_18–39_ sequence (median = 511) and slightly lower responses to the BAGE4_18–32_ peptide (Figure 3). There is almost no response to BAGE4_22–31_; a one‐way ANOVA statistical test was carried out and confirmed a significant effect for BAGE4_18–39_ (*p* = 0.9999, q = 0.02932, DF = 8) and BAGE4_18–32_ (*p* = 0.0009, q = 6.142, DF = 8), while BAGE4_22–31_ shows no significance (*p* = > 0.9999, q = 0.02932, DF = 8).

Unfortunately, mice dosed with Pam_2_Cys‐SK_4_‐BAGE4_18–39_ **2** reacted negatively and were culled on Day 1. There was significant bleeding and darkening at the injection site and the liver appeared pale and patchy coloured. Repeating the experiment using a 20‐fold decrease in concentration of **2** produced the same result. Since the negative in vivo reaction upon administration of **2** was apparent within 24 h of vaccine administration, immune‐mediated toxicity is unlikely which typically takes longer than 24 h to manifest effects [45, 46]. The BAGE4_18–39_ antigen **8** alone does not show any toxicity and Pam_2_Cys is a well‐studied and safe adjuvant; [45, 46] thus, further investigation is required to elucidate the mechanism(s) behind this unanticipated toxicity.

### 3.5 Formulation of Antigen‐Loaded LNPs

**FIGURE 4:**
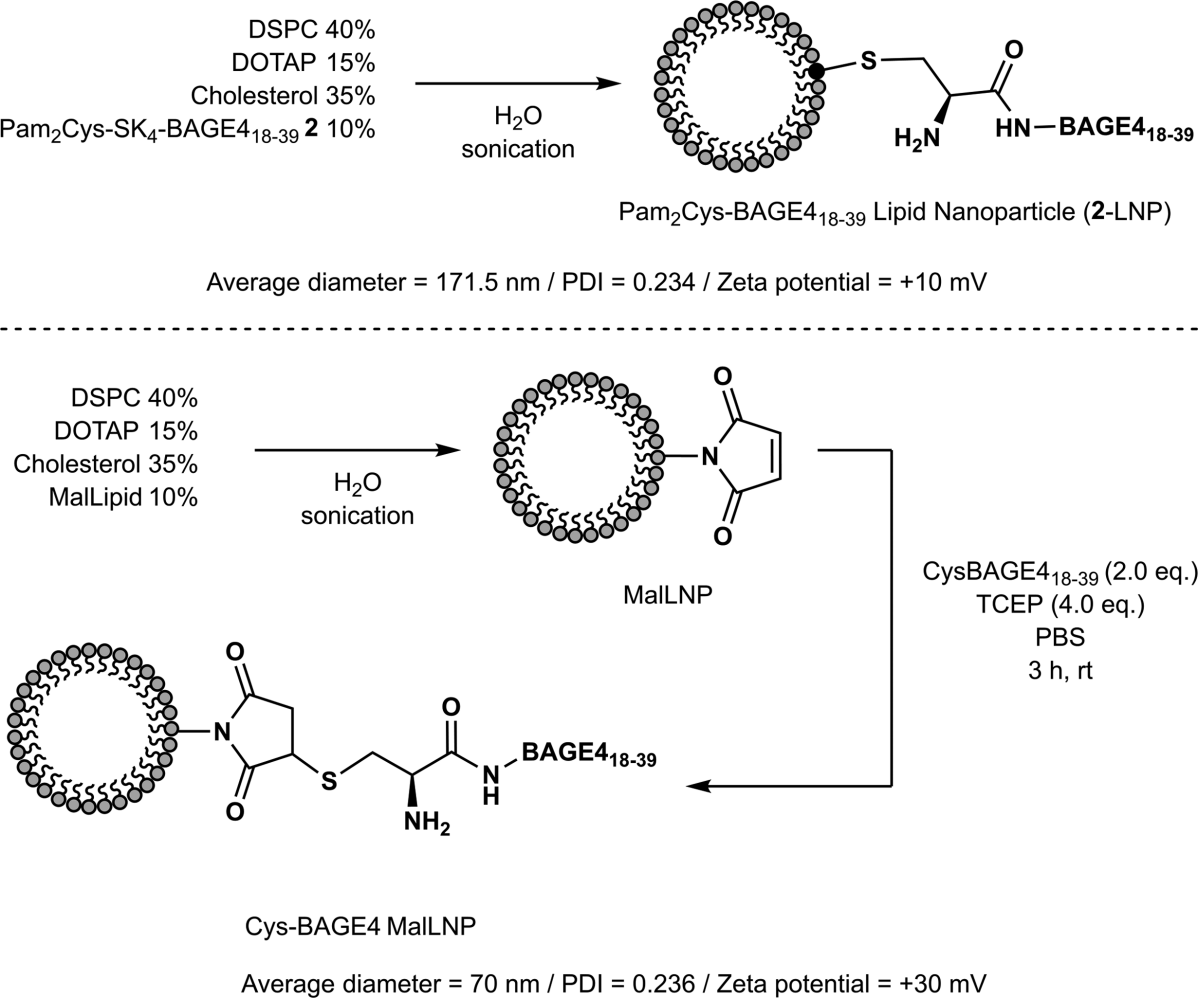
Formulation of nanoparticles Pam_2_Cys‐BAGE4_18–39_ LNP (**2**‐LNP) and Cys‐BAGE4‐MalLNP used in this study. DSPC = 1,2‐dipalmitoyl‐*sn*‐glycero‐3‐phosphocholine. DOTAP = 1,2‐dioleoyl‐3‐trimethylammonium propane. LNPs formulated as multilamellar vesicles.

In addition to the synthesis and evaluation of the palmitoylated antigens administered alone, we also explored the formulation of the vaccine construct **2** into LNPs and the conjugation of the BAGE4 CTA onto the outer envelope of a LNP for in vivo delivery, a method often employed for targeted nucleic acid delivery (Figure 4) [47, 48]. To date, most reported LNP vaccines have been formulated by antigen entrapment [49]. However, antigen‐entrapped liposomes and surface‐coupled antigens of liposomes are reported to induce different types of immune responses [50]. Antigen‐entrapped liposomes have been shown to induce antigen‐specific IgE antibody production [51], while antigens coupled to the surface of liposomes induced substantial IgG antibody production with a minimal amount of IgE antibody production as shown by ovalbumin‐liposome [52], tetanus toxoid [53] or Shiga‐like toxin [54], coupled to the outer envelope of LNPs. Antigen‐LNP conjugates are therefore considered to be suitable vaccine candidate strategies that cause minimal allergic reaction [55]. Nanoparticles were formulated using either covalent linkage of Cys‐BAGE4_18–39_ to maleimide‐containing LNPs (CysBAGE4‐MalLNP, using a bespoke maleimide lipid (**5**)), or self‐assembly of LNPs from Pam_2_Cys‐SK_4_‐BAGE4_18–39_ **2** (**2**‐LNP), and in vivo assays were conducted.

Optimization of the lipid and cholesterol ratios allows the size distribution and stability of the LNP formulations to be ‘tuned’ [56]. Nanoparticles of diameter 100–200 nm, PDI < 0.3 and zeta potentials (ζ) of > ±10 mV were deemed desirable, and formulations containing 35%–40% cholesterol were used throughout. A cationic liposome formulation was selected, as anionic liposomes quickly engage with the biological system after becoming opsonized by circulating protein [57, 58], resulting in the rapid uptake by the reticuloendothelial system (RES) and toxic effects such as pulmonary hypertension, dyspnea and a drop in circulating platelets and leukocytes [59]. A range of lipid compositions that would yield stable cationic LNPs within the appropriate diameter range were evaluated; the optimum composition was found to be 40 mol% DSPC, 15 mol% DOTAP, 35 mol% cholesterol and either 10 mol% MalLipid **5** [33] or 10 mol% Pam_2_Cys‐SK_4_‐BAGE4_18–39_ **2**. These LNPs were formulated in Milli‐Q water via the thin film hydration method [60], and 10 vol% (final volume) of 10x PBS solution was added after sonication to afford the LNPs in PBS (2 mM); the samples were then analysed by TEM and DLS. The MalLNP sample (including repeats to ensure consistency) gave average particle diameters of 70 nm (PDI 0.236) and a zeta potential (ζ) of +30 mV. Storage of the solution for 36 days at 4°C and reanalysis after this time showed little deviation from these values indicating acceptable stability. A control formulation, which omitted the MalLipid **5** (45 mol% DSPC, 15 mol% DOTAP, 40 mol% cholesterol; non‐MalLNP), was also prepared (diameter 83 nm, PDI 0.26, zeta potential +35 mV). The formulation made up with 10 mol% Pam_2_Cys‐SK_4_‐BAGE4_18–39_ **2** (**2**‐LNP) afforded particles with an average diameter of 165 nm (PDI 0.3) and a zeta potential of +10 mV.

To prepare CysBAGE4‐MalLNPs, a solution of MalLNP (100 μL of a 2 mM formulation containing 0.02 µmol of MalLipid **5**), 5 equiv. of CysBAGE4_18–39_ and 10 equiv. of TCEP was agitated for 3 h then purified via dialysis. UV–Vis analysis showed the successful loading of 0.06 μmol of peptide, while the control reaction using NonMal‐LNPs demonstrated 0.02 μmol of loading (see Supporting Information for details). Due to the cationic nature of the MalLNPs (+ 30 mV) and the net negative charge of the CysBAGE4_18–39_ peptide at pH 7.0, electrostatic association of the peptide with the LNPs was anticipated; however, significantly more loading was observed for the MalLNPs than expected considering the NonMal‐LNP control. Thus, to minimise electrostatic loading, 2 equiv. of CysBAGE4_18–39_ (in the presence of 2 equiv. of TCEP relative to peptide) was employed for the CysBAGE4‐MalLNP samples intended for in vivo evaluation.

Competitive ELISA for the CysBAGE4‐MalLNP conjugate shows reduced competition relative to the BAGE4_18–39_ antigen (IC_50_ = 151.4 μM and IC_50_ = 11.41 μM, respectively, Figure S26), comparable with the negative control. This result may be explained by ineffective antigen presentation on the surface of the particles due to the formation of higher order multilayered liposomes (i.e., the antigen is buried within the lipid (bi)layers) [61], or ineffective coating of the plates with the LNP sample.

### 3.6 In Vivo Evaluation of Antigen‐Loaded LNPs

**FIGURE 5:**
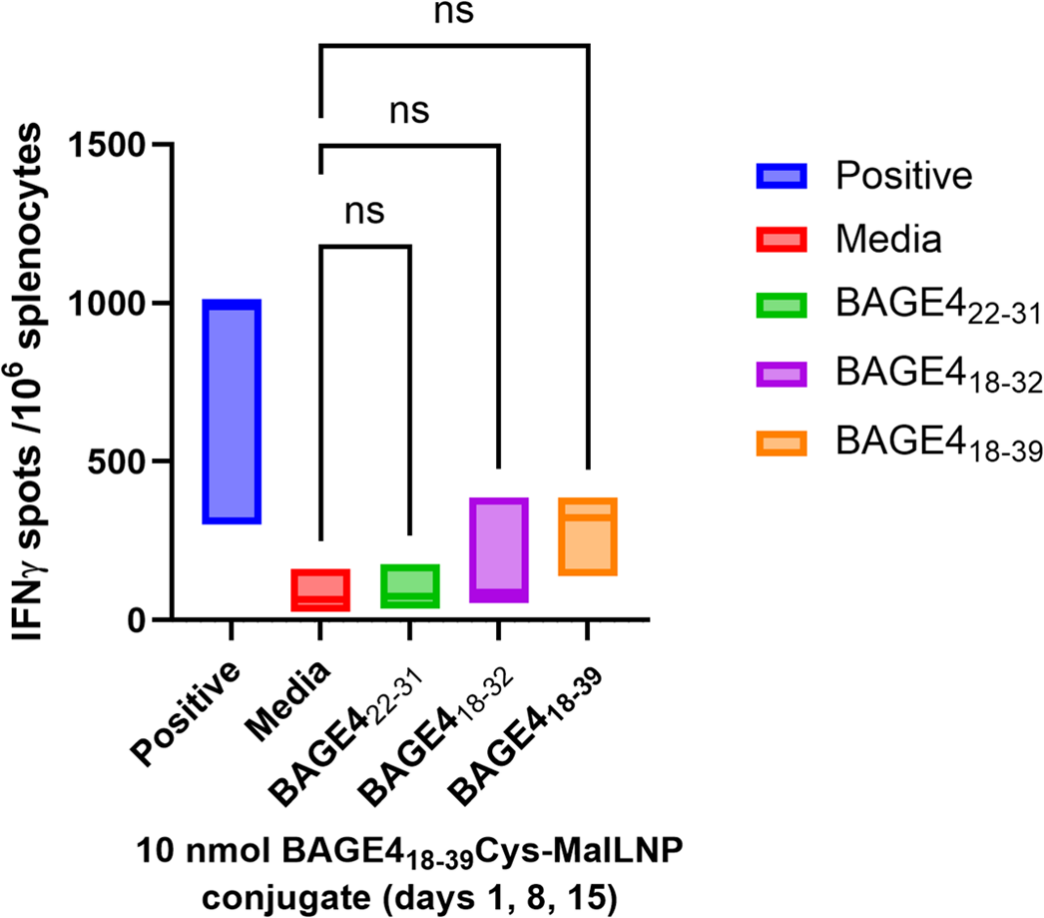
Immune responses induced in BALB/c mice immunized with CysBAGE4‐MalLNPconjugate. Isolated splenocytes from the immunised mice tested against three peptides from the BAGE4_18–39_ peptide and measured in IFNγ ELISpot assay.

In vivo evaluation of the antigen‐loaded LNPs was conducted as described in Section 3.4. The CysBAGE4‐MalLNP formulation will serve as an informative control, demonstrating the response of the peptide and nanoparticle without adjuvant. The immune response for this peptide‐LNP conjugate was somewhat varied (385, 139, 323, with median = 282). A one‐way ANOVA statistical test was carried out and confirmed no significant effect for BAGE4_18–39_ (*p* = 0.5013, q = 1.359, DF = 8), BAGE4_18–32_ (*p* = 0.9165, q = 0.6330, DF = 8) & BAGE4_22–31_ (*p* > 0.9999, q = 0.08425, DF = 8) due to high variability between repeats (Figure 5).

The muted immune response to the delivery of CysBAGE4‐MalLNP was to be expected as the bespoke MalLipid **5** is unlikely to act as an effective adjuvant due to deviation in chemical structure relative to Pam_2_Cys [62]. As was the case with dosing of Pam_2_Cys‐SK_4_‐BAGE4_18–39_ **2**, mice dosed with Pam_2_Cys‐SK_4_‐BAGE4_18–39_ self‐assembled liposomes (**2**‐LNP) reacted negatively and were culled on Day 1 of administration.

### 3.7 In Vivo Evaluation of Pam_2_Cys‐NY‐ESO‐1 Peptide‐Adjuvant Construct

**FIGURE 6:**
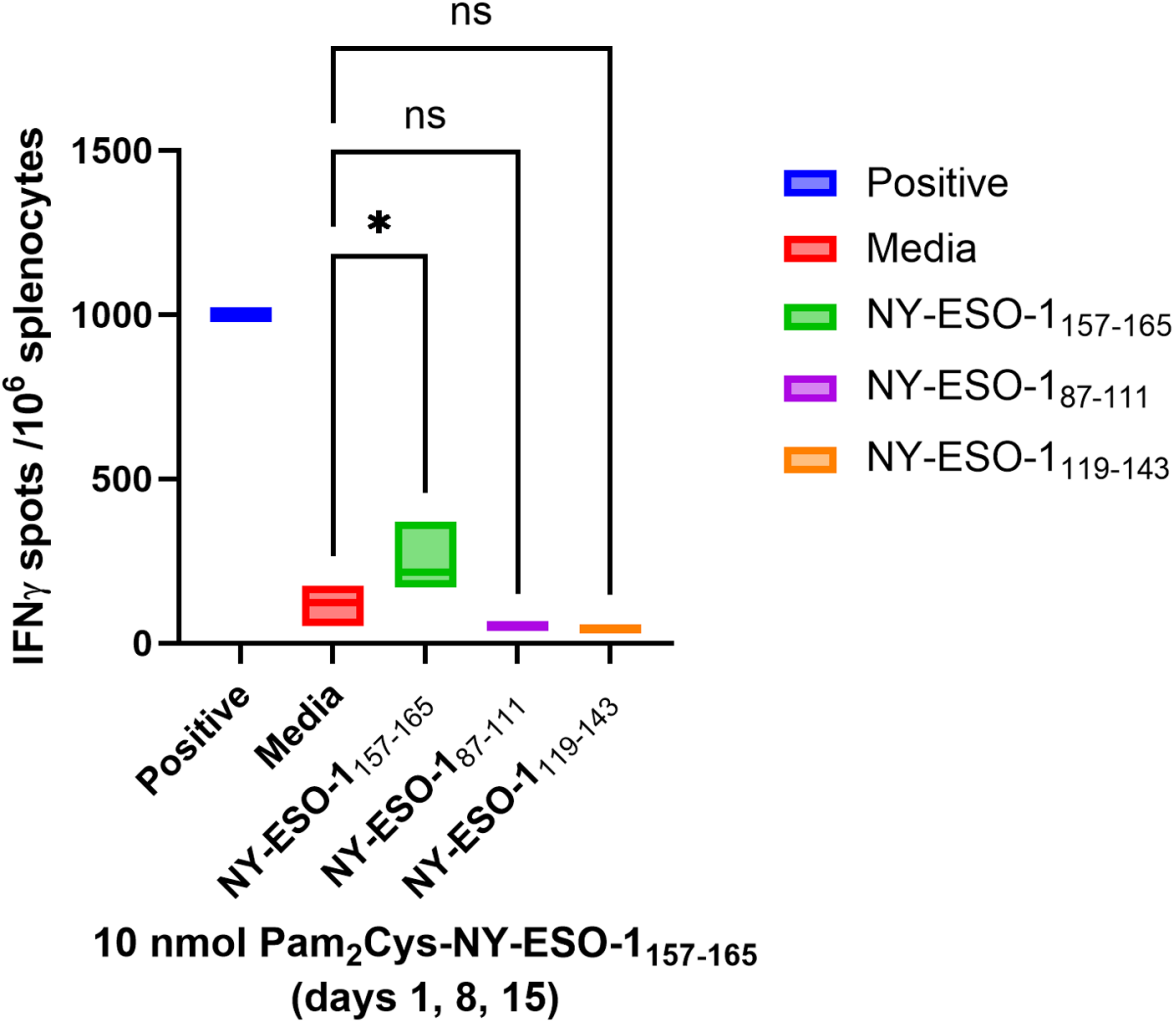
Immune responses induced in HHDII/DR1 mice immunized with the Pam_2_Cys‐SK_4_‐NY‐ESO‐1_157‐165_ **3** vaccine candidate. Isolated splenocytes from the immunised mice tested against three peptides from the NY‐ESO‐1 protein, NY‐ESO‐1_157‐165_, NY‐ESO‐1_87‐111_ & NY‐ESO‐1_119‐143_, and measured in IFNγ ELISpot assay.

In vivo evaluation of Pam_2_Cys‐SK_4_‐NY‐ESO‐1_157‐165_ **3** in healthy HLA‐A2 transgenic mice demonstrated specific but weak responses for **3** over the media‐only negative control (Figure 6). A one‐way ANOVA statistical analysis was carried out to assess the significance of the effect of Pam_2_Cys‐SK_4_‐NY‐ESO‐1_157‐165_ **3** conjugate on the immune response of mice and confirmed a significant effect for NY‐ESO‐1_157‐165_ (*p* < 0229, q = 3.567, DF = 8), while the data for both NY‐ESO‐1_87‐111_ (*p* = 0.3280, q = 1.704, DF = 8) and NY‐ESO‐1_119‐143_ (*p* = 0.2293, q = 1.969, DF = 8) were not significant.

Confirmation that Pam_2_Cys‐SK_4_‐NY‐ESO‐1_157‐165_ **3** is weakly immunogenic is a promising development towards the construction of a synthetic vaccine based on this antigen and adjuvant combination. However, further engineering of the vaccine is required to increase the potency of this candidate before proceeding to cancer models.

## 4 Conclusion

Herein, we report the synthesis of peptide‐adjuvant constructs Pam_2_Cys‐BAGE4_18–39_ **1**, Pam_2_Cys‐SK_4_‐BAGE4_18–39_ **2** and Pam_2_Cys‐SK_4_‐NY‐ESO‐1_157‐165_ **3** and demonstrate that Pam_2_Cys‐SK_4_‐NY‐ESO‐1_157‐165_ **3** shows activity as a self‐adjuvanting vaccine candidate in the first in vivo studies of this scaffold. LNPs were formulated via self‐assembly of constituent lipids, which included Pam_2_Cys‐SK_4_‐BAGE4_18–3_ **2**, and via conjugation of Cys‐BAGE4_18–39_ to the outer envelope of LNPs containing a maleimide lipid (MalLipid **5**), and explored as a delivery tool, antigen display scaffold and adjuvant (**2**‐LNP). The unexpected in vivo toxicity of both Pam_2_Cys‐SK_4_‐BAGE4_18–39_ **2** and **2**‐LNP is of interest, considering the components of these vaccines are non‐toxic when administered separately. Immune‐mediated toxicity is an unlikely factor due to the rapid onset of symptoms in these models. Whilst fibril formation was observed for Pam_2_Cys‐SK_4_‐BAGE4_18–39_ **2**, Pam_2_Cys‐SK_4_‐NY‐ESO‐1_157‐165_ **3** also formed fibrils and was found to be non‐toxic. Localization of the peptide is likely to be dramatically altered by palmitoylation, which may result in toxicity. However, further clarity regarding the activity of BAGE4 (a secreted peptide with evidence at transcriptional level only) would be required to draw any substantive conclusions. Due to the promising results obtained from the Pam_2_Cys‐SK_4_‐NY‐ESO‐1_157‐165_ **3** scaffold, future work will involve the integration of additional components into this vaccine (such as T‐helper epitopes) to further enhance the potency of this self‐adjuvanting vaccine construct.

## Conflicts of Interest

Lindy Durrant is the director of Scancell; Victoria Brentville and Peter Symonds are employees of Scancell.

## Supporting information


**Figure S1.** Analytical HPLC trace of crude H‐CITGF‐OH. Analytical gradient 10%–60% B over 5 min 0.6 mL/min, 210 nm. HRMS *m/z* calc. [M + H] + 540.2492, obs. [M + H] + 540.2514.
**Figure S2.** Analytical HPLC trace of pure H–BAGE418–39–OH. Analytical gradient 2%–95% B over 5 min 0.6 mL/min, 280 nm. ESI + MS *m/z* calc. [M + 2H]2 + 1286.6413, [M + 3H]3 + 858.0966, obs. [M + 2H]2 + 1287.1618, [M + 3H]3 + 858.4298.
**Figure S3.** Analytical HPLC trace of pure H–BAGE418–39–NH2. Analytical gradient 2%–95% B over 5 min 0.6 mL/min, 280 nm. ESI + MS *m/z* calc. [M + 2H]2 + 1286.1493, [M + 2H + Na]3 + 865.0959, obs. [M + 2H]2 + 1286.6605, [M + 2H + Na]3 + 865.7713.
**Figure S4.** Analytical HPLC trace of pure H–NY‐ESO‐1157‐165–NH2. Analytical gradient 10%–100% B over 5 min 0.6 mL/min, 280 nm. HRMS *m/z* calc. [M + H] + 1093.5387, obs. [M + H] + 1093.5494.
**Figure S5.** Analytical HPLC trace of pure H–SK4‐NY‐ESO‐1157‐165–NH2. Analytical gradient 2%–95% B over 5 min 0.6 mL/min, 280 nm. ESI + MS *m/z* calc. [M + H] + 1692.9652, [M + 2H]2 + 846.9862, obs. [M + H] + 1693.9300, [M + 2H]2 + 847.4935.
**Figure S6.** Analytical HPLC trace of pure H–CysBAGE418–39–NH2. Analytical gradient 2%–95% B over 5 min 0.6 mL/min, 280 nm. ESI + MS *m/z* calc. [M + 2H]2 + 1337.6538, [M + 3H]3 + 892.1050, obs. [M + 2H]2 + 1338.1601, [M + 3H]3 + 892.4426.
**Figure S7.** Analytical HPLC trace of pure H–BAGE418–32–NH2. Analytical gradient 2%–95% B over 10 min 0.6 mL/min, 280 nm. ESI + MS *m/z* calc. [M + H] + 1887.9893, [M + 2H]2 + 944.4929, obs. [M + H] + 1888.9491, [M + 2H]2 + 944.9962.
**Figure S8.** Analytical HPLC trace of pure H–BAGE423–31–NH2. Analytical gradient 2%–60% B over 10 min 0.6 mL/min, 280 nm. HRMS *m/z* calc. [M + H] + 1101.5655, obs. [M + H] + 1101.5610.
**Figure S9.** Analytical HPLC trace of pure Pam2Cys‐BAGE418–39 (**1**). Analytical gradient 2%–100% B over 10 min, 280 nm. ESI + MS *m/z* calc. [M + 2H]2 + 1613.3939, [M + 3H]3 + 1075.9317, obs. [M + 2H]2 + 1613.8979, [M + 3H]3 + 1076.2668.
**Figure S10.** Analytical HPLC trace of pure Pam2Cys‐SK4‐BAGE418–39 (**2**). Analytical gradient 2%–100% B over 10 min, 280 nm. ESI + MS *m/z* calc. [M + 2H]2 + 1912.6078, [M + 3H]3 + 1275.4076, [M + 4H]4 + 956.8076, obs. [M + 2H]2 + 1913.5783, [M + 3H]3 + 1276.0767 [M + 4H]4 + 957.3195.
**Figure S11.** Analytical HPLC trace of pure Pam2Cys‐SK4‐NY‐ESO‐1157‐165 (**3**). Analytical gradient 10%–100% B over 5 min, 280 nm. ESI + MS *m/z* [M + 2H]2 + 1173.7389, [M + 3H]3 + 782.8283, obs.[M + 2H]2 + 1174.2336, [M + 3H]3 + 783.1668.
**Figure S12.** CD spectrum recorded for Pam2Cys‐SK4‐BAGE418–39 (**2**).
**Figure S13.** CD spectrum recorded for Pam2Cys‐SK4‐NY‐ESO‐1157‐165 (**3**).
**Figure S14.** Modelling data generated by PEPFOLD for SK4‐BAGE418–39, showing disordered C‐terminal domain. Red: α‐helix. Blue: disordered. Green: ‘extended’.
**Figure S15.** Modelling data generated by PEPFOLD for SK4‐NY‐ESO‐1157‐165. Red: α‐helix. Blue:disordered. Green: ‘extended’.
**Figure S16.** Cryo‐TEM images for 1 wt% aqueous solution of Pam2Cys‐SK4‐BAGE418–39 (**2**).
**Figure S17.** Cryo‐TEM images for 1 wt% aqueous solution of Pam2Cys‐SK4‐NY‐ESO‐1157‐165 (**3**).
**Figure S18.** Small Angle X‐Ray Scattering (SAXS) data for 1 wt% solutions of peptides Pam2Cys‐SK4‐BAGE418–39 (**2**) and Pam2Cys‐SK4‐NY‐ESO‐1 (**3**). Data fitted with core‐shell cylinder form factor and indicate the formation of fibrils. The SAXS fitting parameters are listed in Table S1.
**Table S1.** SAXS fit parameters using a core‐shell cylinder form factor.
**Figure S19.** Analytical HPLC trace of Fmoc‐(*R*)‐(2,3‐dihydroxypropyl)cysteine (**14**). Analytical gradient 30% isocratic for 14 min; 0.6 mL/min, 280 nm.
**Table S2.** Summary of the reagents, mass, and volume used to formulate Pam2Cys‐SK4‐BAGE418–39 **2**‐LNP (2 mM, 1 mL).
**Table S3**: Summary of the reagents, mass, and volume used to formulate MalLNP (2 mM, 2.5 mL).
**Table S4**: Quantification of electrostatic peptide loading.
**Figure S20.** DLS spectra of MalLNP size distributions by intensity. Z‐Average (d.nm): 70.28, PdI: 0.236; Zeta potential of +30 mV.
**Figure S21.** DLS spectra of Pam2Cys‐SK4‐BAGE418–39–NH2 LNP (**2**‐LNP) size distributions by intensity. Z‐Average (d.nm): 164.5, PdI: 0.304; Zeta potential of +10 mV.
**Figure S22.** TEM for MalLNP, the image capturing was operated at 43 k.
**Figure S23.** TEM for Cys‐BAGE4‐MalLNP the image capturing was operated at 43 k.
**Figure S24.** ELISA binding curves for BAGE418–39 with a serial dilution series of Anti‐BAGE4 antibody, absorbance measured at 450 nm.
**Figure S25.** ELISA binding curves for NY‐ESO‐1157‐165 with a serial dilution series of Anti‐NY‐ESO‐1 antibody, absorbance measured at 450 nm.
**Figure S26.** ELISA data to compare the binding affinity of BAGE418–39 antigen IC50 = 11.41 mM +/−3.52, Pam2Cys‐SK4‐BAGE418–39 (**2**) IC50 = 8.04 mM +/−6.04 Cys‐BAGE4‐MalLNP IC50 = 151.4 mM, PBST buffer with 0.05% Tween 20 as negative control.
**Table S5.** Summary of the injection schedule (including dose concentration and number) for Pam2Cys‐SK4‐NY‐ESO‐1157‐165 (**2**) candidate using HHDII/DR1 Tg mice.
**Table S6.** Summary of the injection schedule (including dose concentration and number) for the following vaccine candidates: BAGE418–39 candidate (coadministered with IFA), Cys‐BAGE4‐MalLNP conjugate, Pam2Cys‐SK4‐BAGE18–39 (**2**), and **2**‐LNP using BALB/c mice.

## Data Availability

Data for this article including experimental details, compound and particle characterization data, in vitro and in vivo evaluation data are available in the ESI.
