# Supplementary material for: In Vivo Evaluation of Pam2Cys‐Modified Cancer‐Testis Antigens as Potential Self‐Adjuvanting Cancer Vaccines
Source: J Pept Sci. 2025 May 6;31(6):e70022. doi: 10.1002/psc.70022 (PMC12053792; doi:10.1002/psc.70022)
Supplement: Supplementary file 1 — Figure S1. Analytical HPLC trace of crude H‐CITGF‐OH. Analytical gradient 10%–60% B over 5 min 0.6 mL/min, 210 nm. HRMS m/z calc. [M + H] + 540.2492, obs. [M + H] + 540.2514. Figure S2. Analytical HPLC trace of pure H–BAGE418–39–OH. Analytical gradient 2%–95% B over 5 min 0.6 mL/min, 280 nm. ESI + MS m/z calc. [M + 2H]2 + 1286.6413, [M + 3H]3 + 858.0966, obs. [M + 2H]2 + 1287.1618, [M + 3H]3 + 858.4298. Figure S3. Analytical HPLC trace of pure H–BAGE418–39–NH2. Analytical gradient 2%–95% B over 5 min 0.6 mL/min, 280 nm. ESI + MS m/z calc. [M + 2H]2 + 1286.1493, [M + 2H + Na]3 + 865.0959, obs. [M + 2H]2 + 1286.6605, [M + 2H + Na]3 + 865.7713. Figure S4. Analytical HPLC trace of pure H–NY‐ESO‐1157‐165–NH2. Analytical gradient 10%–100% B over 5 min 0.6 mL/min, 280 nm. HRMS m/z calc. [M + H] + 1093.5387, obs. [M + H] + 1093.5494. Figure S5. Analytical HPLC trace of pure H–SK4‐NY‐ESO‐1157‐165–NH2. Analytical gradient 2%–95% B over 5 min 0.6 mL/min, 280 nm. ESI + MS m/z calc. [M + H] + 1692.9652, [M + 2H]2 + 846.9862, obs. [M + H] + 1693.9300, [M + 2H]2 + 847.4935. Figure S6. Analytical HPLC trace of pure H–CysBAGE418–39–NH2. Analytical gradient 2%–95% B over 5 min 0.6 mL/min, 280 nm. ESI + MS m/z calc. [M + 2H]2 + 1337.6538, [M + 3H]3 + 892.1050, obs. [M + 2H]2 + 1338.1601, [M + 3H]3 + 892.4426. Figure S7. Analytical HPLC trace of pure H–BAGE418–32–NH2. Analytical gradient 2%–95% B over 10 min 0.6 mL/min, 280 nm. ESI + MS m/z calc. [M + H] + 1887.9893, [M + 2H]2 + 944.4929, obs. [M + H] + 1888.9491, [M + 2H]2 + 944.9962. Figure S8. Analytical HPLC trace of pure H–BAGE423–31–NH2. Analytical gradient 2%–60% B over 10 min 0.6 mL/min, 280 nm. HRMS m/z calc. [M + H] + 1101.5655, obs. [M + H] + 1101.5610. Figure S9. Analytical HPLC trace of pure Pam2Cys‐BAGE418–39 (1). Analytical gradient 2%–100% B over 10 min, 280 nm. ESI + MS m/z calc. [M + 2H]2 + 1613.3939, [M + 3H]3 + 1075.9317, obs. [M + 2H]2 + 1613.8979, [M + 3H]3 + 1076.2668. Figure S10. Analytical HPLC trace of pure Pa [file PSC-31-e70022-s001.pdf]

In Vivo Evaluation of Pam_2Cys-Modified Cancer-Testis Antigens as Potential Self-Adjuvanting Cancer Vaccines

Salwa Aljohani,[a,⊥] Alex G. Edmonds,[a] Valeria Castelletto,[b] Jani Seitsonen,[c] Ian Hamley,[b] Peter Symonds,[d] Victoria A. Brentville,[d] Lindy G. Durrant,[d] and Nicholas J. Mitchell*[a]

[a]School of Chemistry, University of Nottingham, University Park, Nottingham, NG7 2RD, United Kingdom

[b]School of Chemistry, Pharmacy and Food Biosciences, University of Reading, Reading, RG6 6AH, United Kingdom

[c]Nanomicroscopy Center, Aalto University, FIN-02150 Espoo, Finland

[d]Scancell, Biodiscovery Institute, University of Nottingham, University Park, Nottingham, NG7 2RD, United Kingdom

[⊥]Present address: College of Science, Department of Chemistry, Taibah University, Yanbu, 46522, Saudi Arabia

Supporting Information

Contents

Contents 2

Materials 3

General Methods 4

Solid Phase Peptide Synthesis (SPPS) 6

Manual Fmoc-SPPS 6

Automated Fmoc-SPPS 7

Peptide Synthesis 8

Pam_2Cys–Antigen Synthesis 16

General Procedure for Coupling Pam_2Cys to Peptide Antigen (Protocol A) 16

General Procedure for Coupling Pam_2Cys to Peptide Antigen (Protocol B) 16

Structural Evaluation of Peptides 20

Synthesis of Organic Compounds 29

NMR Spectra 38

Synthesis of Lipid Nanoparticles 44

Determination of Electrostatic Loading of LNPs 45

LNP Characterisation 46

Evaluation of Biological Activity 48

ELISA assay 48

Competitive ELISA 50

In Vivo Evaluation (ELISpot Assay) 51

ELISpot Assay 52

References 55

Materials

All commercially available reagents and reagent-grade solvents were purchased from Merck, Fluorochem or Fisher, and used as received unless otherwise stated. Amino acids, coupling reagents and resins were obtained from Novabiochem, Fluorochem or GL Biochem. Reagents that were not commercially available were synthesised as reported herein. Antibodies were purchased from Sigma Aldrich. All aqueous solutions were prepared using deionised water. Dry solvents were used when indicated in the procedure.

General Methods

NMR samples were analysed on either a Bruker AVIII 400 NMR system (^{1}H-NMR frequency 400 MHz; ^{13}C-NMR frequency 101 MHz) or a Bruker 500 MHz system (^{1}H-NMR frequency 500 MHz; ^{13}C-NMR frequency 125 MHz). Chemical shifts are reported in parts per million (ppm) and are referenced to solvent residual signals: $CDCl_3$ (δ 7.26 [^{1}H]), DMSO (δ 2.50 [^{1}H]), MeOD (δ 3.31 [^{1}H]). ^{1}H NMR data is reported as chemical shift (δ), multiplicity (s = singlet, d = doublet, t = triplet, q = quartet or combinations of these splitting patterns; m = unassigned multiplet), relative integral, and coupling constant (*J*, Hz). ^{13}C NMR data is reported as chemical shift (δ) and classification of the carbon (e.g., CH_3/CH_2/CH/C).

High-resolution mass spectra were recorded on a Bruker MicroTOF Focus II MS (ESI) operating in positive or negative ionisation mode. Analytical HPLC was performed on a Thermo Ultimate 3000 mHPLC system equipped with PDA eλ detector (λ = 210 – 400 nm). Peptides were analyzed using a Waters Sunfire 5 μm, 2.1 x 150 mm column (C-18) at a flow rate of 0.6 mL/min. The mobile phase composed of 0.1% trifluoroacetic acid in H_2O (Solvent A) and 0.1% trifluoroacetic acid in acetonitrile (Solvent B). The analysis of the chromatograms was conducted using Chromeleon 7 software.

Preparative reverse-phase HPLC was performed using a Waters 1525 binary pump HPLC equipped with a dual wavelength UV detector set to 210 nm and 280 nm. Peptides were purified on a Waters Sunfire 5 μm, 19 x 150 mm (C-18) preparative column operating at a flow rate of 6 mL/min using a mobile phase of 0.1% trifluoroacetic acid in water (Solvent A) and 0.1% trifluoroacetic acid in acetonitrile (Solvent B) using the gradient specified. Semi-preparative reverse-phase HPLC was performed using the same HPLC and solvent system. For peptides, a Waters Sunfire 5 μm, 10 x 250 mm (C-18) preparative column was used, operating at a flow rate of 5 mL/min using the gradient specified. Preparative reverse-phase HPLC of lipo-peptides **1-3** was performed using a Waters Sunfire 5 μm, 10 x 150 mm (C-4) semi-preparative column operating at a flow rate of 5 mL/min using the gradient specified.

Dynamic Light Scattering (DLS) Spectra were recorded using a Zetasizer Nano ZS (Malvern Panalytical) to measure the particle size and charge. Samples were diluted x10 with Milli-Q water and transferred to disposable cuvettes before measurement at 25 °C with a 173° light scattering angle.

Circular Dichroism (CD) Spectra were recorded using a Chirascan spectropolarimeter (Applied Photophysics, U.K.). Peptide solutions were mounted in a Quartz cell with detachable

windows, with 0.01 mm path length. Spectra were measured using a 0.5 nm step, 1 nm bandwidth and 1 s collection time per step, and a range of 180 to 280 nm. The CD signal from the water background was subtracted from the CD data of solutions. The background corrected CD spectra were smoothed using the Chirascan Software (4.2.27) for data analysis. The residue of the calculation was chosen to oscillate around the average, to avoid artifacts in the smoothed curve. CD data, measured in mdeg, was normalized to molar ellipticity using the molar concentration of the sample and the cell path length.

Cryogenic-TEM (Cryo-TEM) Imaging was carried out using a field emission cryo-electron microscope (JEOL JEM-3200FSC), operating at 200 kV. Images were taken in bright field mode and using zero loss energy filtering (omega type) with a slit width of 20 eV. The micrographs were recorded using a Gatan Ultrascan 4000 CCD camera. The specimen temperature was maintained at -187 °C during the imaging. Vitrified specimens were prepared using an automated FEI Vitrobot device using Quantifoil 3.5/1 holey carbon copper 200 mesh grids with a hole size of 3.5 μm and negative-stained with 2% uranyl acetate. Just prior to use, grids were plasma cleaned using a Gatan Solarus 9500 plasma cleaner and then transferred into the environmental chamber of a FEI Vitrobot at room temperature and 100 % humidity. Thereafter 3 μl of sample solution (2 mM; approx. 2 mg/mL) was applied on the grid and it was blotted twice for 5 seconds and then vitrified in a 1/1 mixture of liquid ethane and propane at temperature of -180 °C. The grids with vitrified sample solution were maintained at liquid nitrogen temperature and then cryo-transferred to the microscope.[1]

Small-angle X-ray scattering experiments (SAXS) SAXS experiments were performed on beamline B21 at Diamond (Didcot, UK).[2] The sample solutions were loaded into the 96-well plate of an EMBL BioSAXS robot and then injected via an automated sample exchanger into a quartz capillary (1.8 mm internal diameter) in the X-ray beam. The quartz capillary was enclosed in a vacuum chamber, to avoid parasitic scattering. After the sample was injected into the capillary and reached the X-ray beam, the flow was stopped during the SAXS data acquisition. Beamline B21 was operated with a fixed camera length (3.9 m) and fixed energy (12.4 keV). The images were captured using a PILATUS 2M detector. Data processing was performed using dedicated beamline software ScÅtter.

Solid Phase Peptide Synthesis (SPPS)

Manual Fmoc-SPPS

Resin preloading, manual iterative peptide assembly, and cleavage reactions were conducted in fritted syringes (Torviq, USA). Syringes were capped and agitated on a rotating table at room temperature during coupling. Syntheses were carried out on a 0.25 mmol scale unless otherwise stated; loading of the resin was 0.5–0.8 mmol/g.

Preloading Rink Amide Resin

Rink Amide resin was initially washed with DCM (5 × 3 mL) followed by removal of the Fmoc group by treatment with 20% piperidine/DMF (2 × 5 min). The resin was washed with DMF (5 × 3 mL), DCM (5 × 3 mL) and DMF (5 × 3 mL). Oxyma Pure (4 eq.) and DIC (4 eq.) were added to a solution of Fmoc-AA-OH (4 eq.) in DMF. After 5 min of pre-activation, the mixture was added to the resin. After 2 h the resin was washed with DMF (5 × 3 mL), DCM (5 × 3 mL) and DMF (5 × 3 mL), capped with acetic anhydride/pyridine (1:9 v/v) (2 × 3 min) and washed with DMF (5 × 3 mL), DCM (5 × 3 mL) and DMF (5 × 3 mL).

Preloading 2-Chlorotrityl Chloride Resin

2-Chlorotrityl chloride resin was swollen in DCM for 30 min then washed with DCM (2 × 3 mL). A solution of Fmoc-AA-OH (0.5 equiv. relative to resin functionalization) and $i\mathrm{Pr}_2\mathrm{NEt}$ (2.0 eq. relative to resin functionalization) in DCM (final concentration 0.1 M of amino acid) was added and the resin shaken at rt for 16 h. The resin was washed with DMF (5 × 3 mL) and DCM (5 × 3 mL). The resin was treated with a solution of DCM/CH_3OH/$i\mathrm{Pr}_2\mathrm{NEt}$ (17:2:1 v/v/v, 3 mL) for 1 h and washed with DMF (5 × 3 mL), DCM (5 × 3 mL), and DMF (5 × 3 mL).

Estimation of Amino Acid Loading

The resin was treated with 20% piperidine/DMF (2 × 3 mL, 3 min) and 20 μL of the combined deprotection solution was diluted to 10 mL using 20% piperidine/DMF in a volumetric flask. The UV absorbance of the resulting piperidine-fulvene adduct was measured (λ = 301 nm, ε = 7800 M^{-1} cm^{-1}) to determine the loading of the resin.

General Amino Acid Coupling

A solution of protected amino acid (4 eq.), DIC (4 eq.) and Oxyma Pure (4 eq.) in DMF (final concentration 0.1 M) was added to the resin. After 1 h, the resin was washed with DMF (5 × 3 mL), DCM (5 × 3 mL) and DMF (5 × 3 mL).

Capping

Acetic anhydride/pyridine (1:9 v/v, 3 mL) was added to the resin. After 3 min the resin was washed with DMF (5 × 3 mL), DCM (5 × 3 mL) and DMF (5 × 3 mL).

Deprotection

The resin was treated with 20% piperidine/DMF (2 x 3 mL, 3 min) and washed with DMF (5 × 3 mL), DCM (5 × 3 mL) and DMF (5 × 3 mL).

Cleavage

A mixture of TFA, thioanisole, tri*iso*propylsilane (TIS) and water (90:4:4:2 v/v/v/v) was added to the resin. After 3 h, the resin was washed with TFA (3 × 2 mL).

Work-up

The combined cleavage solutions were concentrated under a stream of nitrogen to < 5 mL. 40 mL of diethyl ether was added to precipitate the peptide and the suspension centrifuged. The pellet was then dissolved in water containing 0.1% TFA, filtered and purified by preparative HPLC and analyzed by LC–MS and ESI mass spectrometry.

Automated Fmoc-SPPS

Automated Fmoc-SPPS was carried out on either a Biotage Initiator$^+$ Alstra or CEM Liberty Blue microwave peptide synthesizer. General synthetic procedures for Fmoc-deprotection and capping were carried out in accordance with the manufacturer's specifications. Biotage Initiator$^+$ Alstra: standardized amino acid couplings were performed for 15 min at 50 °C under microwave irradiation in the presence of amino acid (0.5 M in DMF, 4 eq.), Oxyma Pure (0.5 M in DMF, 4 eq.) and di*iso*propylcarbodiimide (0.5 M in DMF, 4 eq.). Peptide cleavage and work-up were carried out as described above for manual SPPS. CEM Liberty Blue: standardized amino acid couplings were performed for 2.5 min at 90 ºC under microwave irradiation in the presence of amino acid (0.2 M in DMF, 4 eq.), Oxyma Pure (1 M in DMF, 4 eq.) and di*iso*propylcarbodiimide (1 M in DMF, 4 eq.). Peptide cleavage and work-up were carried out as described above for manual SPPS.

Peptide Synthesis

The following peptides were synthesised using standard Fmoc-SPPS techniques as described above.

H–CITGF–OH was synthesized on 2-CTC resin (300 mg, 0.25 mmol). The crude peptide was cleaved and lyophilised to produce the desired peptide with no further purification necessary (121 mg, 0.22 mmol, 88% yield).

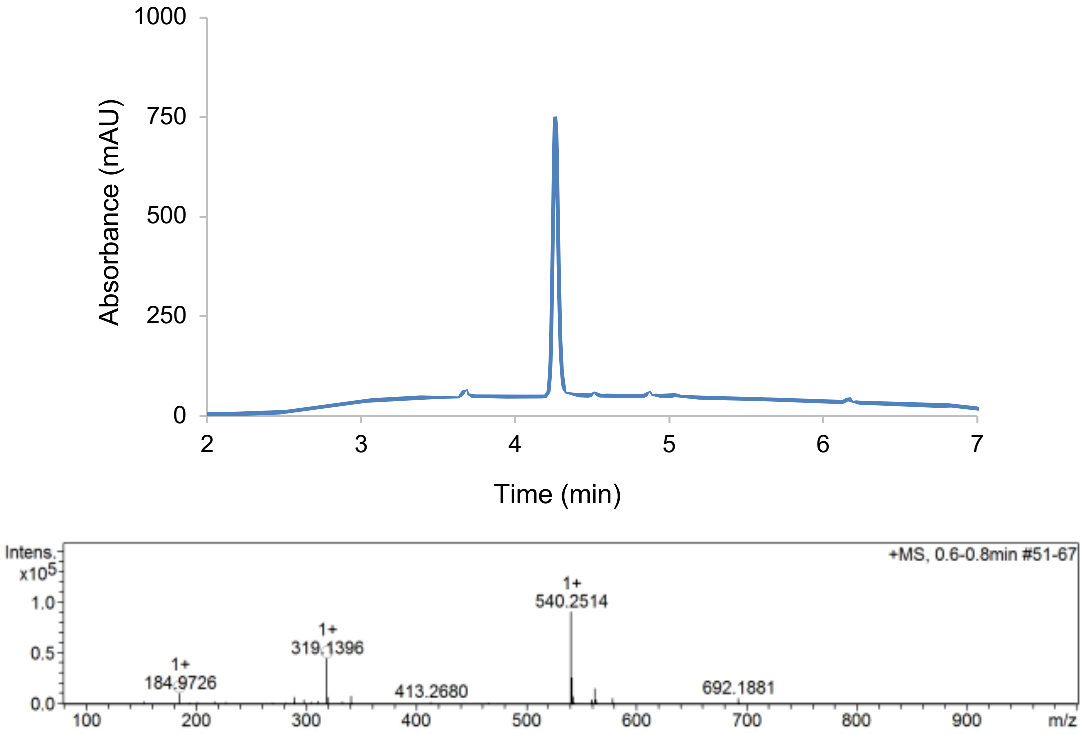

Figure S1. Analytical HPLC trace of crude H-CITGF-OH. Analytical gradient 10–60% B over 5 min 0.6 ml/min, 210 nm. HRMS *m/z* calc. $[M+H]^+$ 540.2492, obs. $[M+H]^+$ 540.2514.

H–BAGE4$_{18\text{-}39}$–OH = H–RLMKEESPVVSWWLEPEDGTAL–OH was synthesised on 2-CTC resin, 0.22 mmol. The crude peptide was cleaved and 0.05 mmol was purified by preparative HPLC (20 to 80% B over 30 min) and lyophilised to produce the desired peptide (20 mg, 0.0077 mmol, 15% yield).

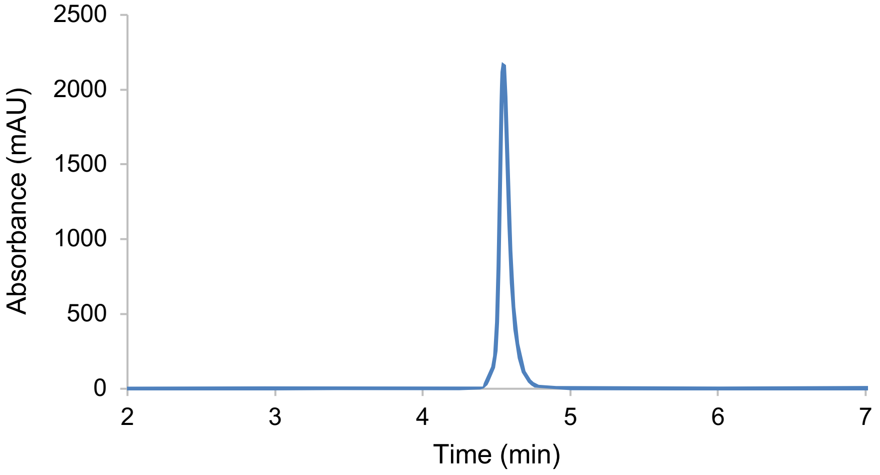

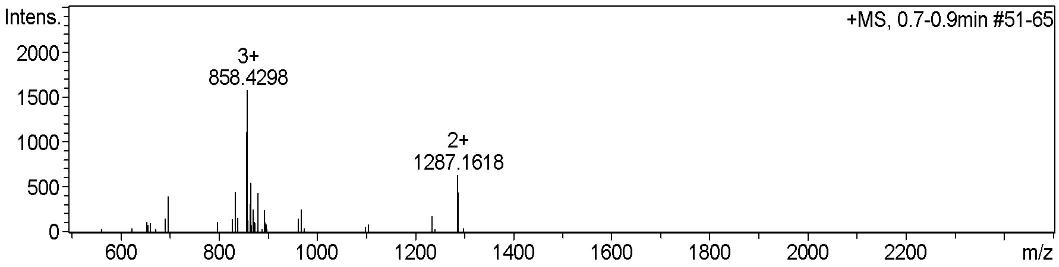

Figure S2. Analytical HPLC trace of pure H–BAGE4$_{18\text{-}39}$–OH. Analytical gradient 2–95% B over 5 min 0.6 ml/min, 280 nm. ESI$^+$ MS *m/z* calc. $[M+2H]^{2+}$ 1286.6413, $[M+3H]^{3+}$ 858.0966, obs. $[M+2H]^{2+}$1287.1618, $[M+3H]^{3+}$ 858.4298.

H–BAGE4$_{18\text{-}39}$–NH$_2$ = H–RLMKEESPVVSWWLEPEDGTAL–NH$_2$ was synthesised on Rink Amide resin, 0.18 mmol. The crude peptide was cleaved and 0.06 mmol was purified by preparative RP-HPLC (20 to 80% B over 30 min) and lyophilised to produce the desired peptide (30 mg, 0.0117 mmol, 20% yield).

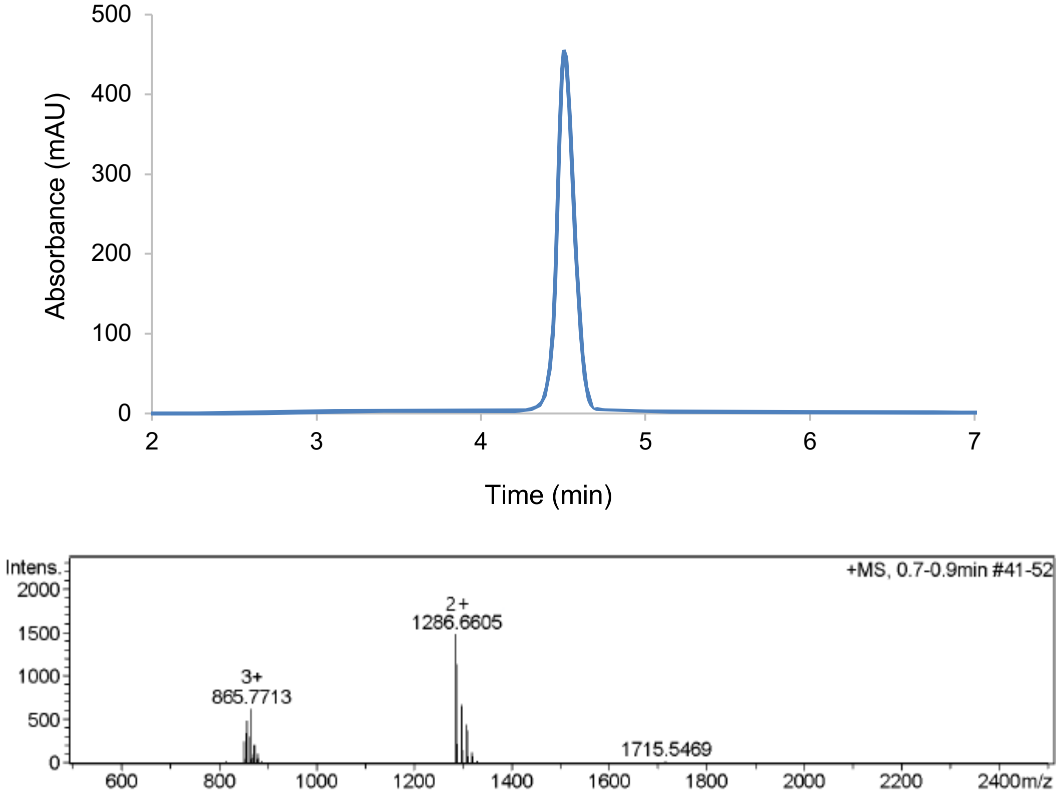

Figure S3. Analytical HPLC trace of pure H–BAGE4$_{18\text{-}39}$–NH$_2$. Analytical gradient 2–95% B over 5 min 0.6 ml/min, 280 nm. ESI$^+$ MS *m/z* calc. $[M+2H]^{2+}$ 1286.1493, $[M+2H+Na]^{3+}$ 865.0959, obs. $[M+2H]^{2+}$ 1286.6605, $[M+2H+Na]^{3+}$ 865.7713.

NY-ESO-1$_{157-165}$ = H–SLLMWITQC–NH$_2$ was synthesised on Rink Amide resin, 0.05 mmol. The crude peptide was cleaved and purified by preparative RP-HPLC (30 to 80% B over 35 min, isocratic @ 35% B for 5 min) and lyophilised to produce the desired peptide (11 mg, 0.0101 mmol, 20% yield).

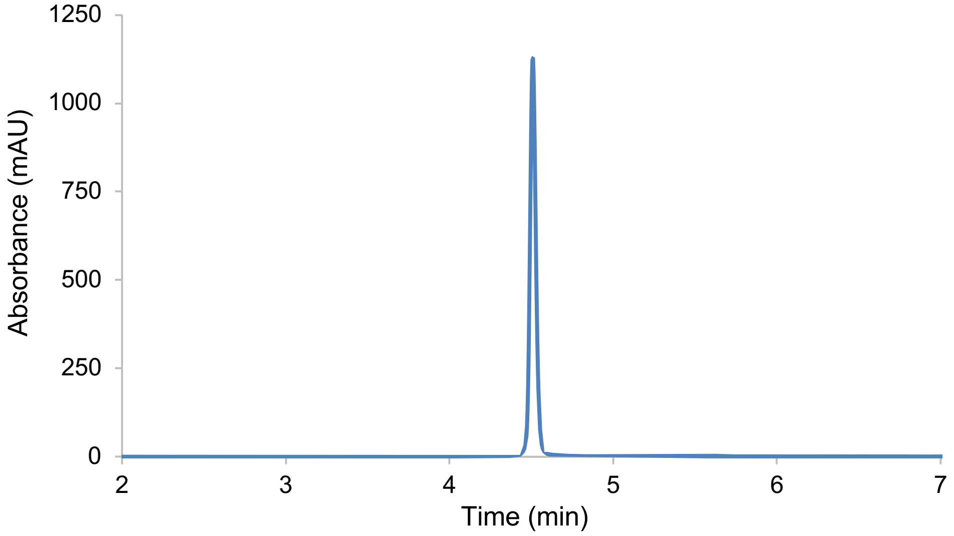

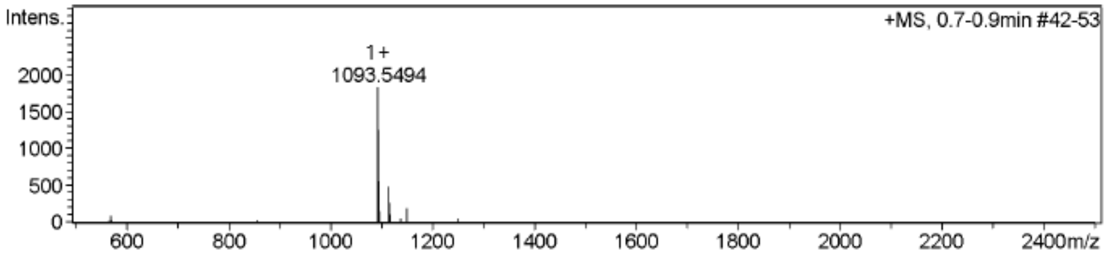

Figure S4. Analytical HPLC trace of pure H–NY-ESO-1$_{157-165}$–NH$_2$. Analytical gradient 10–100% B over 5 min 0.6 ml/min, 280 nm. HRMS *m/z* calc. $[M+H]^+$ 1093.5387, obs. $[M+H]^+$ 1093.5494.

SK_4-NY-ESO-$1_{157\text{-}165}$ = H–SKKKKSLLMWITQC–NH_2 was synthesised on Rink Amide resin, 0.05 mmol. The crude peptide was cleaved and purified by preparative RP-HPLC (20 to 70% B over 30 min) and lyophilised to produce the desired peptide (24 mg, 0.0142 mmol, 28% yield).

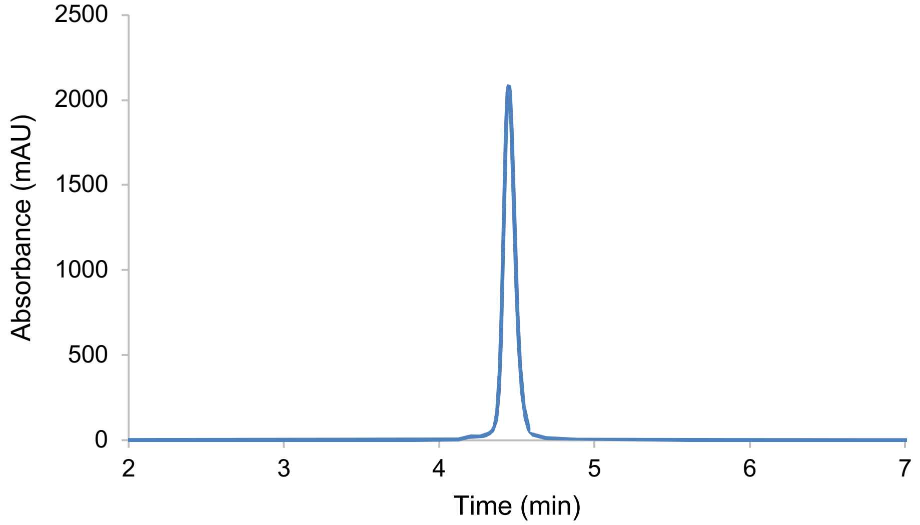

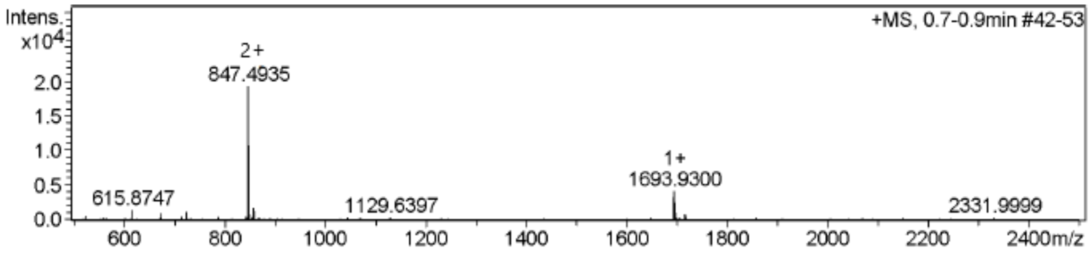

Figure S5. Analytical HPLC trace of pure H–SK_4-NY-ESO-$1_{157\text{-}165}$–NH_2. Analytical gradient 2–95% B over 5 min 0.6 ml/min, 280 nm. ESI^+ MS *m/z* calc. $[M+H]^+$ 1692.9652, $[M+2H]^{2+}$ 846.9862, obs. $[M+H]^+$ 1693.9300, $[M+2H]^{2+}$ 847.4935.

CysBAGE4$_{18\text{-}39}$ = H–CRLMKEESPVVSWWLEPEDGTAL–NH_2 was synthesised on Rink Amide resin, 0.10 mmol. The crude peptide was cleaved and 0.05 mmol was purified by preparative RP-HPLC (20 to 80% B over 30 min) and lyophilised to produce the desired peptide (25.5 mg, 0.0095 mmol, 19% yield).

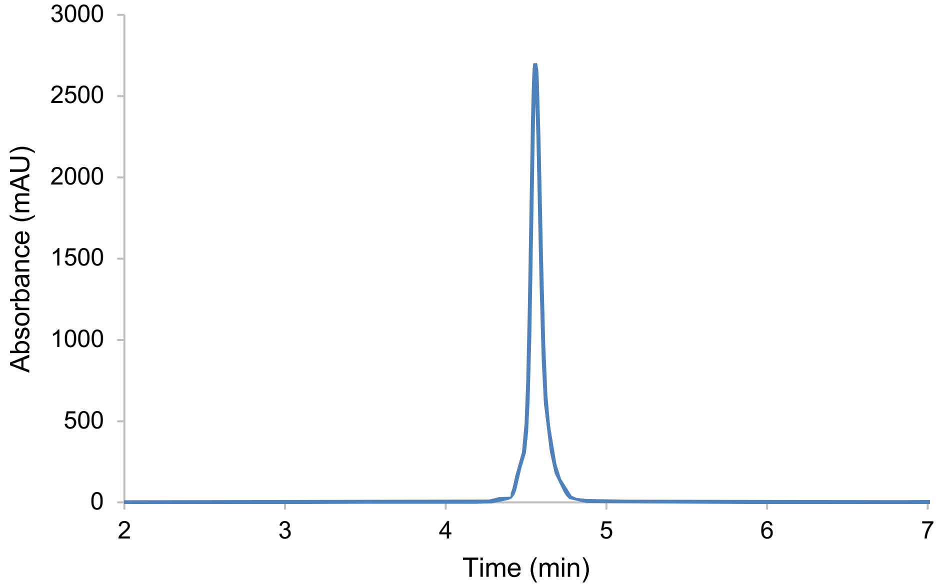

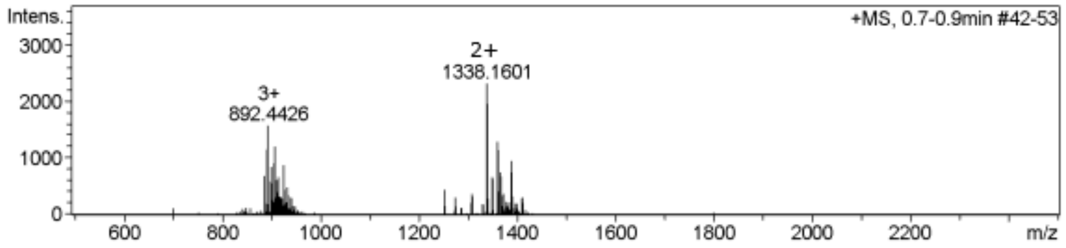

Figure S6. Analytical HPLC trace of pure H–CysBAGE4$_{18\text{-}39}$–NH_2. Analytical gradient 2–95% B over 5 min 0.6 ml/min, 280 nm. ESI$^+$ MS *m/z* calc. $[M+2H]^{2+}$ 1337.6538, $[M+3H]^{3+}$ 892.1050, obs. $[M+2H]^{2+}$ 1338.1601, $[M+3H]^{3+}$ 892.4426.

H–BAGE4$_{18\text{-}32}$–NH$_2$ = H–RLMKEESPVVSWWLE–NH$_2$ was synthesised on Rink Amide resin, 0.167 mmol. The crude peptide was cleaved and 0.023 mmol was purified by preparative RP-HPLC (20 to 60% B over 30 min) and lyophilised to produce the desired peptide (11 mg, 0.006 mmol, 26% yield).

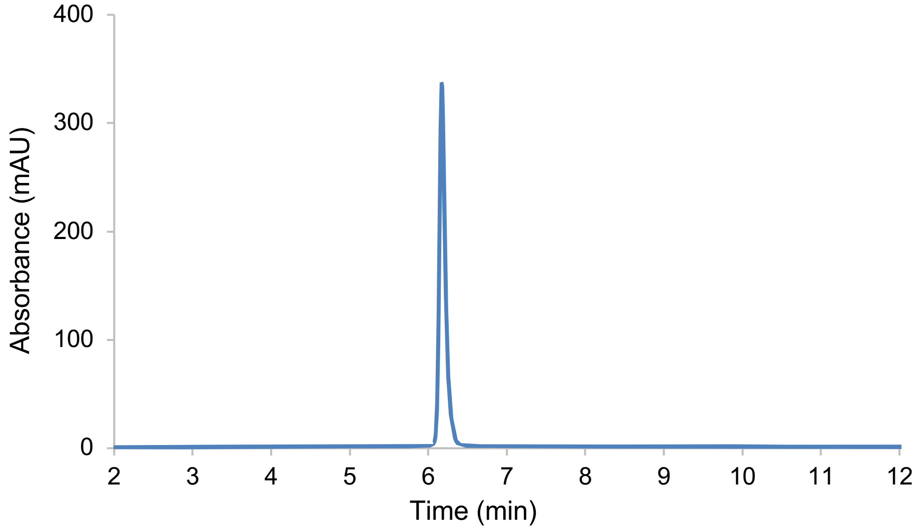

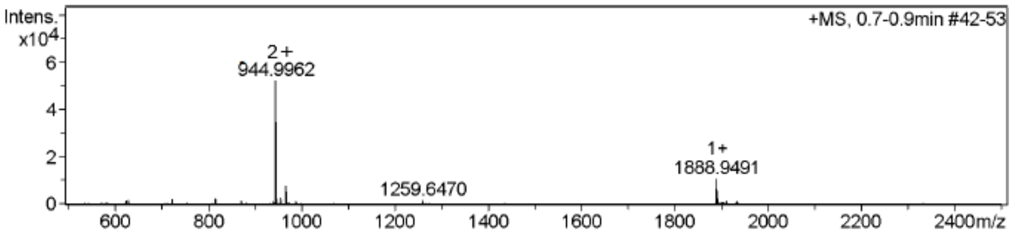

Figure S7. Analytical HPLC trace of pure H–BAGE4$_{18\text{-}32}$–NH$_2$. Analytical gradient 2–95% B over 10 min 0.6 ml/min, 280 nm. ESI$^+$ MS *m/z* calc. $[M+H]^+$ 1887.9893, $[M+2H]^{2+}$ 944.4929, obs. $[M+H]^+$ 1888.9491, $[M+2H]^{2+}$ 944.9962.

H–BAGE4$_{23\text{-}31}$–NH$_2$ = H–ESPVVSWWL–NH$_2$ was synthesised on Rink Amide resin, 0.16 mmol. The crude peptide was cleaved and 0.03 mmol was purified by preparative RP-HPLC (40 to 80% B over 30 min) and lyophilised to produce the desired peptide (7 mg, 0.006 mmol, 20% yield).

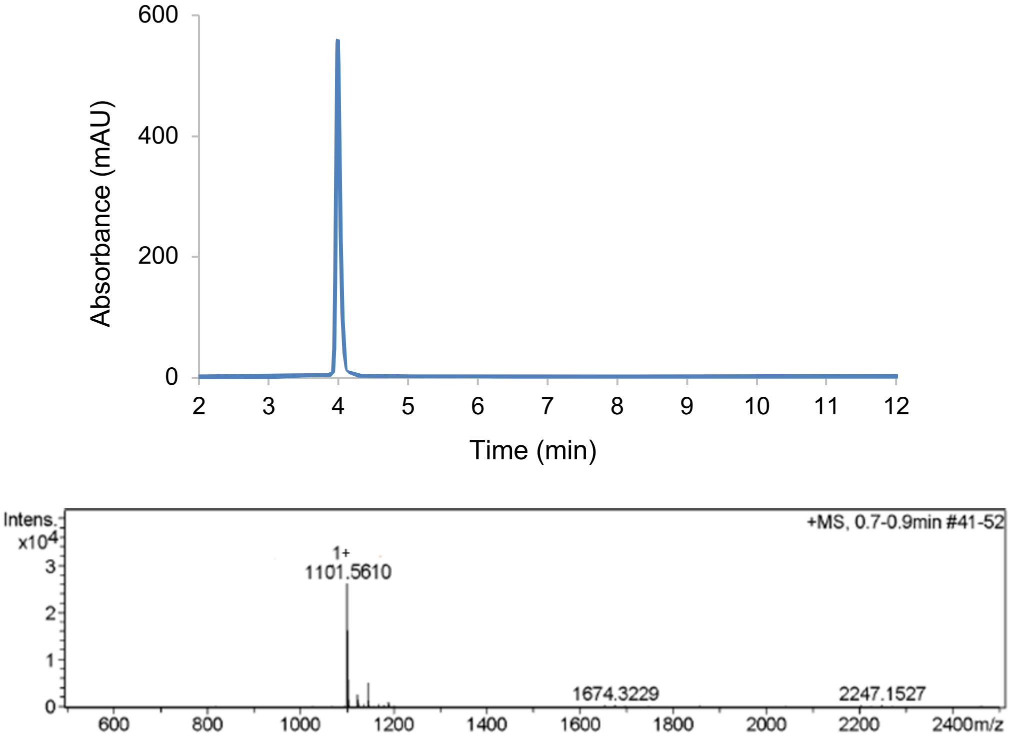

Figure S8. Analytical HPLC trace of pure H–BAGE4$_{23\text{-}31}$–NH$_2$. Analytical gradient 2–60% B over 10 min 0.6 ml/min, 280 nm. HRMS *m/z* calc. $[M+H]^+$ 1101.5655, obs. $[M+H]^+$ 1101.5610.

Pam_2Cys–Antigen Synthesis

General Procedure for Coupling Pam_2Cys to Peptide Antigen (Protocol A)

A solution of Fmoc-diol-Cys-OH (**14**, 1.5 eq.), DIC (1.5 eq.) and Oxyma Pure (1.5 eq.) in DMF (2 mL) was stirred at 0 °C for 5 min, then added to the peptide on resin and agitated for 4 h. The resin was washed with DCM (5 × 3 mL) and DMF (5 × 3 mL). To the resin-bound peptide in DCM (5 mL) was added palmitic acid (20 eq.), DIC (25 eq.) and 4-dimethylaminopyridine (DMAP) (2.0 eq.). The mixture was agitated for 18 h, then washed with DCM (5 × 3 mL). Fmoc group deprotection and resin cleavage were carried out as detailed in the standard Fmoc-SPPS procedures above. The crude product was purified *via* preparative RP-HPLC using a C-4 semi-preparative column (see general methods section for column details).

General Procedure for Coupling Pam_2Cys to Peptide Antigen (Protocol B)

To a solution of Fmoc-Pam_2Cys-OH (**4**, 1.2 eq.) in DMF (5 mL), was added DIC (1.8 eq.) and Oxyma Pure (1.8 eq.). After agitation for 5 min at rt, the resin was added and the mixture agitated at rt for 18 h, then the resin was washed with DCM (5 × 3 mL). Fmoc group deprotection and resin cleavage were carried out as detailed in the standard Fmoc-SPPS procedures above. The crude product was purified *via* preparative RP-HPLC using a C-4 semi-preparative column (see general methods section for column details).

Pam$_2$Cys-BAGE4$_{18\text{-}39}$ (1) was synthesised according to general protocol A using H–BAGE4$_{18\text{-}39}$ on 2-CTC resin (300 mg, 0.018 mmol). The crude peptide was cleaved and purified by semi-preparative RP-HPLC (40 to 80% B over 60 min) and lyophilised to produce the desired Pam$_2$Cys-BAGE4$_{18\text{-}39}$ **1** (7 mg, 0.00217 mmol, 12% yield).

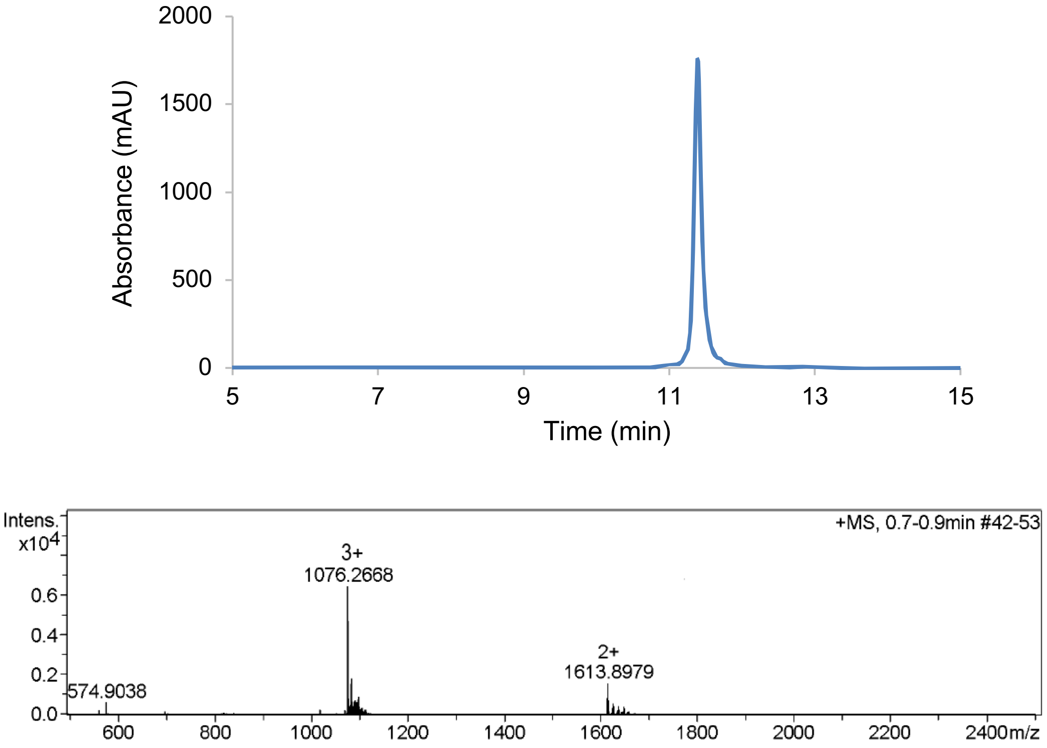

Figure S9. Analytical HPLC trace of pure Pam$_2$Cys-BAGE4$_{18\text{-}39}$ (**1**). Analytical gradient 2–100% B over 10 min, 280 nm. ESI$^+$ MS *m/z* calc. $[M+2H]^{2+}$ 1613.3939, $[M+3H]^{3+}$ 1075.9317, obs. $[M+2H]^{2+}$ 1613.8979, $[M+3H]^{3+}$ 1076.2668.

Pam_2Cys-SK_4-$BAGE4_{18-39}$ (2) was synthesised according to general protocol B using SK_4-$BAGE4_{18-39}$ on Rink Amide resin (210 mg, 0.025 mmol). 0.0125 mmol of the crude peptide was purified by preparative RP-HPLC (40 to 80% B over 60 min) and lyophilised to produce the desired Pam_2Cys-SK_4-BAGE4 **2** (9 mg, 2.8 μmol, 22% yield); yield calculation based on fraction of crude material purified.

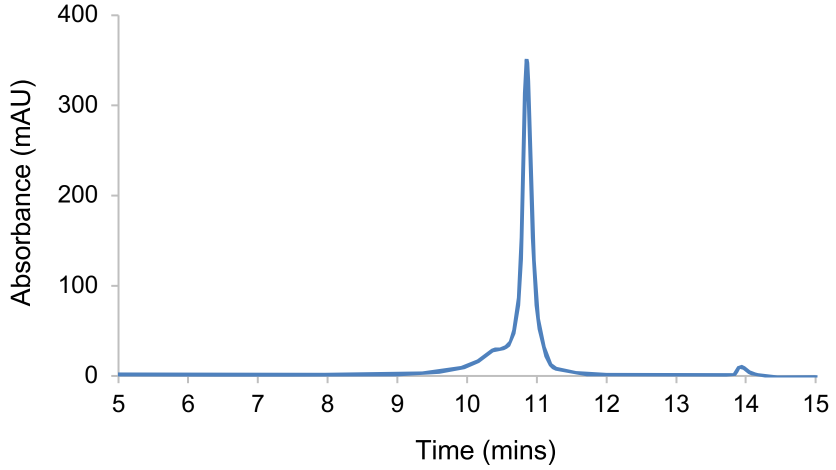

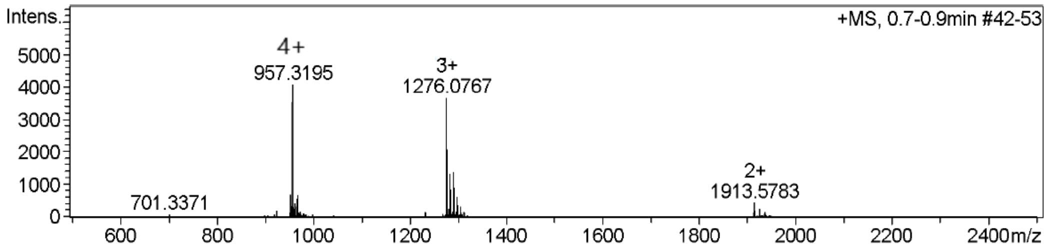

Figure S10. Analytical HPLC trace of pure Pam_2Cys-SK_4-$BAGE4_{18-39}$ (**2**). Analytical gradient 2–100% B over 10 min, 280 nm. ESI^+ MS *m/z* calc. $[M+2H]^{2+}$ 1912.6078, $[M+3H]^{3+}$ 1275.4076, $[M+4H]^{4+}$ 956.8076, obs. $[M+2H]^{2+}$ 1913.5783, $[M+3H]^{3+}$ 1276.0767 $[M+4H]^{4+}$ 957.3195.

Pam$_2$Cys-SK$_4$-NY-ESO-1$_{157-165}$ (3) was synthesised according to general protocol B using SK$_4$-NY-ESO-1$_{157-165}$ on Rink Amide resin (185 mg, 0.03 mmol). For the coupling reagent DIC/Oxyma were replaced with 1.3 eq of each PyBOP and HOBT in present of 2.7 eq of DIPEA. 0.015 mmol of the crude cleaved peptide was purified by semi-preparative RP-HPLC (40 to 80% B over 60 min) and lyophilised to produce the desired Pam$_2$Cys-SK$_4$-NY-ESO-1$_{157-165}$ **3** (9.4 mg, 4 μmol, 26 % yield); yield calculation based on fraction of crude material purified.

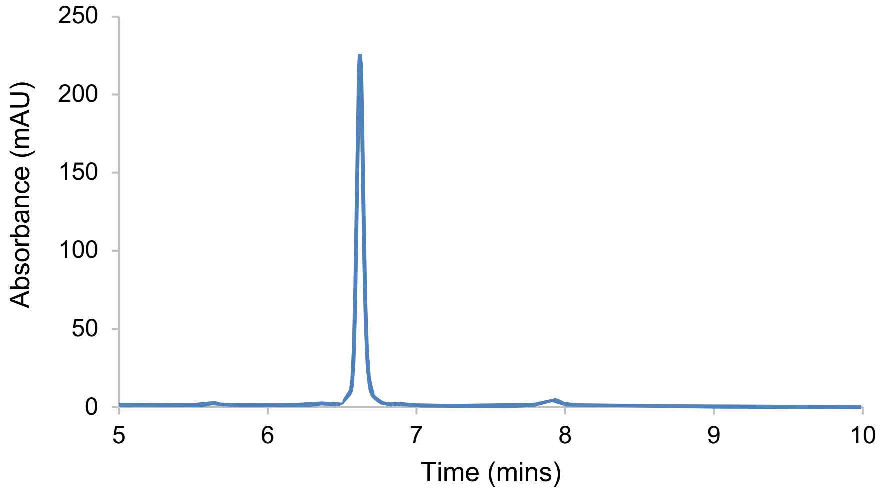

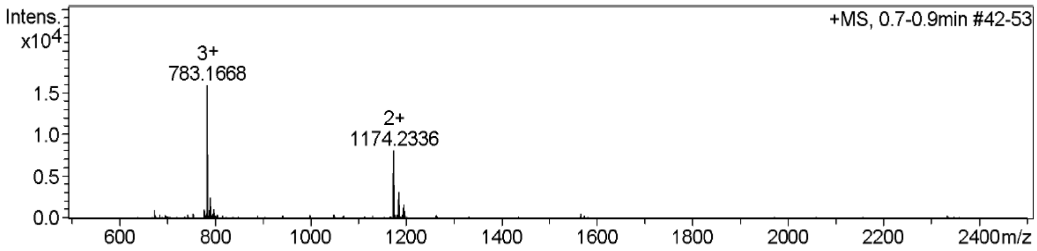

Figure S11. Analytical HPLC trace of pure Pam$_2$Cys-SK$_4$-NY-ESO-1$_{157-165}$ (**3**). Analytical gradient 10–100% B over 5 min, 280 nm. ESI$^+$ MS *m/z* $[M+2H]^{2+}$ 1173.7389, $[M+3H]^{3+}$ 782.8283, obs.$[M+2H]^{2+}$ 1174.2336, $[M+3H]^{3+}$ 783.1668.

Structural Evaluation of Peptides

Circular Dichroism

Both peptides exhibited CD spectra characteristic of an α-helical structure, however a much higher α-helix content was observed for Pam$_2$Cys-SK$_4$-NY-ESO-1$_{157-165}$ **3** compared to Pam$_2$Cys-SK$_4$-BAGE$_{18-39}$ **2**.

Pam$_2$Cys-SK$_4$-BAGE4 (**2**): f_α = 0.066

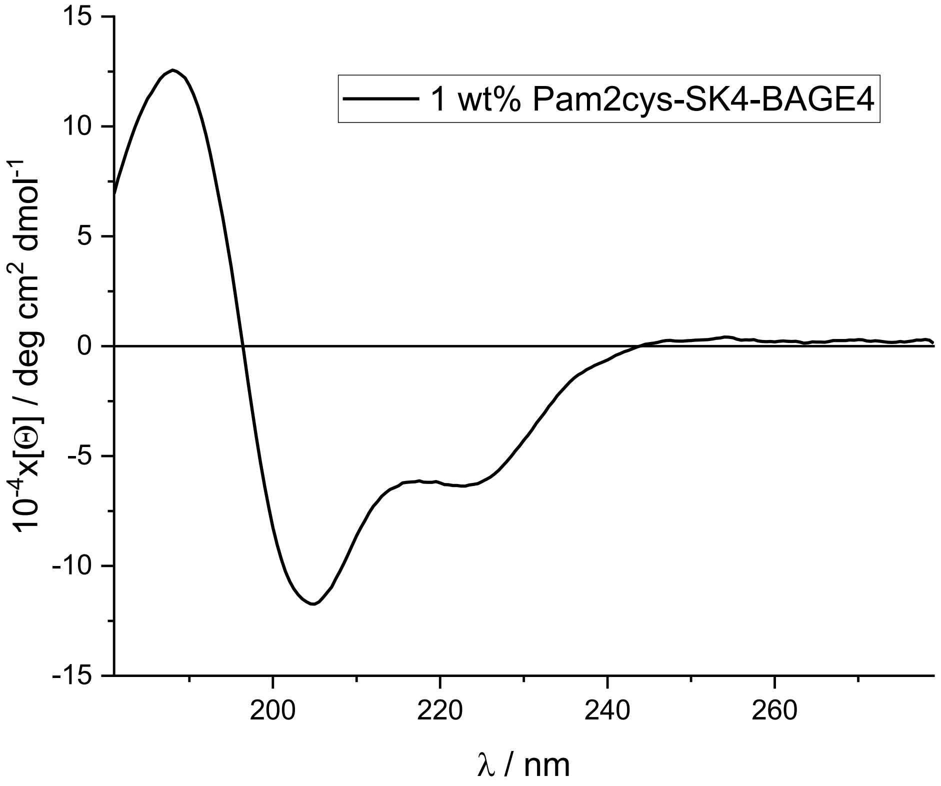

Figure S12. CD spectrum recorded for Pam$_2$Cys-SK$_4$-BAGE4$_{18-39}$ (**2**)

Pam_2Cys-SK_4-NY-ESO-1 (**3**): $f_\alpha = 0.407$

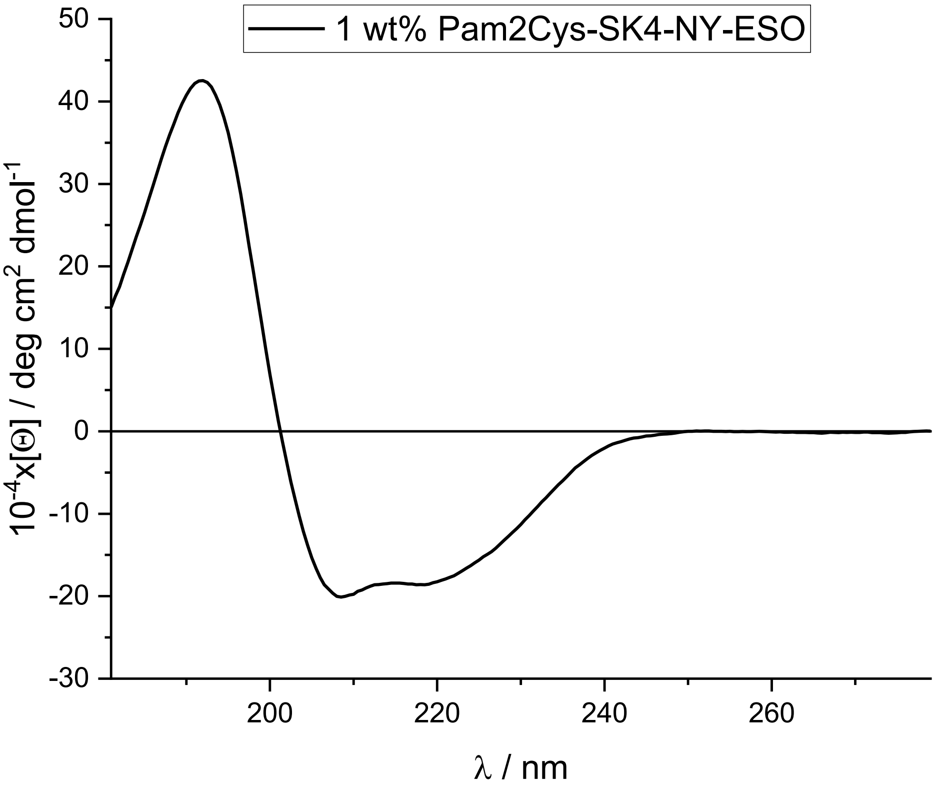

Figure S13. CD spectrum recorded for Pam_2Cys-SK_4-NY-ESO-$1_{157\text{-}165}$ (**3**)

The observed CD data was consistent with predicted data from PEPFOLD modelling:

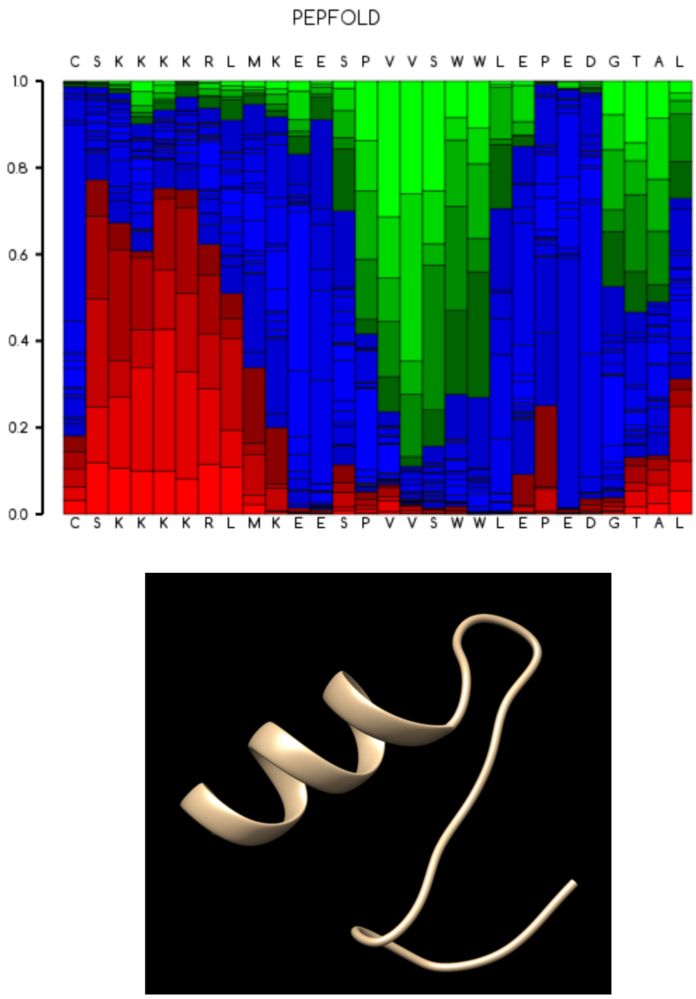

Figure S14. Modelling data generated by PEPFOLD for SK$_4$-BAGE4$_{18-39}$, showing disordered C-terminal domain. Red: α-helix. Blue: disordered. Green: 'extended'.

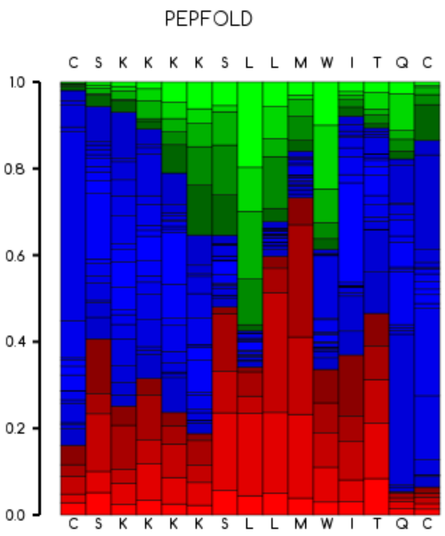

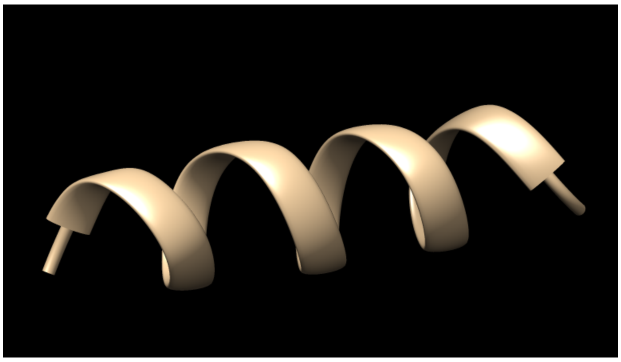

Figure S15. Modelling data generated by PEPFOLD for SK$_4$-NY-ESO-1$_{157\text{-}165}$. Red: α-helix. Blue: disordered. Green: 'extended'.

Cryo-TEM – fibril formation observed

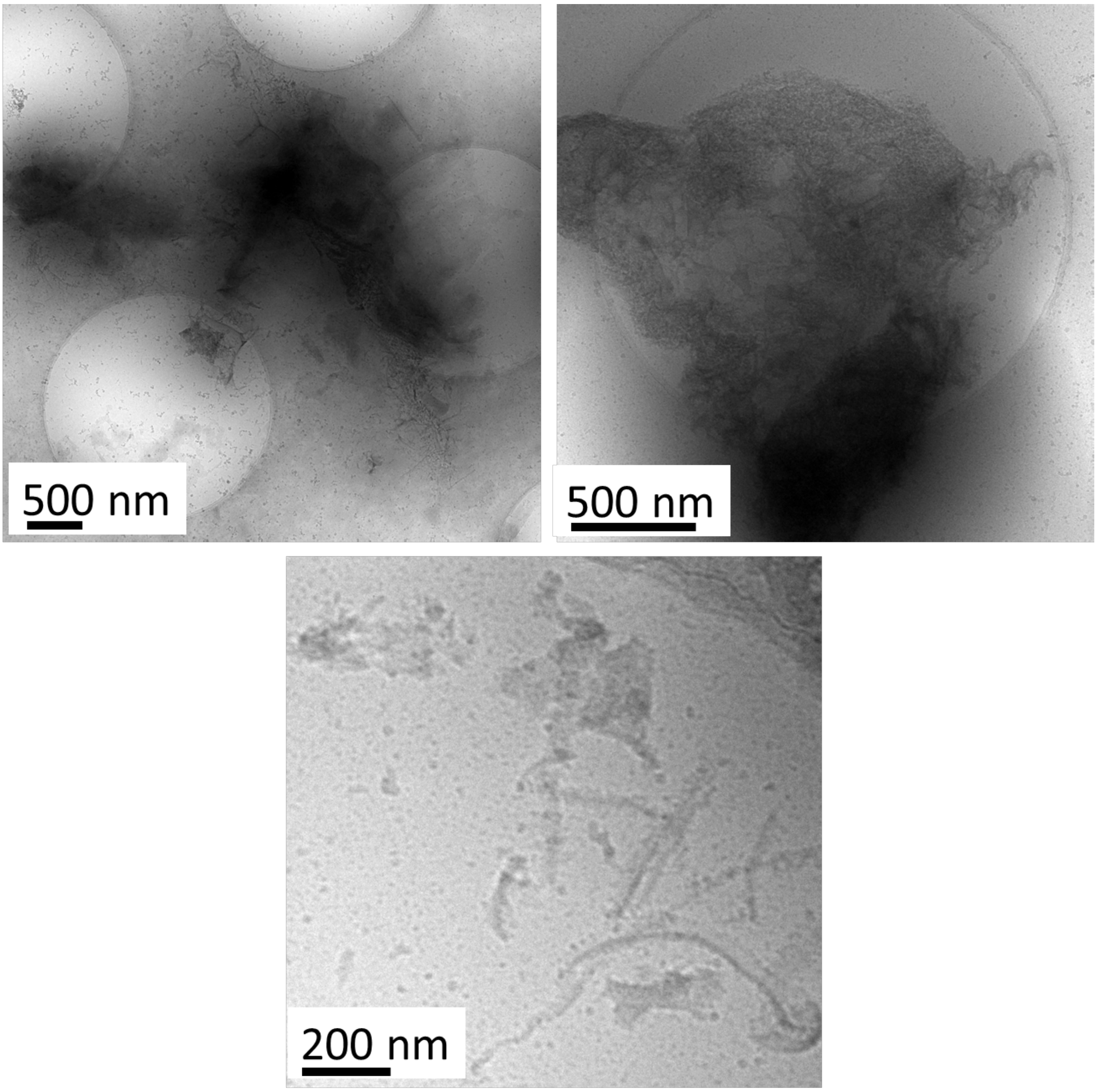

Figure S16. Cryo-TEM images for 1wt% aqueous solution of Pam_2Cys-SK_4-$BAGE4_{18\text{-}39}$ (**2**).

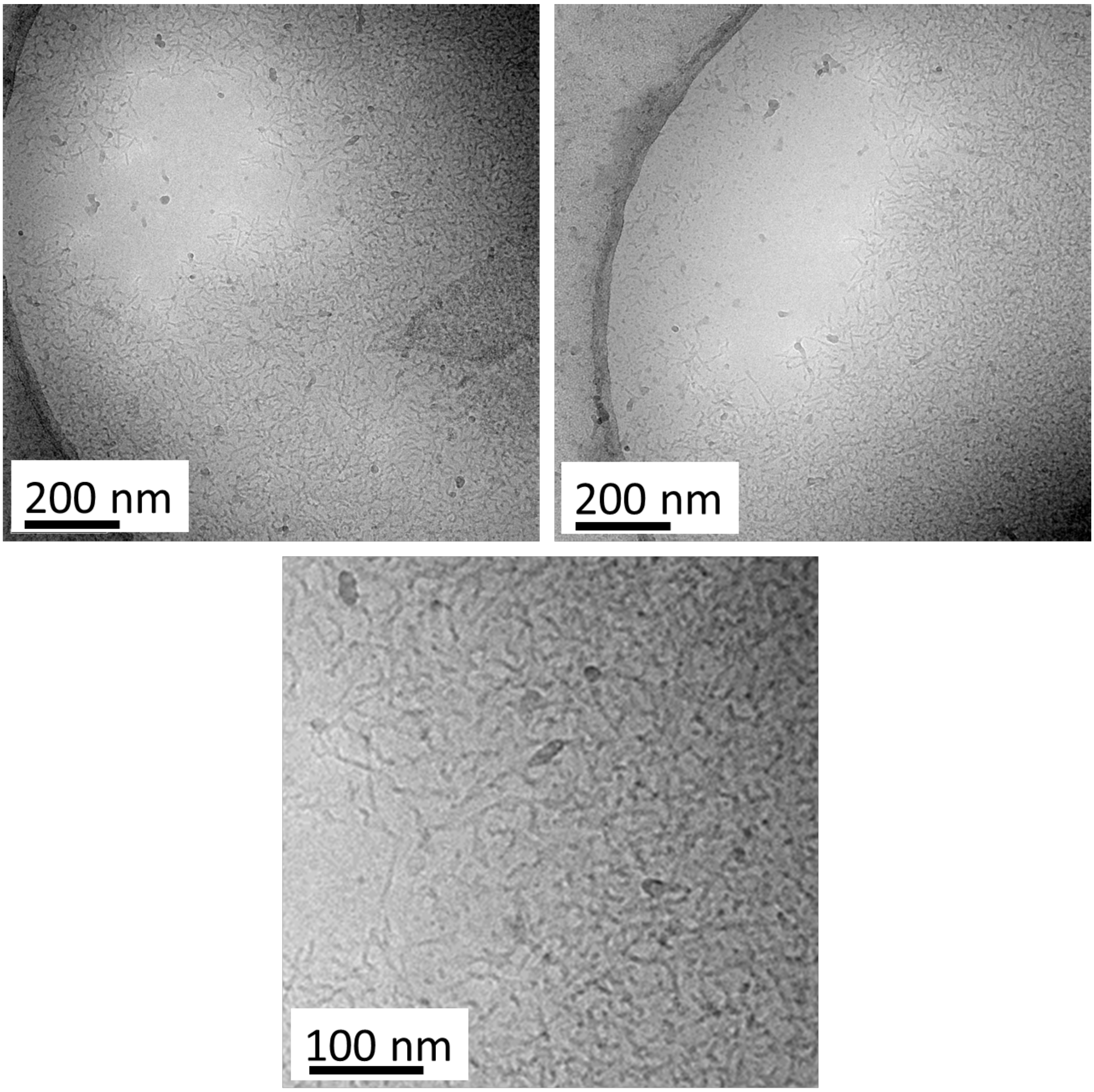

Figure S17. Cryo-TEM images for 1wt% aqueous solution of Pam_2Cys-SK_4-NY-ESO-$1_{157\text{-}165}$ (**3**).

SAXS models. The SAXS intensity from a scattering object without a particular orientation can be approximated by the following equation for monodisperse particles: [Pedersen, J. S.; Svaneborg, C., Scattering from block copolymer micelles. *Curr. Opin. Coll. Int. Sci*. 2002, **7**, 158-166. 2. Castelletto, V.; Hamley, I. W., Modelling Small-Angle Scattering Data from Micelles. *Curr. Opin. Coll. Int. Sci.* 2002, **7**, 167-172.]:

$$I(q) \propto < F^2(q) > S(q) \quad (1)$$

Where $<F^2(q)>$ is the average form factor (due to the particle size and shape) and $S(q)$ is the interparticle interference function (structure factor). For dilute systems, such as those studied in this work, $S(q) \sim 1$. We used the form factor for a core-shell cylinder.[3] The fitting parameters for the cylindrical shell form factor were the core radius, R*,* the shell thickness, D_r, and the scattering length density of the core, η_{core}, shell, η_{shell}, and solvent, η_{solv}. All fitting was done using software SASfit.[4]

Small Angle X-Ray Scattering (SAXS)

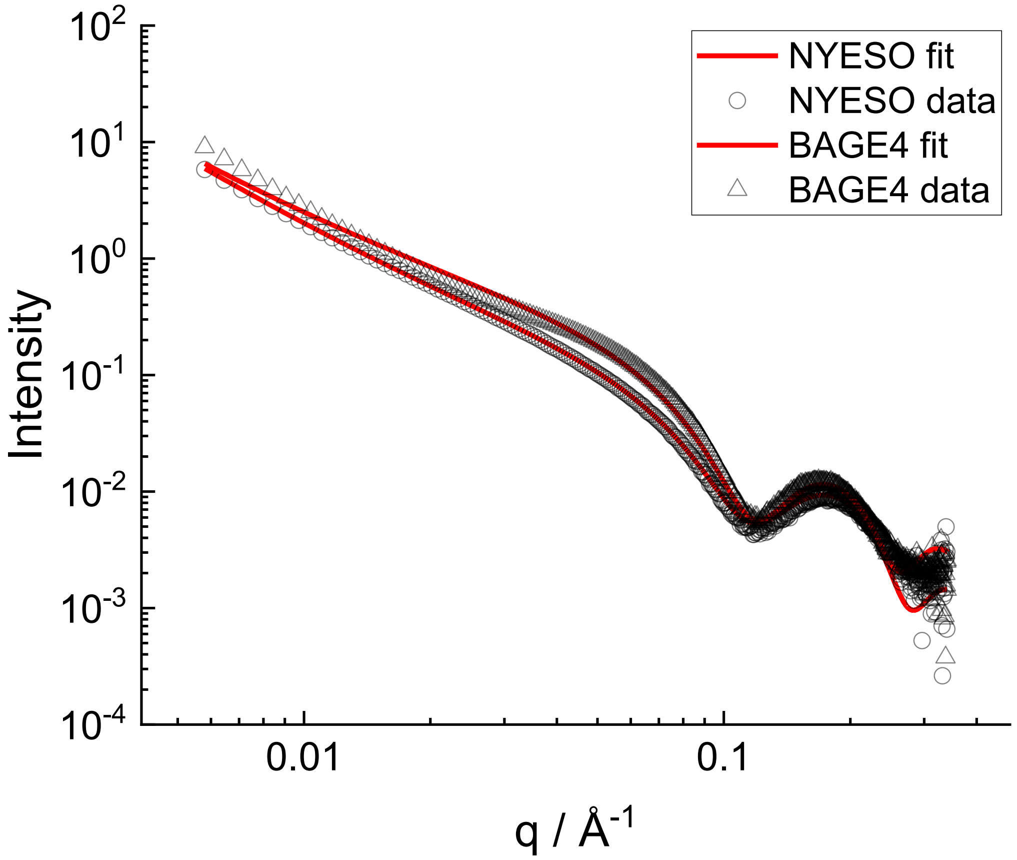

Figure S18. Small Angle X-Ray Scattering (SAXS) data for 1 wt% solutions of peptides $Pam_2Cys-SK_4-BAGE4_{18-39}$ (**2**) and $Pam_2Cys-SK_4$-NY-ESO-1 (**3**). Data fitted with core-shell cylinder form factor and indicates the formation of fibrils. The SAXS fitting parameters are listed in Table S1.

Table S1. SAXS fit parameters using a core-shell cylinder form factor.

	1 wt% Pam_2Cys-SK_4-$BAGE4_{18-39}$ (**2**)	1 wt% Pam_2Cys-SK_4-NY-ESO-$1_{157-165}$ (**3**)
R ± ΔR [Å]	18.5±1.5	14.0±0.2
D_r [Å]	4.8	10.9
L [Å]	16000	2364
η_{core} [cm^{-1}]	9.3×10^{-8}	-7.9×10^{-7}
η_{shell} [cm^{-1}]	2.4×10^{-6}	2.7×10^{-6}
η_{solv} [cm^{-1}][a]	1.0×10^{-8}	1.0×10^{-8}
I_0 [cm^{-1}]	8.0×10^{-4}	2.9×10^{-4}
I_1 [cm^{-1}]	3.1×10^{-5}	3.6×10^{-4}
n	2.3	2.3

Key: core radius, R (Gaussian polydispersity DR); shell thickness, D_r; cylinder length, L; scattering length density of the core, h_{core}, shell, h_{shell}, and solvent, $h_{solv.}$; sloping background $I_0 + I_1q^{-n}$. Note that since L >> R, L just represents a scaling factor in the fit. [a] Fixed parameter.

Synthesis of Organic Compounds

(*R*)-4-(Iodomethyl)-2,2-dimethyl-1,3-dioxolane (7)

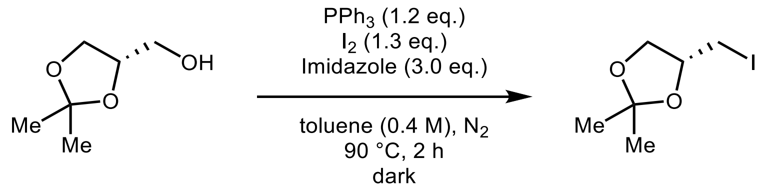

To a solution of (*S*)-solketal (**6**, 1.98 g, 15.0 mmol, 1.00 eq.) in toluene (37.5 mL, 0.4 M) was added triphenylphosphine (4.72 g, 18.0 mmol, 1.20 eq.), imidazole (3.06 g, 45.0 mmol, 3.0 eq.), and iodine (4.60 g, 19.5 mmol, 1.30 eq.). The reaction mixture was stirred at 90 ºC under N_2, in the dark. After 2 hours, the solvent was removed under reduced pressure, and the resulting residue was dissolved in DCM (100 mL). The solution was washed with 12% aq. $Na_2S_2O_3$ (3 × 50 mL) and brine (50 mL), then dried ($MgSO_4$), filtered, and concentrated *in vacuo*. Triphenylphosphine oxide was removed from the residue by precipitation into diethyl ether, and the supernatant was concentrated *in vacuo*. Excess triphenylphosphine was removed from the residue by precipitation into ice cold pentane, and the supernatant was concentrated *in vacuo* to afford the title compound as a colourless oil (**7**, 2.90 g, 11.8 mmol, 79%), which was used in the next step without further purification. The analytical data was consistent with those reported in the literature.[5]

(*R*)-3-Iodopropane-1,2-diol (8)

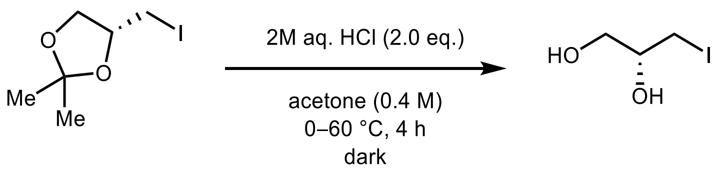

To a solution of (*R*)-4-(iodomethyl)-2,2-dimethyl-1,3-dioxolane (**7**, 2.42 g, 10.0 mmol, 1.0 eq.) in acetone at 0°C (25 mL, 0.4 M) was added HCl (2 M in H_2O, 10.0 mL, 20.0 mmol, 2.0 eq.). The reaction mixture was heated to 60 °C and stirred for 4 h in the dark. After completion of the reaction, sat. aq. $NaHCO_3$ (25 mL) was added, and the product was extracted into EtOAc (3 × 50 mL). The combined organic extracts were dried ($MgSO_4$), filtered, and concentrated *in vacuo* to afford the title compound as a colourless solid (**8**, 1.72 g, 8.52 mmol, 85%), which was used in the next step without further purification. The analytical data was consistent with those reported in the literature.[6]

(*R*)-3-Iodopropane-1,2-diyl dipalmitate (9)

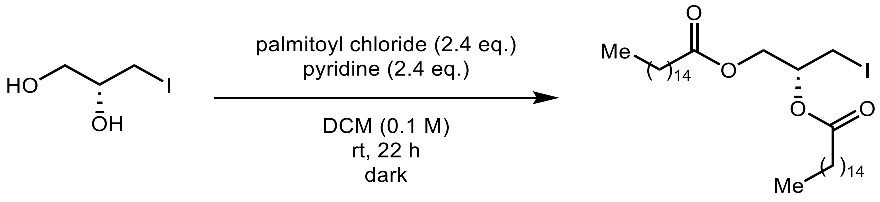

To a solution of (*R*)-3-Iodopropane-1,2-diol (**8**, 1.72 g, 8.52 mmol, 1.0 eq.) in DCM (100 mL, 0.09 M) was added pyridine (1.62 mL, 20.0 mmol, 2.35 eq.). To the reaction mixture was added palmitoyl chloride (6.07 mL, 20.0 mmol, 2.35 eq.), dropwise over 20 min. The reaction was stirred at room temperature for 12 h, in the dark. The solvent was removed *in vacuo*, and the resulting residue was dissolved in EtOAc (100 mL). H_2O (100 mL) was added, and the organic phase separated. The aqueous phase was extracted with EtOAc (2 × 100 mL), then the combined organic phases were dried ($MgSO_4$), filtered, and concentrated *in vacuo*. Recrystallisation from methanol afforded the title compound (**9**, 3.98 g, 4.99 mmol, 59%). The analytical data was consistent with those reported in the literature.[7]

^{1}H NMR (400 MHz, $CDCl_3$) δ_H 5.00 (dddd, *J* = 5.8, 5.7, 5.7, 4.2 Hz, 1H), 4.31 (dd, *J* = 11.9, 4.2 Hz, 1H), 4.22 (dd, *J* = 11.9, 5.7 Hz, 1H), 3.35 (dd, *J* = 10.7, 5.8 Hz, 1H), 3.28 (dd, *J* = 10.7, 5.7 Hz), 2.36–2.28 (m, 4H), 1.67–1.58 (m, 4H), 1.32–1.25 (m, 52H), 0.88 (t, *J* = 7.0 Hz, 6H).

^{13}C NMR (101 MHz, $CDCl_3$) δ_C 173.4 (C), 172.9 (C), 70.1 (CH_2), 64.3 (CH_2), 34.4 (CH), 34.2 (CH_2), 32.1 (CH_2), 29.9–29.9 (m, CH_2), 29.8 (CH_2), 29.6 (CH_2), 29.4 (CH_2), 29.3 (CH_2), 25.1 (CH_3), 25.0 (CH_3), 22.8 (CH_2).

(*R*)-(2,3-dihydroxypropyl)cysteine (13)

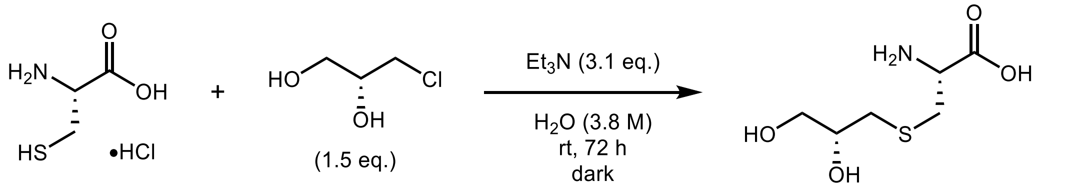

A mixture of L-cysteine hydrochloride (**11**, 3.34 g, 19.0 mmol), (*R*)-3-chloro-1,2-propanediol (**12**, 2.40 mL, 28.5 mmol, 1.5 eq.) and triethylamine (8.08 mL, 58 mmol, 3.1 eq.) in water (5 mL) was stirred in the dark at rt. After 72 h the reaction mixture was concentrated *in vacuo* and the residue was washed with acetone (3 × 5 mL) and dried to give the title compound as an off white solid (**13**, 3.5 g, 18.2 mmol, 95%), which was used without further purification.

HRMS *m/z* calc. for $C_6H_{13}NO_4S$ $[M+H]^+$ 196.0643, obs. $[M+H]^+$ 196.0638 (2.55 ppm error).

^{1}H NMR (400 MHz, DMSO-d_6) δ_H 7.89 (d, J = 7.4 Hz, 2H), 7.74 (d, J = 7.5 Hz, 2H), 7.68–7.66 (m, 1H), 7.42 (t, J = 7.6 Hz, 2H), 7.33 (t, J = 7.5 Hz, 2H), 4.30–4.22 (m, 3H), 4.14 (td, J = 9.2, 4.6 Hz, 1H), 3.58 (p, J = 5.60 Hz, 1H), 2.98 (dd, J = 13.6, 4.6 Hz, 1H), 2.80 (dd, J = 13.6, 9.5 Hz, 1H), 2.70 (dd, J = 13.4, 4.7 Hz, 1H), 3.40–3.31 (m, 4H).

Fmoc-(*R*)-(2,3-dihydroxypropyl)cysteine (14)

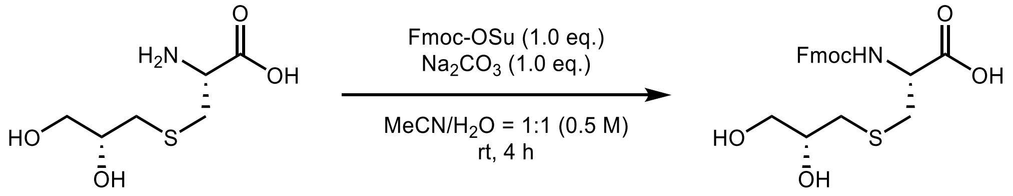

To a solution of fluorenylmethoxycarbonyl-*N*-hydroxysuccinimide (Fmoc-OSu) (6.10 g, 18.2 mmol, 1.0 eq.) in acetonitrile (20 mL) was added to a solution of (*R*)-(2,3-dihydroxypropyl)cysteine (**13**, 3.50 g, 18.2 mmol, 1.0 eq.) and sodium carbonate (1.93 g, 18.2 mmol, 1.0 eq.) in water (20 mL). The reaction mixture was stirred at room temperature for 4 h, then water (50 mL) was added, and the solution was acidified to pH 2 with concentrated hydrochloric acid. The reaction mixture was extracted with EtOAc (3 × 50 mL), and the combined organic extracted were washed with water (2 × 50 mL) and brine (2 × 50 mL). The organic extracts were dried ($MgSO_4$), filtered and concentrated *in vacuo* to afford the title compound as a sticky off white solid. Recrystallization from Et_2O–EtOAc at –20 °C yielded a white powder (**14**, 870 mg, 3.60 mmol, 20%).

HRMS *m/z* calc. for $C_{21}H_{23}NO_6S$ $[M–H]^-$ 416.1168, $[2M-H]^-$ 833.2492, obs. $[M-H]^-$ 416.1181, $[2M–H]^-$ 833.2392 (3.12 ppm error).

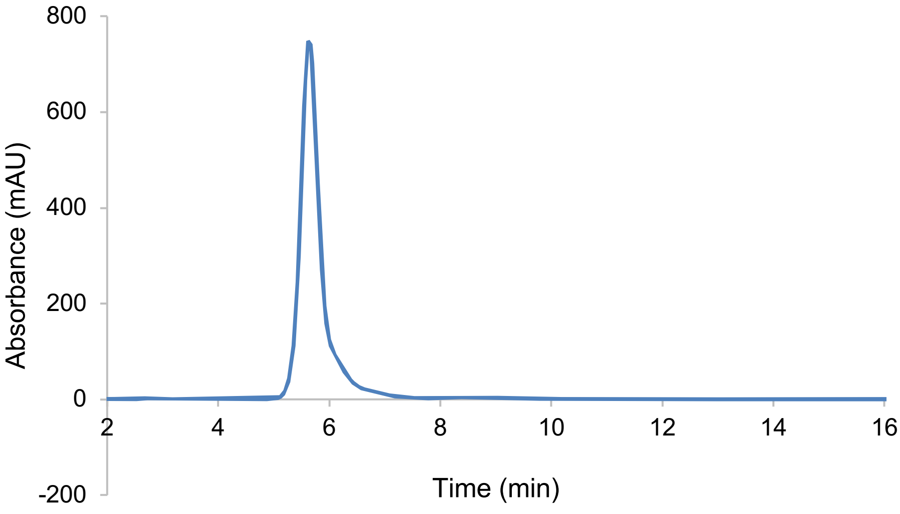

Figure S19. Analytical HPLC trace of Fmoc-(*R*)-(2,3-dihydroxypropyl)cysteine (**14**). Analytical gradient 30% isocratic for 14 min; 0.6 mL/min, 280 nm.

Fmoc-(*R*)-(2,3-dihydroxypropyl)cysteine *tert*-butyl ester (17)[8]

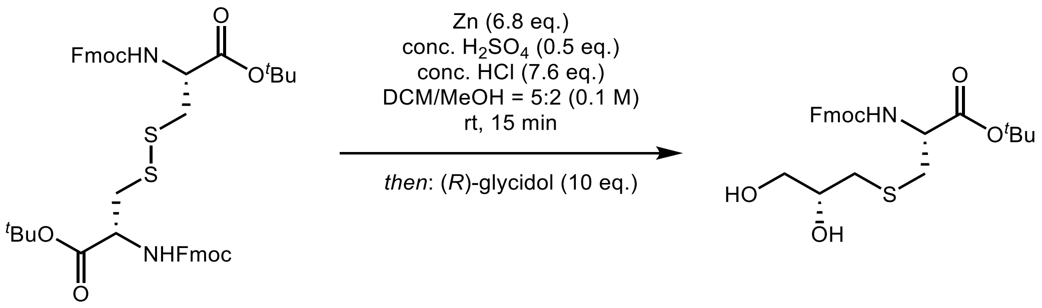

To a solution of (Fmoc-Cys-OtBu)$_2$ (**16**, 1.25 mmol, 996 mg) in DCM (10 mL) was added zinc dust (8.75 mmol, 572 mg, 6.8 eq.) and H_2SO_4/HCl/MeOH (4 mL, 1/7/100). The reaction mixture was stirred at rt for 15 min, then (*R*)-glycidol (12.5 mmol, 0.841 mL, 10 eq.) was added and the mixture was stirred at 40 °C for 5 h. The reaction mixture was filtered and then concentrated *in vacuo* to a volume of ca. 5 mL. The resulting residue was diluted with EtOAc (10 mL) and washed with 2M HCl (10 mL). The aqueous layer was back extracted with EtOAc (3 × 20 mL),. The combined organic layers were dried ($MgSO_4$), filtered and concentrated *in vacuo* to afford the crude product. Purification of the crude material by silica gel column chromatography (50–70% EtOAc/Pentane) afforded the product as a white solid (**17**, 0.84 mmol, 397 mg, 68%). The analytical data was consistent with those reported in the literature.[8]

HRMS *m/z* calc. for $C_{25}H_{31}NO_6S$ $[M+H]^+$ 474.1950, obs. $[M+H]^+$ 474.1933

^{1}**H NMR** (400 MHz, $CDCl_3$) δ 7.76 (d, *J* = 7.5 Hz, 2H, Ar*H*), 7.61 (d, *J* = 7.5 Hz, 2H, Ar*H*), 7.40 (t, *J* = 7.4 Hz, 2H, Ar*H*), 7.31 (t, *J* = 7.4 Hz, 2H, Ar*H*), 5.85 (d, *J* = 8.0 Hz, 1H, -N*H*), 4.54–4.48 (m, 1H, C*H*-N), 4.40 (d, *J* = 7.0 Hz, 2H, Fmoc-CH_2-O), 4.23 (t, *J* = 7.0 Hz, 1H, Fmoc-C*H*-), 3.95–3.86 (m, impurity), 3.82–3.78 (m, 1H, -O*H*), 3.75 (dd, *J* = 11.3, 3.9 Hz, 1H, CH_aH$_b$-O), 3.71–3.65 (m, 1H, -C*H*-OH), 3.64–3.55 (m, impurity), 3.52 (dd, *J* = 11.3, 6.0 Hz, 1H, CH$_a$$H_b$-O), 3.03–2.90 (m, 2H, C(N)-CH_aH$_b$-S, C(N)-CH$_a$$H_b$-S), 2.83–2.74 (m, 1H, C(O)-CH_aH$_b$-S), 2.68–2.57 (m, overlap, 1H, C(O)-CH$_a$$H_b$-S), 1.49 (s, 9H, -C$H_3$).

^{13}**C NMR** (101 MHz, $CDCl_3$) δ 171.1 (C), 157.5 (C), 145.2 (C), 145.1 (C), 142.7 (C), 129.2 (CH), 128.5 (CH), 126.5 (CH), 121.4 (CH), 84.6 (C), 72.0 (CH), 68.6 (CH_2), 66.6 (CH_2), 55.9 (CH), 47.4 (CH), 38.1 (CH_2), 37.1 (CH_2), 29.4 (CH_3).

Fmoc-Pam$_2$Cys *tert*-butyl ester (4-O^{t}Bu)[9]

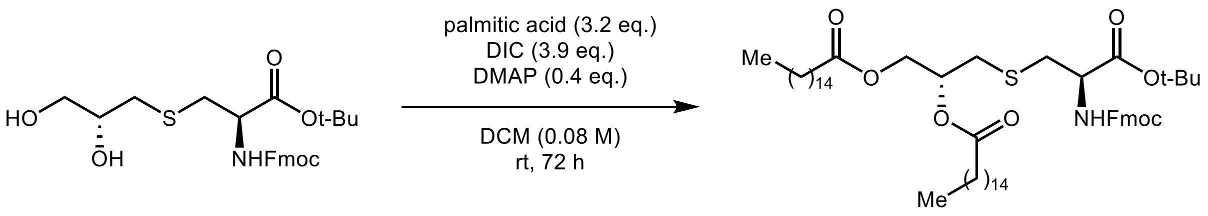

Compound **17** (11.5 mmol, 5.44 g) was dissolved in dry DCM (150 mL) under argon. Consecutively, palmitic acid (36.8 mmol, 9.42 g, 3.2 eq.), DIC (44.9 mmol, 5.65 g, 3.9 eq.) and DMAP (4.60 mmol, 562 mg, 0.4 eq.) were added. The reaction mixture was stirred at rt for 72 h, then glacial acetic acid (86.3 mmol, 5 mL) was added. The solution was stirred at rt for 15 min, then filtered and concentrated *in vacuo*. Crystallization in DCM/MeOH (1:19) yielded compound **4-O^{t}Bu** (8.9 mmol, 8.46 g, 77%).

HRMS *m/z* calc. for $C_{57}H_{91}NO_8S$ $[M+Na]^+$ 972.6363, obs. $[M+Na]^+$ 972.6389.

^{1}**H NMR** (400 MHz, $CDCl_3$) δ 7.79 (d, *J* = 7.5 Hz, 2H, Ar*H*), 7.64 (d, *J* = 7.5 Hz, 2H, Ar*H*), 7.42 (t, *J* = 7.4 Hz, 2H, Ar*H*), 7.34 (t, *J* = 7.4 Hz, 2H, Ar*H*), 5.73 (d, *J* = 7.6 Hz, 1H, -N*H*), 5.20–5.14 (m, 1H, C*H*-OH), 4.55–4.51 (m, 1H, C*H*-N), 4.43 (m, 1H, CH_aH$_b$-O), 4.38–4.32 (m, 2H, Fmoc-CH_2-O), 4.26 (t, *J* = 7.1 Hz, 1H, Fmoc-C*H*-), 4.20–4.13 (m, 1H, CH$_a$$H_b$-O), 3.88–3.78 (m, impurity), 3.72–3.61 (m, impurity), 3.5 (s, impurity, Methanol), 3.13–3.00 (m, 2H, C(N)-CH_2-S), 2.82–2.72 (m, 2H, C(O)-CH_2-S), 2.39–2.24 (m, 4H, -CH_2-COO with PA as impurity), 1.68–1.55 (m, 4H, -CH_2-CH$_2$-COO with PA as impurity), 1.54–1.45 (m, 9H, -CH_3 x 3), 1.37–1.20 (m, 48H, -(CH_2-)$_{12}$ with PA as impurity), 0.90 (t, *J* = 6.7 Hz, 6H, -CH_3 with PA as impurity). The

spectrum of the analysed sample is a mixture of **4-O^{t}Bu** with the starting compound PA (palmitic acid) which affects the integral values of the peaks at 2.3, 1.6, 1.3 and 0.88 ppm. The analytical data was consistent with those reported in the literature.[9]

^{13}C NMR (101 MHz, $CDCl_3$) δ 173.4 (C), 173.3 (C), 169.5 (C), 155.7 (C), 143.8 (C), 141.3 (C), 127.7 (CH), 127.1 (CH), 125.1 (CH), 120.0 (CH), 83.0 (C),70.2 (CH), 67.2 (CH_2), 63.5 (CH_2), 50.9 (CH), 47.1 (CH), 34.3 (CH_2), 34.1 (CH_2), 33.3 (CH_2), 31.9 (CH_2), 29.7 (CH_2), 29.6 (CH_2), 29.5 (CH_2), 29.4 (CH_3), 29.3 (CH_2), 29.2 (CH_2), 29.1 (CH_2), 28.0 (CH_2), 24.9 (CH_2), 24.8 (CH_2), 22.7 (CH_2), 14.0 (CH_3).

Fmoc-Pam$_2$Cys-OH (4)[8]

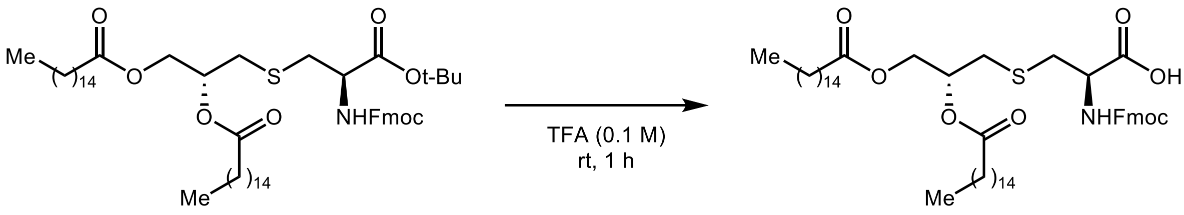

TFA (130.7 mmol, 14.9 g, 9.1 mL) was added to compound **4** (1.2 mmol, 1.1 g) and the solution was stirred at rt for 1 h. Co-evaporation of TFA with toluene (3 × 10 mL) resulted in a crude compound. Purification of the crude compound by flash column chromatography (SiO_2, 15–20% EtOAc/PE with 1% AcOH) yielded the title compound (**4**, 1.03 mmol, 920 mg, 86%). The analytical data was consistent with those reported in the literature.[8]

HRMS *m/z* calc. for $C_{53}H_{83}NO_8S$ $[M+Na]^+$ 916.5737, obs. $[M+Na]^+$ 916.5729 (0.87 ppm error).

^{1}H NMR (400 MHz, $CDCl_3$) δ 7.76 (d, *J* = 7.5 Hz, 2H, Ar*H*), 7.61 (d, *J* = 7.5 Hz, 2H, Ar*H*), 7.40 (t, *J* = 7.4 Hz, 2H, Ar*H*), 7.31 (t, *J* = 7.4 Hz, 2H, Ar*H*), 5.78 (d, *J* = 7.8 Hz, 1H, -N*H*), 5.18–5.16 (m, 1H, C*H*-OH), 4.68–4.63 (m, 1H, C*H*-N), 4.41–4.33 (m, 3H, Fmoc-CH_2-O, CH_aH$_b$-O), 4.24 (t, *J* = 7.0 Hz, 1H, Fmoc-C*H*-), 4.18–4.11 (m, 1H, CH$_a$$H_b$-O), 3.19–3.04 (m, 2H, C(N)-CH_2-S), 2.82–2.73 (m, 2H, C(O)-CH_2-S), 2.31 (app. q, *J* = 7.7 Hz, 4H, -CH_2-COO), 1.64–1.56 (m, 4H, -CH_2-CH_2-COO), 1.32–1.23 (m, 48H, -(CH_2-)$_{12}$), 0.88 (t, *J* = 6.7 Hz, 6H, -CH_3).

^{13}C NMR (101 MHz, $CDCl_3$) δ 173.7 (C), 173.7 (C), 173.6 (C), 155.7 (C), 143.8 (C), 141.4 (C), 127.9 (CH), 127.3 (CH), 125.3 (CH), 120.1 (CH), 70.4 (CH), 67.6 (CH_2), 63.6 (CH_2), 53.8 (CH), 47.2 (CH), 34.8 (CH_2), 34.5 (CH_2), 34.3 (CH_2), 33.1 (CH_2), 32.1 (CH_2), 29.9 (CH_2), 29.8 (CH_2), 29.8 (CH_2), 29.7 (CH_2), 29.5 (CH_2), 29.4 (CH_2), 29.3 (CH_2), 29.3 (CH_2), 25.1 (CH_2), 25.0 (CH_2), 22.8 (CH_2), 14.3 (CH_3).

(S)-3-(Benzylideneamino)propane-1,2-diol (19)[10]

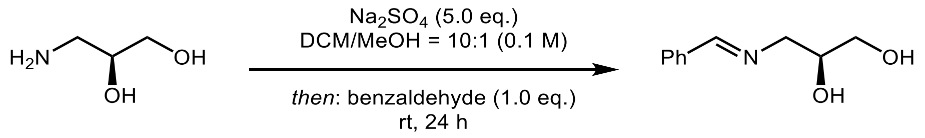

A solution of (*S*)-3-amino-1,2-propanediol (**18**, 200 mg, 2.20 mmol) in anhydrous DCM/MeOH (10:1, 25 ml) was stirred over Na_2SO_4 (1.56 g, 11.0 mmol, 5.0 eq.) for 2 h, then benzaldehyde (270 mg, 2.20 mmol, 1.0 eq.) was added dropwise. After stirring at rt for 24 h, the mixture was filtered, and the filtrate concentrated *in vacuo* to afford the title compound (**19**, 0.28g, 1.56 mmol, 71%), which was used without further purification.

HRMS *m/z* calc. for $C_{10}H_{13}NO_2$ $[M+H]^+$ 180.1024, obs. $[M+H]^+$ 180.1033 (5.00 ppm error).

Cetyl alcohol mesylate (21)[10]

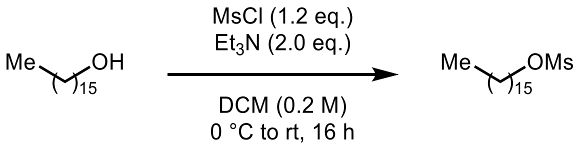

A solution of cetyl alcohol (**20**, 2.5 g, 10.3 mmol) and Et_3N (2.08 g, 20.6 mmol, 2.0 eq.) in DCM (60 mL) was cooled to 0 °C before mesyl chloride (1.42 g, 12.4 mmol, 1.2 eq.) was added dropwise. The solution was allowed to warm to rt and stirred for 16 h before being diluted with DCM (100 mL) and extracted with 1M aq. $NaHCO_3$ (3 × 50 mL). The aqueous layer was extracted with DCM (3 × 50 mL), then the combined organic extracts washed with water (3 × 50 mL), brine (50 mL), dried ($MgSO_4$), filtered and concentrated *in vacuo* to afford the title compound a pale yellow solid (**21**, 3.17 g, 9.89 mmol, 96%), which was used without further purification.

HRMS *m/z* calc. for $C_{17}H_{36}O_3S$ $[M+Na]^+$ 343.2283, obs. $[M+Na]^+$ 343.2273 (2.91 ppm error).

Lipid ether amine (22)[10]

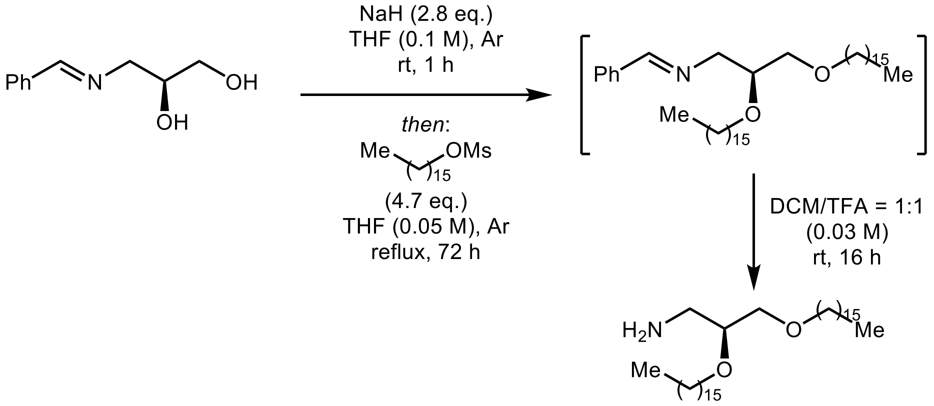

To an oven-dried flask was added NaH (180 mg, 4.50 mmol, 2.8 eq., 60% dispersion in mineral oil) under an atmosphere of argon. A solution of compound **19** (287 mg, 1.60 mmol) in anhydrous THF (16 mL) was added, and the mixture was stirred at rt for 1 h. To the reaction mixture was added a solution of the crude cetyl mesylate **21** (2.57 g, 7.50 mmol, 4.7 eq.) in anhydrous THF, dropwise. The reaction mixture was heated to reflux and stirred for 72 hrs. The reaction was quenched by the addition of water (50 mL) and the aqueous layer extracted with DCM (3 × 30 mL). The combined organic extracts were washed with water (30 mL), brine (30 mL), then dried ($MgSO_4$), filtered and concentrated *in vacuo*. The resultant oil was dissolved in wet DCM/TFA (1:1, 50 mL) and stirred at rt for 16 h to hydrolyse the imine. Reaction progress was monitored by TLC. Upon reaction completion, the reaction mixture was concentrated *in vacuo* and the TFA co-evaporated with toluene (3 × 50 mL). The resultant brown oil was purified by flash column chromatography (0–20% MeOH/DCM DCM), to afford the title compound a brown solid (**22**, 170 mg, 0.315 mmol, 29%).

HRMS *m/z* calc. for $C_{35}H_{73}NO_2$ $[M+H]^+$ 540.5719, obs. $[M+H]^+$ 540.5743 (4.44 ppm error).

^{1}H NMR (400 MHz, $CDCl_3$) δ 4.69 (br s, 2H, -N*H*), 3.62–3.55 (m, 1H, C*H*-O), 3.54–3.46 (m, 3H), 3.43 (t, *J* = 6.7 Hz, 2H, CH_2-O), 3.08 (dd, *J* = 13.0, 4.3 Hz, 1H, C$H_a$$H_b$-N), 2.97 (dd, *J* = 13.0, 6.6 Hz, 1H, C$H_a$$H_b$-N), 1.60–1.51 (m, 4H, C$H_2$-C$H_2$-O), 1.31–1.25 (m, 52H, -(CH_2)$_{13}$-), 0.88 (t, *J* = 6.8 Hz, 6H, -CH_3).

^{13}C NMR (101 MHz, $CDCl_3$) δ 75.9 (CH), 72.0 (CH_2), 70.9 (CH_2), 70.4 (CH_2), 42.3 (CH_2), 31.9 (CH_2), 30.0 (CH_2), 29.7 (CH_2), 29.7 (CH_2), 29.7 (CH_2), 29.7 (CH_2), 29.6 (CH_2), 29.6 (CH_2), 29.5 (CH_2), 29.4 (CH_2), 26.1 (CH_2), 22.7 (CH_2), 14.1 (CH_2).

MalLipid (5)[10]

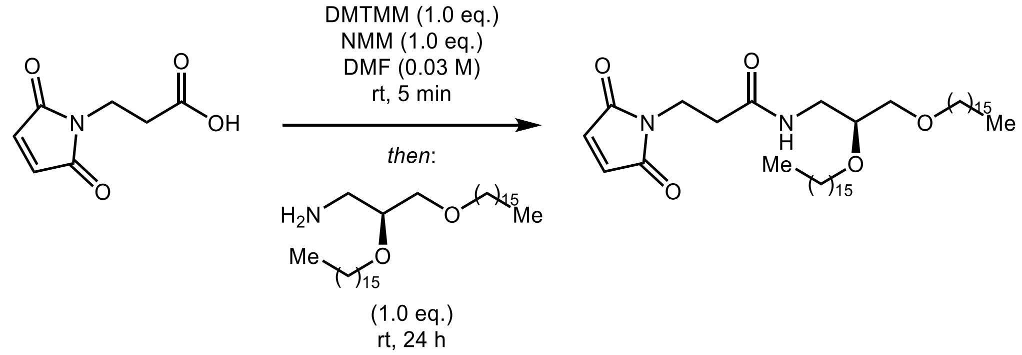

To a solution of 3-maleimidopropionic acid (**23**, 27 mg, 0.16 mmol) in DMF (5 mL) was added DMTMM (44 mg, 0.16 mmol, 1.0 eq.) and *N*-methylmorpholine (32 mg, 0.32 mmol, 2.0 eq.). The reaction mixture was stirred for 5 minutes before the addition of lipid ether amine (**22**, 85 mg, 0.16 mmol, 1.0 eq.). The solution was stirred at rt for 24 h, then acidified with 1M HCl. The reaction mixture was extracted with EtOAc (3 × 50 mL). The combined organic layers were washed with water (3 × 50 mL) and brine (50 mL), dried ($MgSO_4$), filtered, and concentrated *in vacuo*. The resultant oil was purified by flash column chromatography (SiO_2, 0–10% MeOH/DCM) to afford the title compound as a waxy solid (**5**, 23 mg, 33.3 µmol, 21%). The analytical data was consistent with those reported in the literature.[10]

HRMS *m/z* calc. for $C_{42}H_{78}N_2O_5$ $[M+Na]^+$ 713.5809, obs. $[M+Na]^+$ 713.5803 (0.85 ppm error).

^{1}H NMR (400 MHz, $CDCl_3$) δ 6.69 (s, 2H, C*H*=C*H*), 5.95 (t, *J* = 5.5 Hz, 1H, -N*H*), 3.84 (t, *J* = 7.3 Hz, 2H, CH_2-CH_2-N), 3.59–3.36 (m, 9H, C$H_a$$H_b$-N, C*H*-O, C$H_2$-O), 3.30–3.25 (m, 1H, C$H_a$$H_b$-N), 2.50 (t, *J* = 7.3 Hz, 2H, CH_2-CH_2-N), 1.55 (p, *J* = 6.8 Hz, 4H, CH_2-CH_2-O), 1.33–1.25 (m, 52H, -(CH_2)$_{13}$-), 0.88 (t, *J* = 6.7 Hz, 6H, -CH_3).

^{13}C NMR (101 MHz, $CDCl_3$) δ 170.6 (C), 169.6 (C), 134.3 (CH), 76.6 (CH), 72.0 (CH_2), 71.7 (CH_2), 70.3 (CH_2), 41.0 (CH), 34.8 (CH_2), 34.4 (CH_2), 32.1 (CH_2), 30.2 (CH_2), 29.9 (CH_2), 29.8 (CH_2), 29.8 (CH_2), 29.7 (CH_2), 29.6 (CH_2) 26.3 (CH_2), 22.8 (CH_2), 14.3 (CH_3).

NMR Spectra

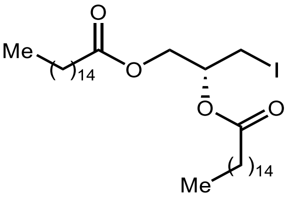

9

^{1}H NMR ($CDCl_3$, 400 MHz)

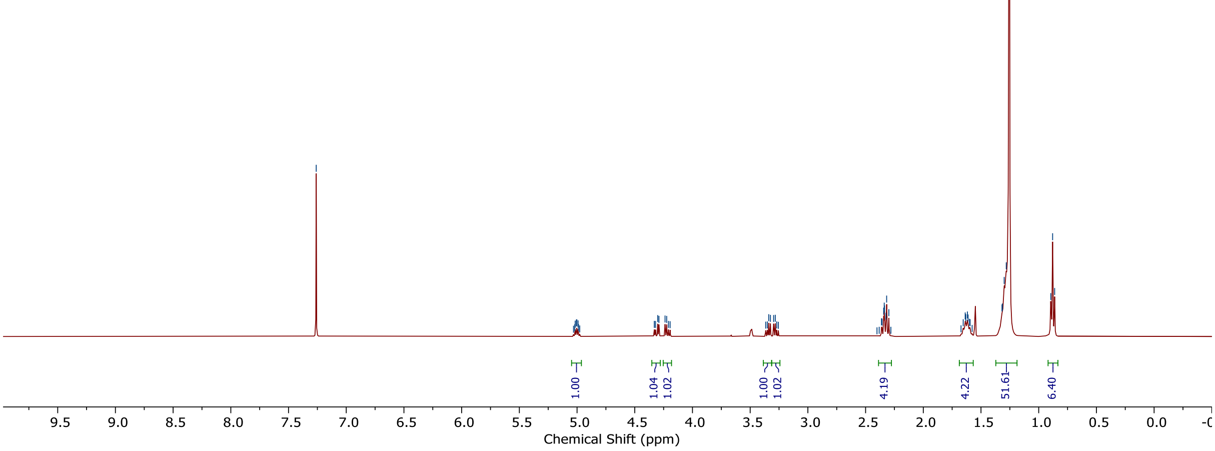

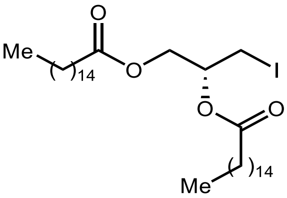

9

^{1}H NMR ($CDCl_3$, 400 MHz)

(5.5–0.5 ppm region)

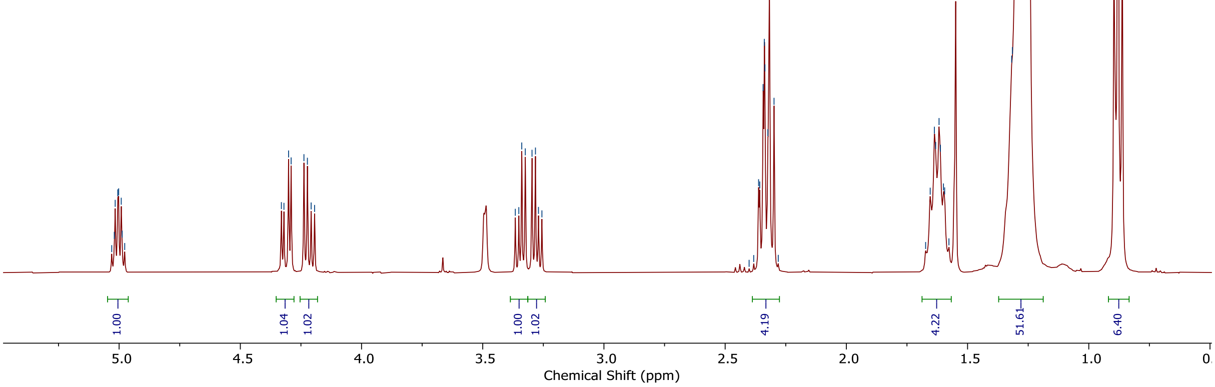

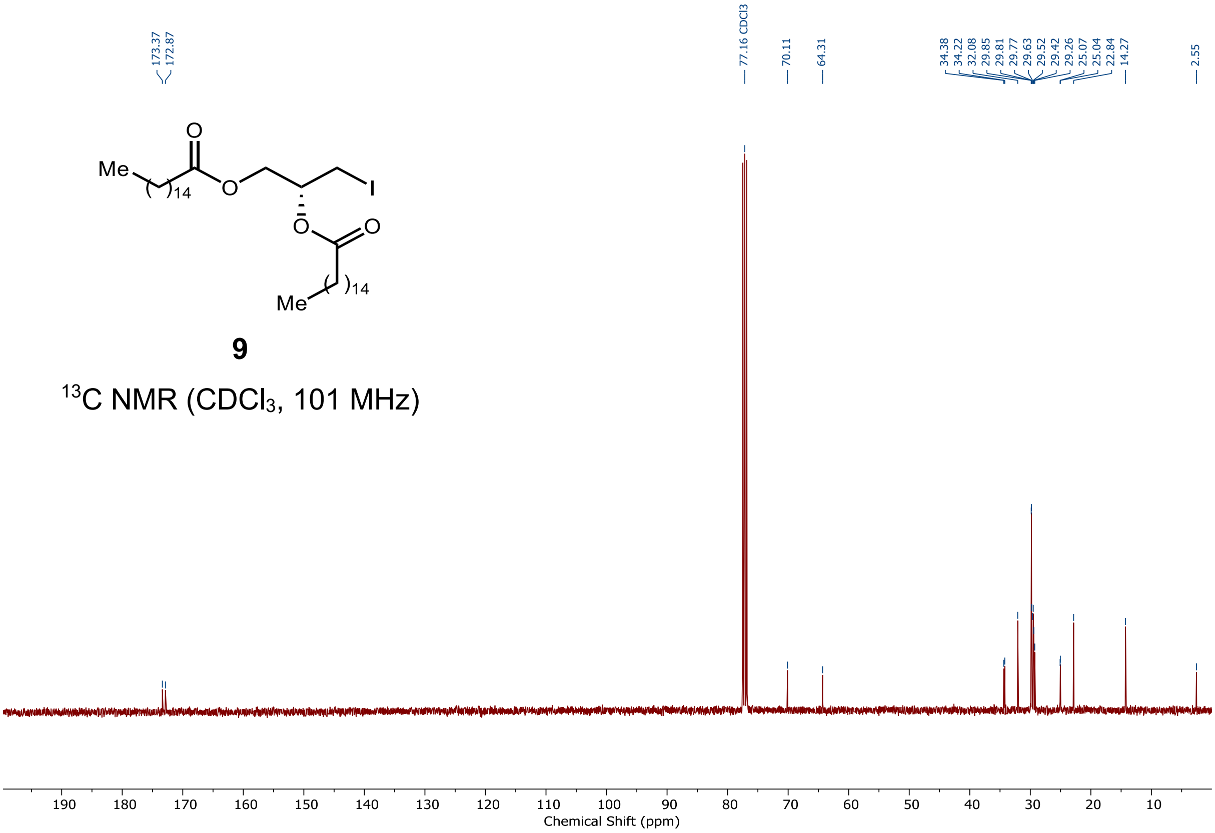

9

^{13}C NMR ($CDCl_3$, 101 MHz)

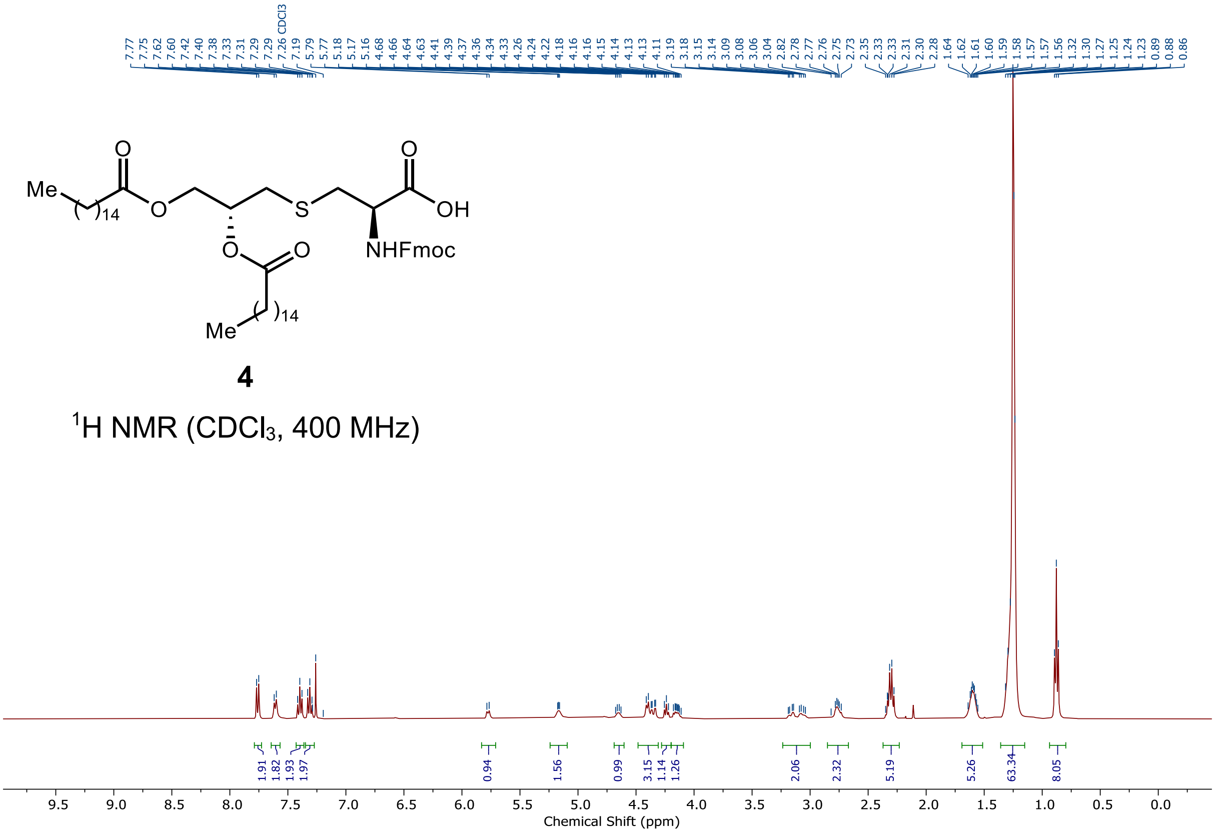

4

^{1}H NMR ($CDCl_3$, 400 MHz)

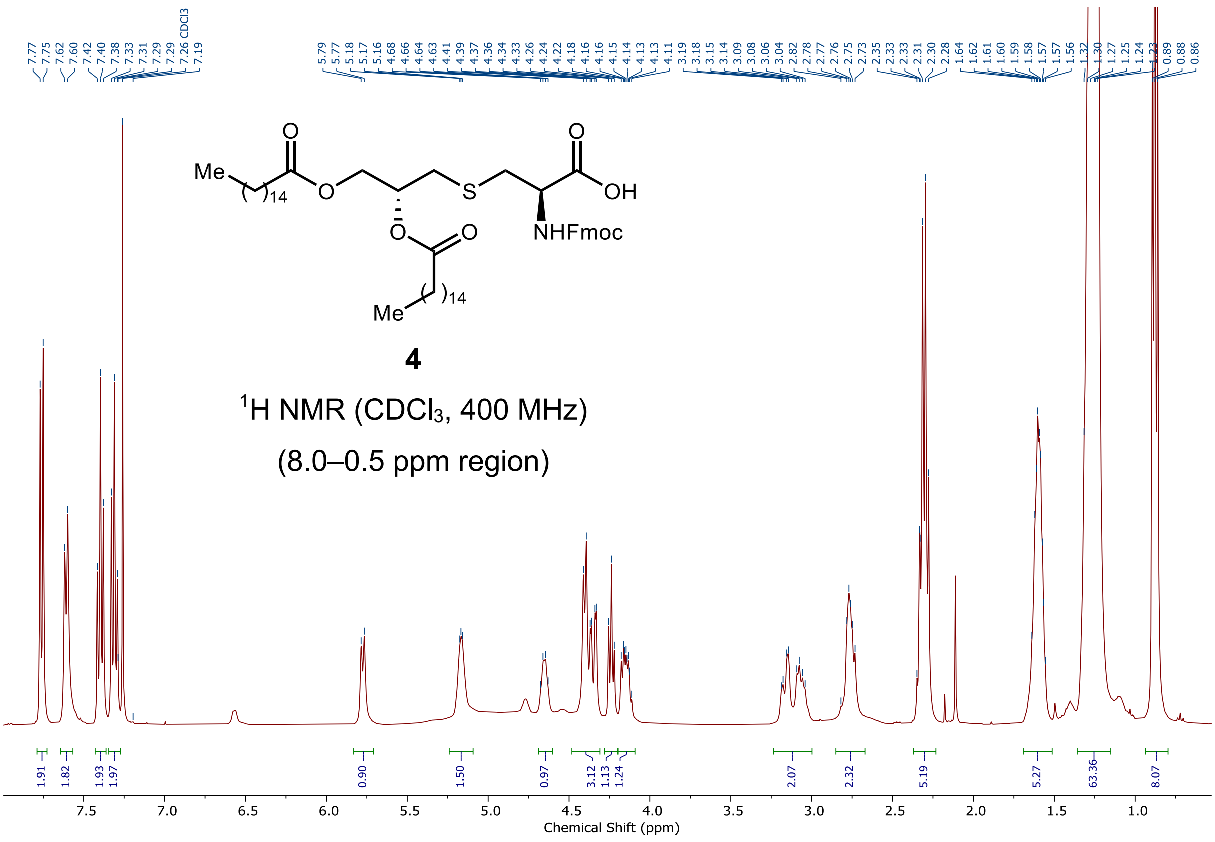

Me
O
O
S
O
OH
O
O
NHFmoc
14
14
Me
4
¹H NMR (CDCl₃, 400 MHz)
(8.0–0.5 ppm region)
Chemical Shift (ppm)

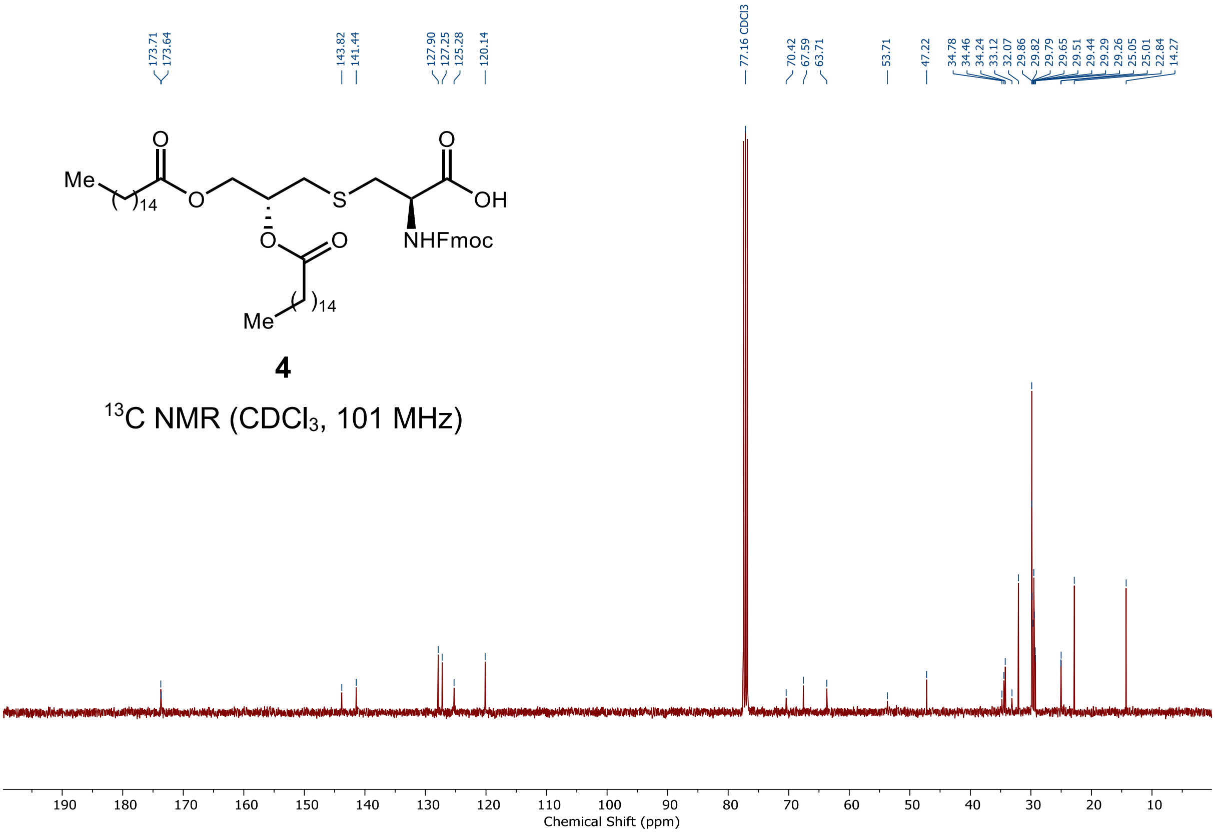

Me
O
O
S
O
OH
O
O
NHFmoc
14
14
Me
4
¹³C NMR (CDCl₃, 101 MHz)
Chemical Shift (ppm)

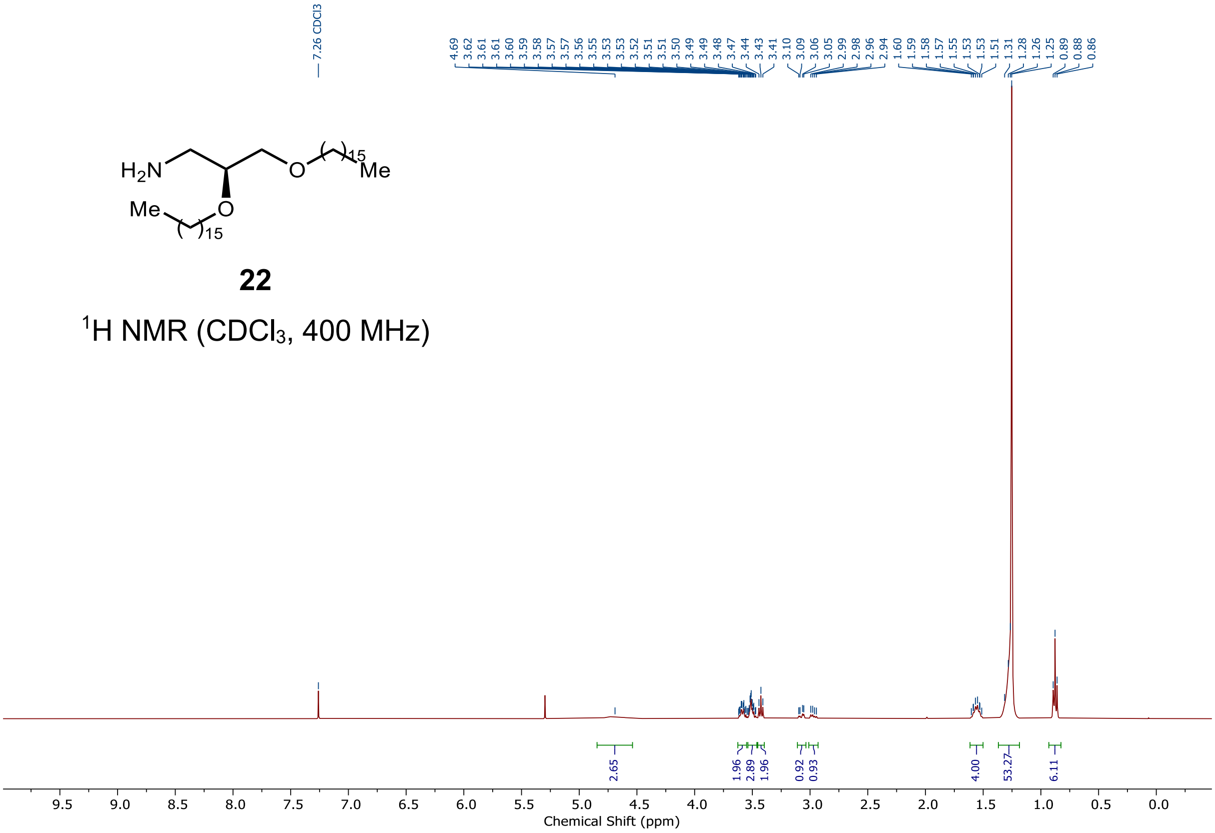

22

^{1}H NMR ($CDCl_3$, 400 MHz)

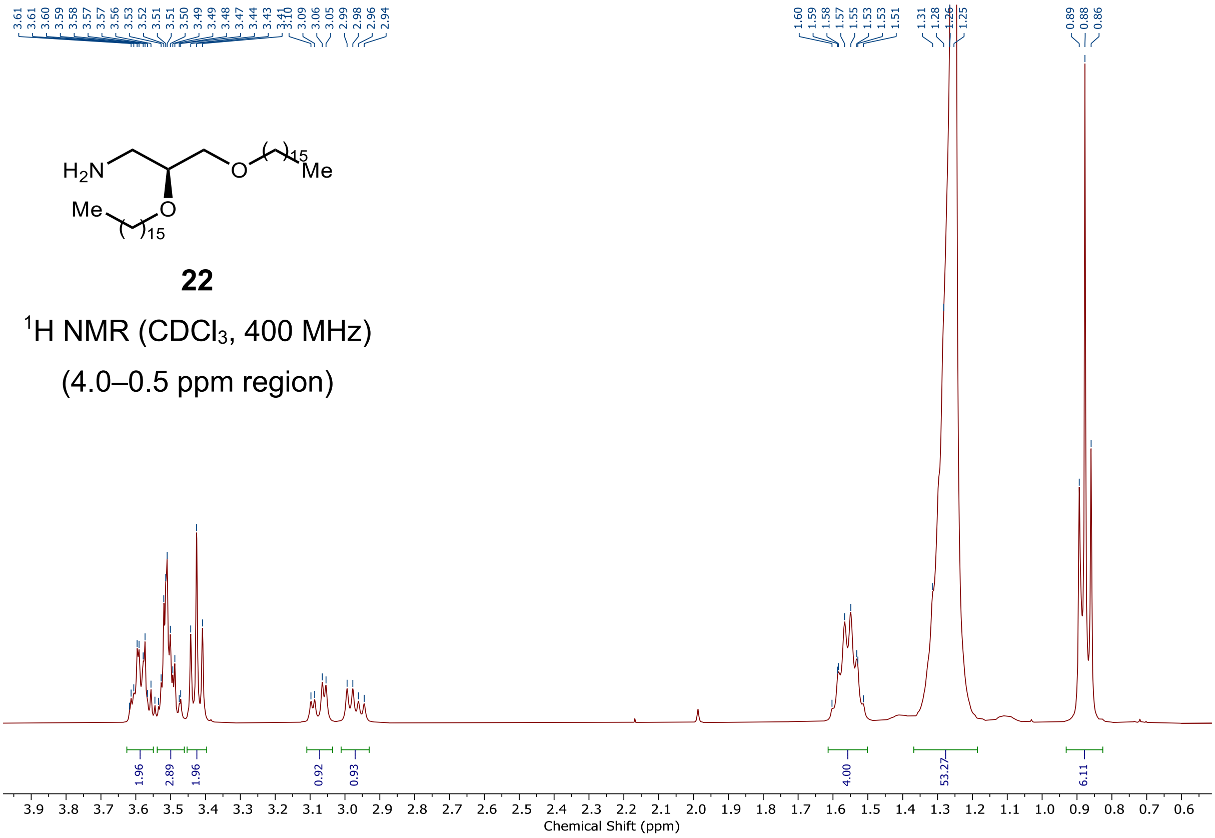

22

^{1}H NMR ($CDCl_3$, 400 MHz)

(4.0–0.5 ppm region)

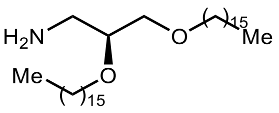

22

^{13}C NMR ($CDCl_3$, 101 MHz)

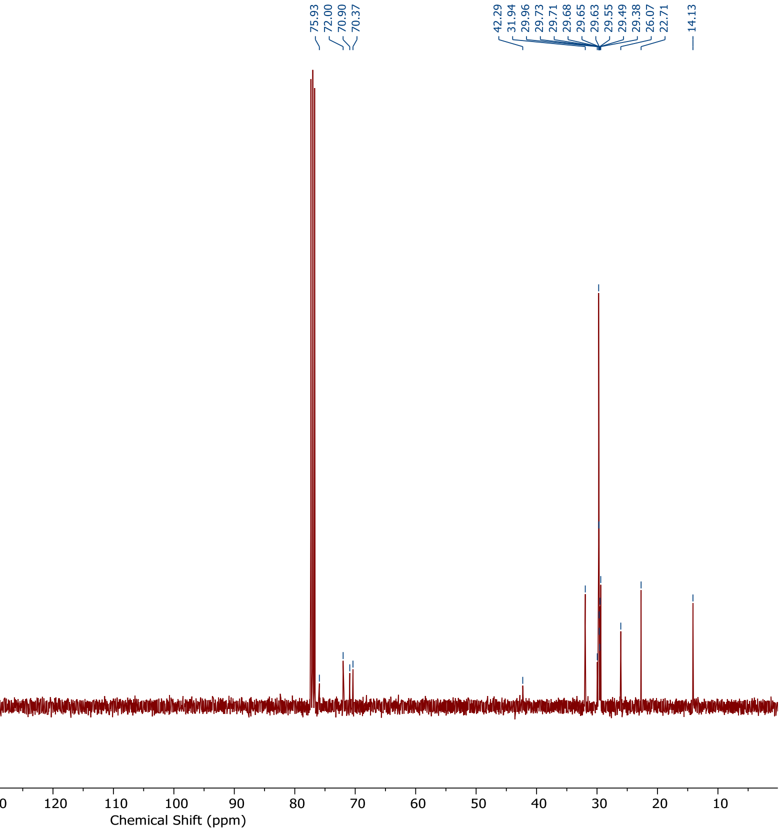

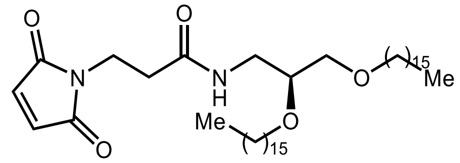

5

^{1}H NMR ($CDCl_3$, 400 MHz)

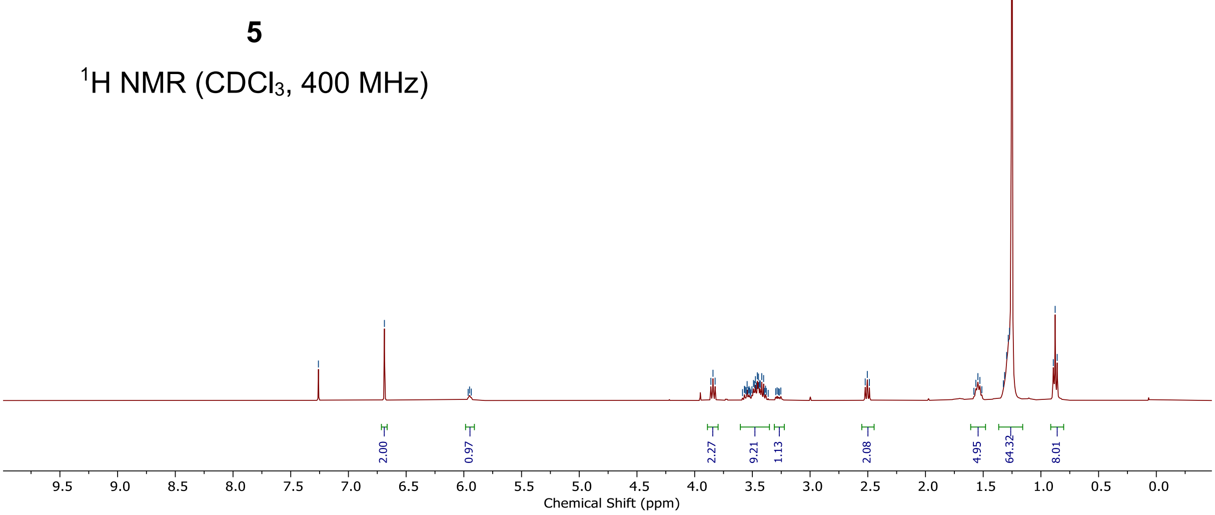

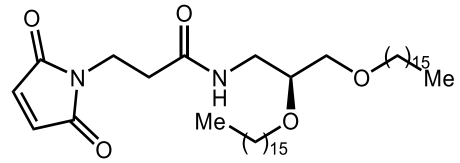

5

^{1}H NMR ($CDCl_3$, 400 MHz)

(4.5–0.5 ppm region)

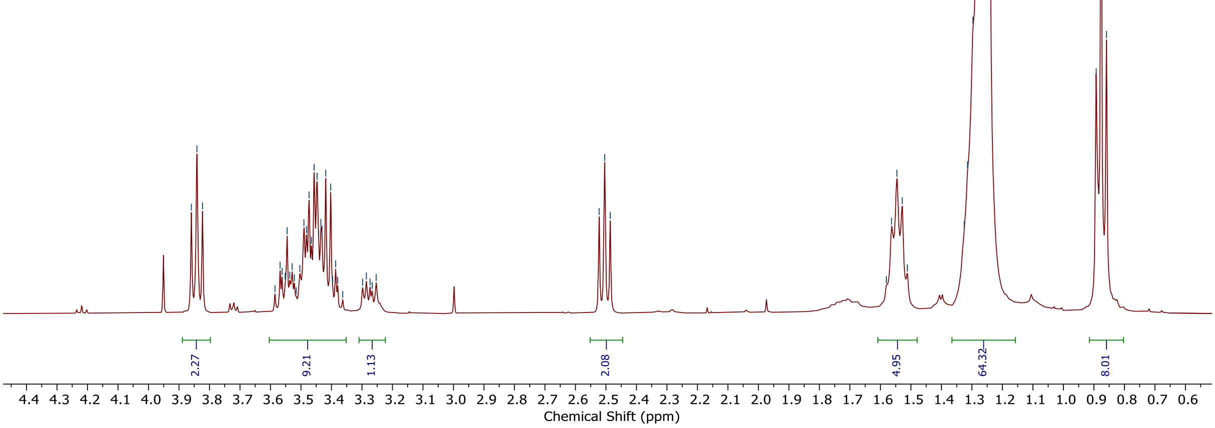

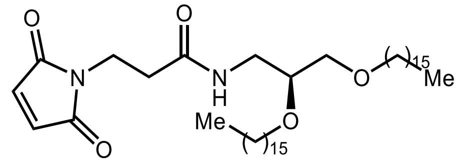

5

^{13}C NMR ($CDCl_3$, 101 MHz)

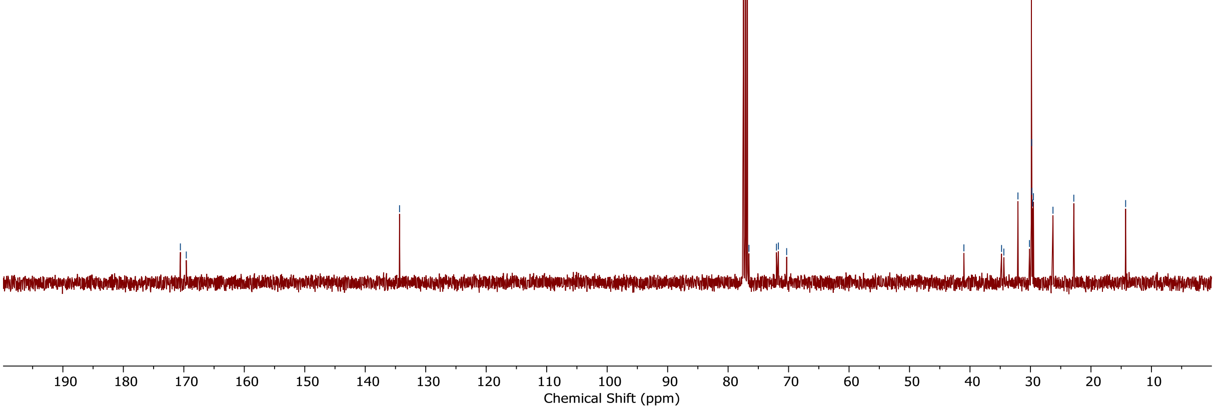

Synthesis of Lipid Nanoparticles

Anionic & cationic LNPs were formulated using the lipid film hydration method.[11]

$Pam_2Cys-SK_4-BAGE4_{18-39}$ Lipid Nanoparticle (2-LNP)

All lipids (DSPC, DOTAP and cholesterol) were dissolved in chloroform and $Pam_2Cys-SK_4-BAGE4_{18-39}$ **2** was dissolved in methanol prior to formulation. Solvents were evaporated slowly *in vacuo* to make a continuous lipid film, which was lyophilised overnight. Milli-Q water (1 mL) was added to hydrate the film with vortexing at 55°C. The resulting mixture was sonicated for 10 minutes, no pulsing, amplitude at 90% (SONICS Vibra-Cell™, CPX 130), and using ice bath resulting in formulated $Pam_2Cys-SK_4-BAGE4_{18-39}$ (**2**) vaccine construct.

Table S2. Summary of the reagents, mass, and volume used to formulate $Pam_2Cys-SK_4-BAGE4_{18-39}$ **2-**LNP (2 mM, 1 mL).

Reagents	mg	mmol	MW	Volume (µl)	%
DSPC	0.632	0.0008	790.1	400	40
DOTAP	0.209	0.0003	698.5	150	15
Cholesterol	0.271	0.0007	386.7	350	35
$Pam_2Cys-SK_4-BAGE4_{18-39}$	0.859	0.0002	3825	100	10

Maleimide LNPs (MalLNP)

All lipids (DSPC, MalLipid **5**, cholesterol, and DOTAP) were dissolved in chloroform. Chloroform was slowly evaporated *in vacuo* in a rotary evaporator to make a continuous lipid film, which was dried *in vacuo* for 4 h. PBS (5 mL or 2.5 mL) was added to hydrate the film with vortexing at 55 °C for 1–2 min. The resulting mixture was sonicated for 10 minutes, no pulsing, amplitude@ 90%, and using ice bath resulting in a 2 mM LNP solution.

Table S3: Summary of the reagents, mass, and volume used to formulate MalLNP (2 mM, 2.5 mL).

Reagents	mg	mmol	MW	Volume (mL)	%
DSPC	1.58	0.002	790.145	1.0	40
DOTAP	0.524	0.00075	698.54	0.375	15
Cholesterol	0.676	0.00175	386.65	0.875	35
Mal-Lipid **5**	0.345	0.0005	690	0.25	10

Cys-SK$_4$-BAGE4$_{18\text{-}39}$-LNP

1200 µL of 2 mM MalLNP formulation (containing 0.24 µmol lipid **5**) was conjugated to Cys-BAGE4$_{18\text{-}39}$ (0.48 µmol, 2.0 eq.) of in the presence of TCEP, at pH 7. The reaction mixture was vortexed at rt for 3 h. The sample was loaded into Slide-A-Lyzer Dialysis Cassette (10k MWCO) then floated in dialysate buffer (PBS), 500 times the volume of loaded sample. The dialysate buffer was changed after 2 h, 3 times, the last for an overnight period. The sample was then recovered and analysed by UV-vis spectroscopy at 280 nm.

Determination of Electrostatic Loading of LNPs

Table S4: Quantification of electrostatic peptide loading.

LNP	A_{280}	Volume / mL	Bound Peptide / µmol[a]
MalLNP	0.826	0.8	0.06
NonMal-LNP	0.245	0.8	0.02

[a] Using estimated extinction coefficient for H–Cys-BAGE4$_{18\text{-}39}$–NH_2 = 11380 cm^{-1} M^{-1}

LNP Characterisation

MalLNP and **2**-LNP were analysed using DLS to measure the particle size and PDI. All samples were diluted 10 times with Milli-Q water and transferred to disposable cuvettes before measurement at 25 °C with a 173° light scattering angle.

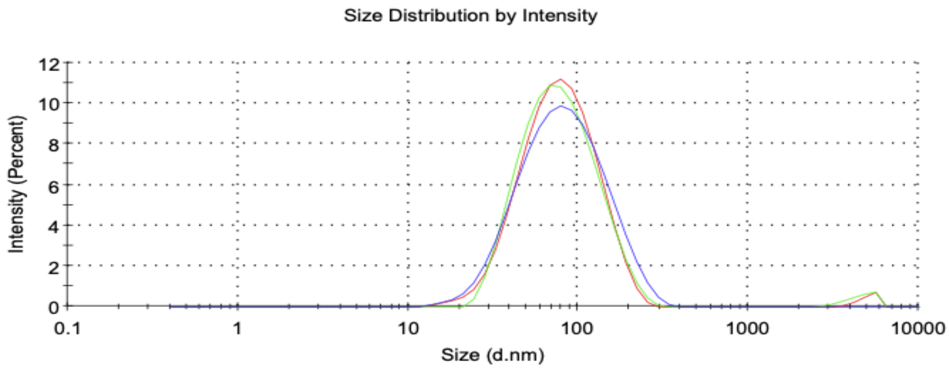

Figure S20. DLS spectra of MalLNP size distributions by intensity. Z-Average (d.nm): 70.28, PdI: 0.236; Zeta potential of +30 mV.

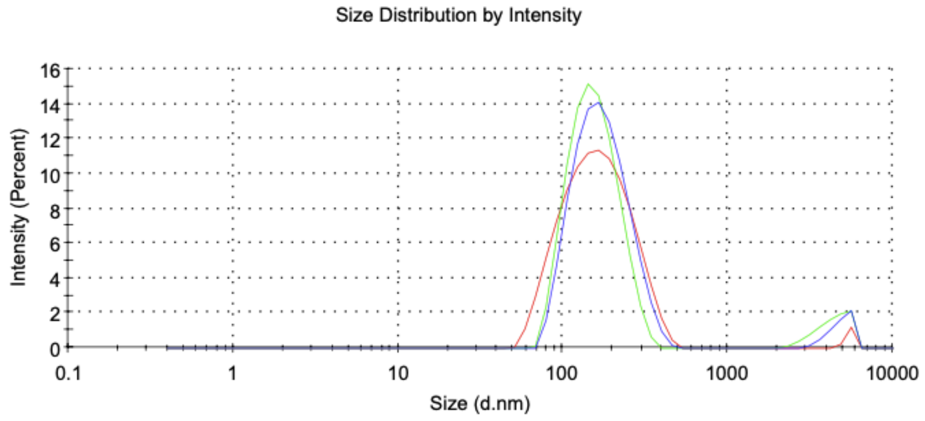

Figure S21. DLS spectra of Pam_2Cys-SK_4-$BAGE4_{18\text{-}39}$–NH_2 LNP (**2**-LNP) size distributions by intensity. Z-Average (d.nm): 164.5, PdI : 0.304; Zeta potential of +10 mV.

MalLNP, and Cys-BAGE4-MalLNP formulations were characterized with Transmission Electron Microscopy (TEM) to determine the particle size and shape. All samples were diluted in Milli-Q water and applied to glow-discharged carbon-coated copper 200 mesh grids and negative-stained with 2% uranyl acetate.

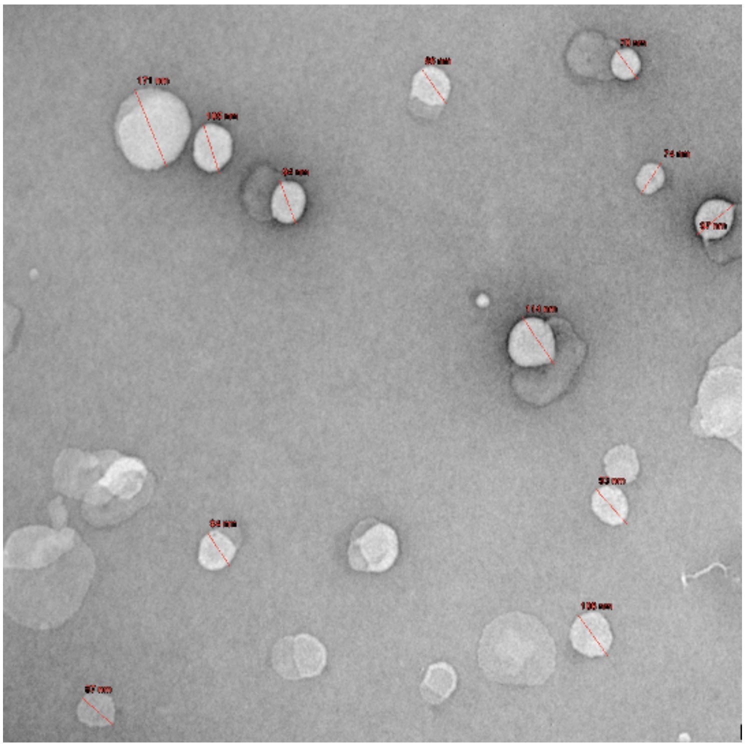

Figure S22. TEM for MalLNP, the image capturing was operated at 43k.

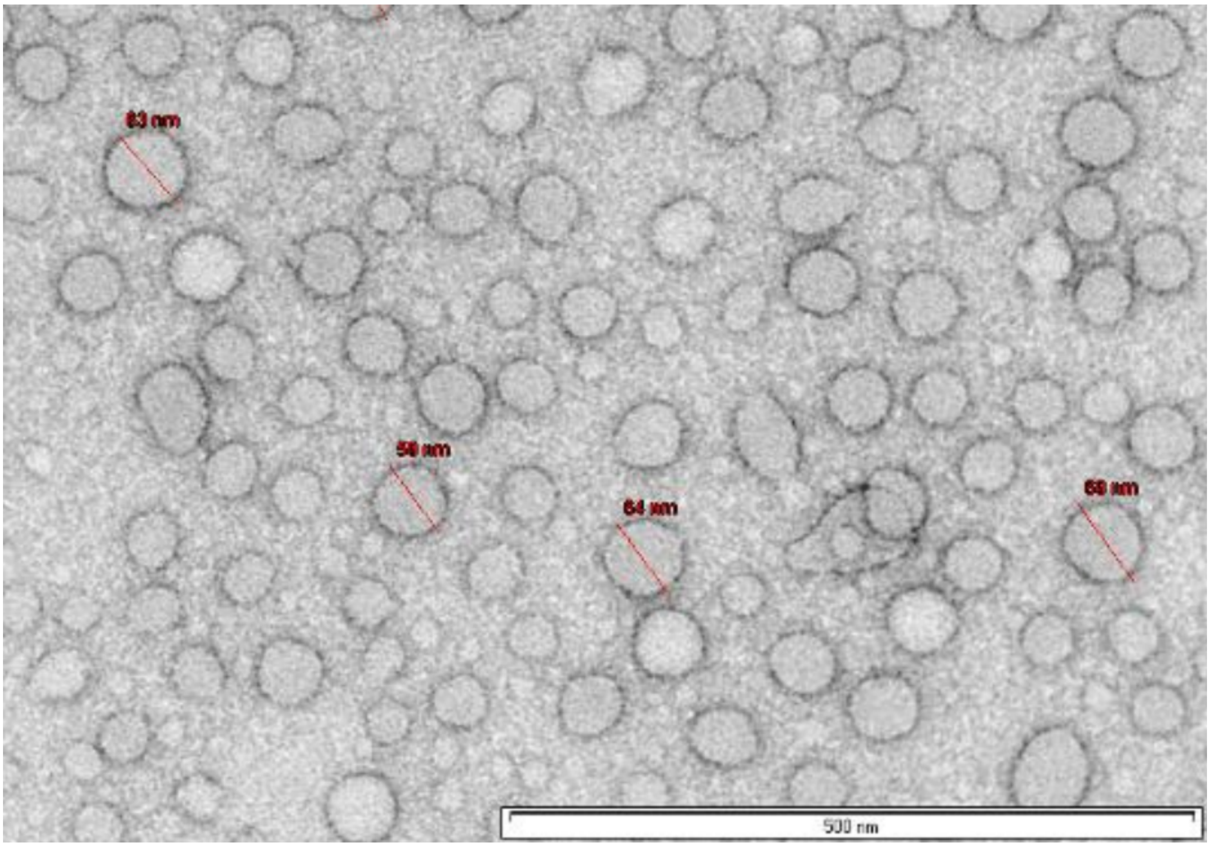

Figure S23. TEM for Cys-BAGE4-MalLNP the image capturing was operated at 43k.

Evaluation of Biological Activity

ELISA assay

Coating antigen

The concentration of stock samples used for ELISA for $BAGE4_{18-39}$ and Pam_2Cys-SK_4-$BAGE4_{18-39}$ **2** was 1 mM. The antigen was diluted to 100 ng/ml in carbonate-bicarbonate buffer, then 60 µL of the antigen dilution was pipetted into the wells of Maxisorp 384 well plate. The plate was diluted as required (23 dilutions were prepared) and incubated at 4 °C under foil overnight. The coating solution was removed and the plate washed three times by filling the wells with 200 µL PBST. The solutions or washes were removed by flicking the plate over a sink, then patting the plate dry with a paper towel.

Blocking

Remaining protein-binding sites in the coated wells were blocked by adding 40 µL blocking buffer (3% whey, 0.05% NaN_3, 0.1% EDTA in PBS) to each well. The plates were covered with foil and incubated for 2 h at rt or overnight at 4 °C. Plates were washed three times by filling the wells with 200 µL PBS.

Incubation with primary and secondary antibody

In an Abgene 96 well plate, 8 dilutions of primary antibody (anti-BAGE4 produced in rabbit; Sigma Aldrich SAB4301150, and anti-NY-ESO-1 produced in mouse; Sigma Aldrich N2038) in PBST buffer and, 20 µL of each dilution of primary antibody was added to the coated 384 well plate in duplicate. The plate was covered and incubated for 1 h at rt, then washed four times with PBST. 20 µL of secondary HRP-anti rabbit IgG antibody (for BAGE4) and secondary HRP-anti mouse IgG antibody (for NY-ESO-1) (diluted at the optimal concentration according to the manufacturer in PBST buffer immediately before use) was added, then the plate was covered and incubated at rt for 1 h. Following incubation, the plate was washed four times with PBS.

Detection

40 µL of 3,3',5,5'-tetramethylbenzidine (TMB) solution was added to each well. The plate was incubated for 20–30 min, then an equal volume of stopping solution (2 M H_2SO_4) was added. The optical density at 450 nm was recorded.

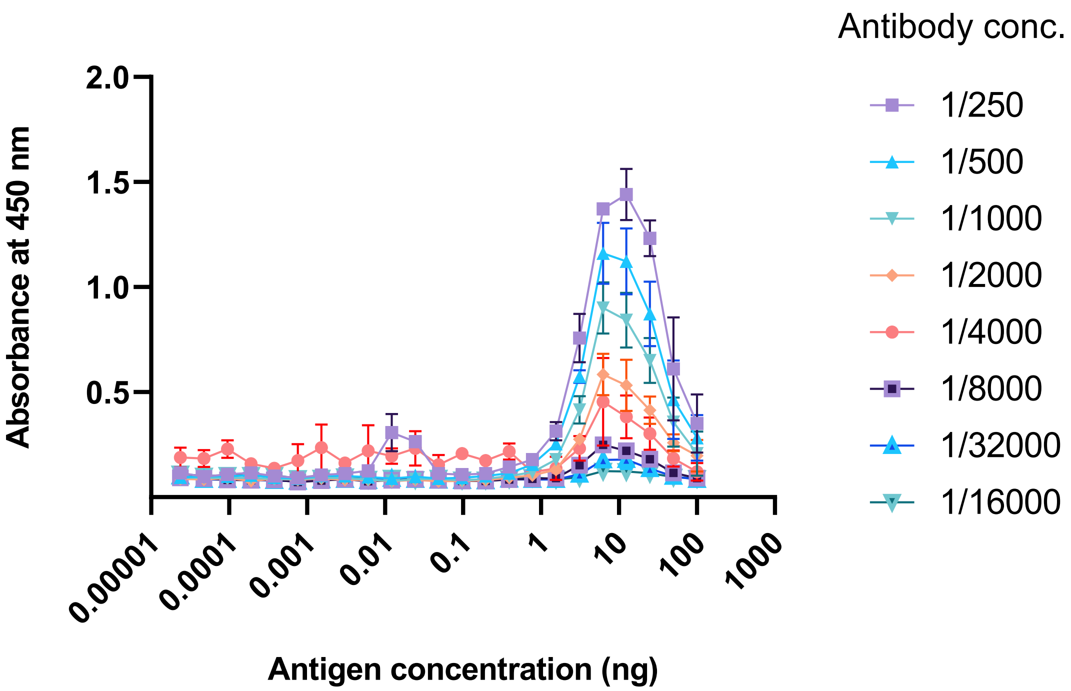

Figure S24. ELISA binding curves for BAGE4$_{18\text{-}39}$ with a serial dilution series of Anti-BAGE4 antibody, absorbance measured at 450 nm.

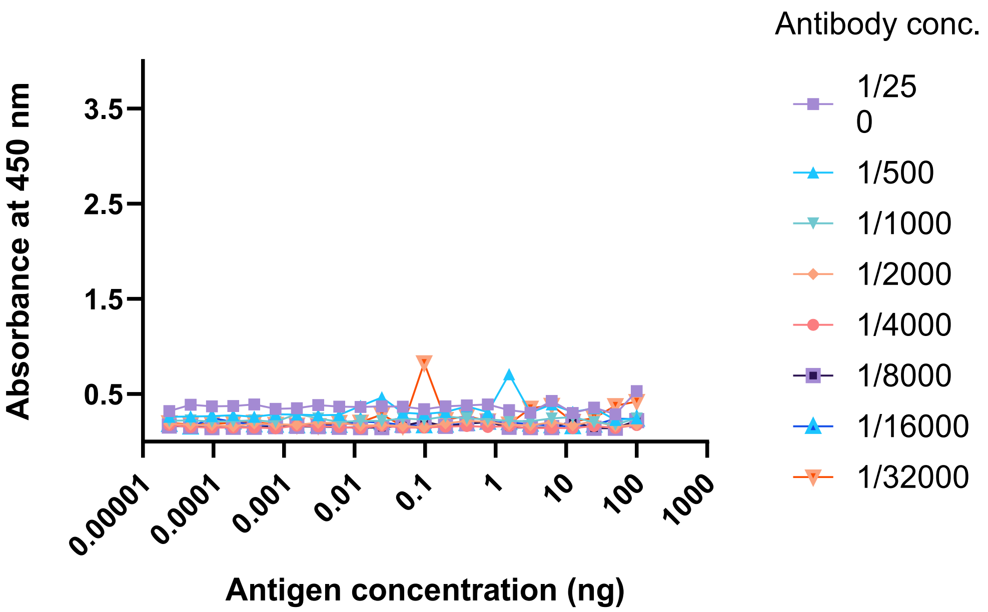

Figure S25. ELISA binding curves for NY-ESO-1$_{157\text{-}165}$ with a serial dilution series of Anti-NY-ESO-1 antibody, absorbance measured at 450 nm.

Competitive ELISA

For competitive ELISA, the coating antigen, blocking, and detection steps are as described above.

Incubation with primary and secondary antibody

In an Abgene 96 well plate, 60 µl of BAGE4 solution (1000 ng/mL, 1 µM) was added to the first two wells of the first column of the plate and diluted twofold across each column of the plate. The same process was followed using lipopeptide (Pam$_2$Cys-SK$_4$-BAGE4$_{18\text{-}39}$ **2**) in the next two rows of the plate, and for Cys-BAGE4-Mal LNP in the next two rows of the plate. No antigen was added to the last two rows of the plate. 20 µL of primary anti-BAGE4 (produced in rabbit; Sigma Aldrich SAB4301150) antibody was added to each well at the optimized concentration (1:250) and incubated for 1 h. The incubated BAGE4$_{18\text{-}39}$, Pam$_2$Cys-SK$_4$-BAGE4$_{18\text{-}39}$ (**2**), and Cys-BAGE4-MalLNP with primary antibody were transferred to a washed coated plate with antigen (BAGE4) and incubated for 1 h, then the plate was washed four times with PBST. 20 µL of secondary antibody–HRP (Polyclonal Swine Anti-Rabbit Immungic–HRP) (diluted 1:4000 according to the manufacturer in PBST buffer immediately before use) was added. The plate was incubated for 1 h, then washed four times with PBS.

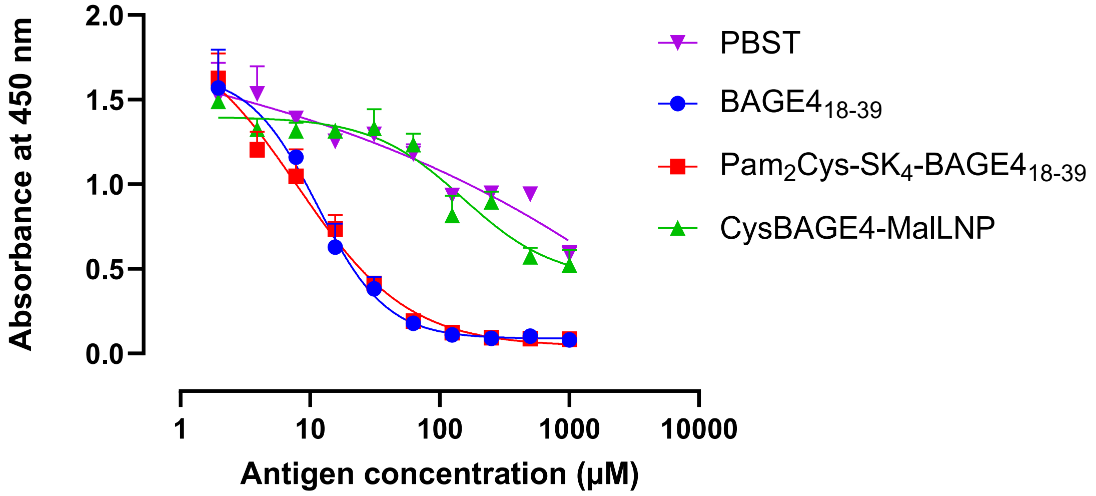

Figure S26. ELISA data to compare the binding affinity of BAGE4$_{18\text{-}39}$ antigen IC$_{50}$= 11.41 mM +/- 3.52, Pam$_2$Cys-SK$_4$-BAGE4$_{18\text{-}39}$ (**2**) IC$_{50}$ = 8.04 mM +/- 6.04 Cys-BAGE4-MalLNP IC$_{50}$ = 151.4 mM, PBST buffer with 0.05% Tween 20 as negative control.

In Vivo Evaluation (ELISpot Assay)

Animal experiments were carried out with ethical approval from University of Nottingham ethical review boards and under a Home Office approved project license (PP2706800). HLA-HHDII/DR1 (HLA-A2.1+/+ HLADR1+/+, Pasteur Institute) transgenic mice knocked out for murine MHC-I and II and expressing chimeric HLA-A2 (HHDII) and human HLA-DR1 or Balb/c mice (Charles River) aged 6-12 weeks were used. Mice were vaccinated with 10 ηmol of each immunogen at each timepoint. Doses were delivered subcutaneously (s.c.) in 50 μl volume on days 1, 8 and 15. No anaesthesia was used for injections. Responses were screened on day 21 after euthanasia by cervical dislocation. Preparation of media and buffers and steps performed on days 1 and 2 of the ELISpot assay were performed using a laminar flow cabinet and aseptic techniques to ensure the sterility of media, reagents and plates at stages before development of the ELISpot. Development of the ELISpot on day 4 can be performed on the laboratory bench.

The following tables summarise the details of the vaccine candidates and vaccination procedure including (dose time points):

Table S5. Summary of the injection schedule (including dose concentration and number) for Pam_2Cys-SK_4-NY-ESO-$1_{157-165}$ (**2**) candidate using HHDII/DR1 Tg mice.

Group	Test article	Dose (μg)/vol	Route	Dosing schedule	N	Terminal	Assays at termination
1	Pam_2Cys-SK_4-NY-ESO-1-	10 nmol in 50 μl	SC	Day 1,8 & 15	3	Day 21	ELISpot on splenocytes

Table S6. Summary of the injection schedule (including dose concentration and number) for the following vaccine candidates: $BAGE4_{18-39}$ candidate (co-administered with IFA), Cys-BAGE4-MalLNP conjugate, Pam_2Cys-SK_4-$BAGE_{18-39}$ (**2**), and **2**-LNP using BALB/c mice.

Group	Test article	Dose (μg)/vol	Route	Dosing schedule	N	Terminal	Assays at termination
1	$BAGE4_{18-39}$ peptide mixed with standard adjuvant (Incomplete Freunds)	10 nmol in 50 μl	SC	Day 1,8 & 15	3	Day 21	ELISpot on splenocytes ELISA on sera samples

2	Cys-BAGE4$_{18\text{-}39}$-Mal-LNP	10 nmol in 50 µl	SC	Day 1,8 & 15	3	Day 21	
3	Pam2Cys-SK$_4$-BAGE4$_{18\text{-}39}$	10 nmol in 50 µl	SC	Day 1, 8 & 15	3	Day 1	Experiment terminated
4	Pam$_2$Cys-SK$_4$-BAGE4$_{18\text{-}39}$ LNP formulation	10 nmol in 50 µl	SC	Day 1, 8 & 15	3	Day 1	Experiment terminated

ELISpot Assay

Elispot assays were performed using murine IFNγ capture and detection reagents according to the manufacturer's instructions (Mabtech).

Day 1: Coating of plates with capture antibody

20 µL 35% ethanol was added to each well then discarded. Each plate was then washed 4 times with 200 µL of sterile distilled water per well. The capture antibody (mAb AN 18, 1 mg/mL) was diluted to 10 µg/mL (1:100) in sterile PBS, and 50 µL of the diluted capture antibody was added per well to MAIP multiscreen ELISpot plate(s), ensuring that the bottom of the wells were completely covered with coating antibody solution. Each plate was covered and incubated at 4°C overnight.

Day 2: Blocking plates

The capture antibody solution was discarded, and each plate was washed 4 times with 200 µL of sterile PBS per well. PBS was discarded, and 100 µL Complete Medium (RPMI medium 1640 (GIBCO/BRL) supplemented with 10% FCS (Sigma), 2mM L-glutamine (Sigma) and sodium bicarbonate buffered with additional 20mM HEPES (Sigma)) was added to each well. The plates were incubated at rt for between 1 and 24 h.

Preparation of effector cells from spleens

Mice were laid on their right side, on a paper towel, in the hood, and their skin wetted with 70% ethanol to minimise the risk of bacterial contamination. Using large scissors, the skin above the spleen region was cut away. Small scissors were used to cut open the cavity exposing the spleen, which was removed and transferred to a petri dish containing 10 mL

complete medium, and warmed to 37 °C. 10 mL of complete medium was drawn into a 20 mL syringe with a 21-gauge needle. The outer capsule of the spleen was punctured with a needle, and splenocytes flushed out of the spleen with gentle squeezing. The cells were transferred to a sterile 25 mL conical universal tube. Clumps were allowed to settle for 5 minutes, then the cell suspension was decanted into a fresh 25 mL conical universal tube. Centrifugation at 1000 rpm for 10 minutes in a benchtop centrifuge yielded a pellet, which was resuspended in 10 mL complete medium containing 2-mercaptoethanol. Viable cells were counted by mixing equal volumes (50 µL) of cell suspension and 0.4% Trypan blue and transferring 9 µL to a FastRead disposable haemocytometer. Note: Trypan blue is taken up by nonviable but not by viable cells. Nonviable cells appear blue while viable cells remain colourless. Cells were resuspended to a concentration of 5×10^6 cells/mL in complete medium containing 50µM 2-mercaptoethanol (Thermofisher).

Addition of splenocytes and peptide to ELISpot plates

Media was discarded from blocked ELISpot plate(s), and cells from splenocyte harvest were added at 100 µL/well (5×10^5 cells) in complete medium plus 2-mercaptoethanol (5×10^{-6} M). Assay performed in quadruplicate. Peptides were diluted appropriately in complete media plus 2-mercaptoethanol (10 µg/mL to 0.01 µg/mL range) and added to the relevant wells in ELISpot plate. Media control (no peptide) was included as a negative control and LPS (5 µg/mL) as a positive control. The plate was incubated at 37°C for approx. 40 h.

Day 4: Development of ELISpot

Note: At this step conditions are non-sterile and all further procedures can be carried out on the bench.

Cells were discarded from the ELISpot plate, and the plate was washed 5 times with 200 µl/well 211 PBS 0.05% Tween. 50 µL biotinylated detection antibody (murine IFNγ ELISpot kit) diluted 1:1000 in PBS was added to each well. The plates were incubated at room temp for 2.5 h, then the antibody was discarded, and the plates washed 5 times with 200 µL/well PBS 0.05%Tween. 50 µL streptavidin Alkaline Phosphatase (murine IFNγ ELISpot kit) diluted 1:1000 in PBS was added to each well. The plates were incubated for 1.5 h at rt. The plates were flicked off and washed 6 times with 200 µL/well PBS 0.05% Tween. 50 µL development solution was added to each well, and the plate was left in the dark at rt for 5 min to develop. Development solution (for 1 plate, AP conjugate substrate kit, BioRad) was added in the order:

4.8 mL H_2O, 200 µL Development buffer (well mixed), 50 µL reagent A (from Alkaline Phosphatase substrate kit), 50 µL reagent B (from Alkaline Phosphatase substrate kit). Once spots were observed on the plate, it was washed under the tap to stop the reaction, and the plastic plate back was removed to wash the back of the wells. The plate was left to dry at rt, and spots were counted on the ELISpot reader.

References

[1] U. Baxa, in *Characterization of nanoparticles intended for drug delivery*, Springer, **2017**, pp. 73-88.

[2] N. P. Cowieson, C. J. C. Edwards-Gayle, K. Inoue, N. S. Khunti, J. Doutch, E. Williams, S. Daniels, G. Preece, N. A. Krumpa, J. P. Sutter, M. D. Tully, N. J. Terrill, R. P. Rambo, *J. Synchrotron Radiat.* **2020**, *27*, 1438-1446.

[3] I. W. Hamley, *Small-Angle Scattering*, Wiley, Chichester, **2021**.

[4] aI. Bressler, J. Kohlbrecher, A. F. Thunemann, *J. Appl. Crystallogr.* **2015**, *48*, 1587-1598; bJ. Kohlbrecher, I. Bressler, *J. Appl. Crystallogr.* **2022**, *55*, 1677-1688.

[5] N. Martin, E. J. Thomas, *Org. Biomol. Chem.* **2012**, *10*, 7952-7964.

[6] M. Kurimura, M. Takemoto, K. Achiwa, *Chem. Pharm. Bull.* **1991**, *39*, 2590-2596.

[7] B. L. Lu, F. F. Li, I. D. Kelch, G. M. Williams, P. Rod Dunbar, M. A. Brimble, *Eur. J. Org. Chem.* **2021**, *2021*, 5415-5423.

[8] B. L. Lu, G. M. Williams, D. J. Verdon, P. R. Dunbar, M. A. Brimble, *J. Med. Chem.* **2020**, *63*, 2282-2291.

[9] G. P. Gential, N. I. Ho, F. Chiodo, N. Meeuwenoord, F. Ossendorp, H. S. Overkleeft, G. A. van der Marel, D. V. Filippov, *Bioorg. Med. Chem. Lett.* **2016**, *26*, 3641-3645.

[10] C. Karyal, P. Palazi, J. Hughes, R. C. Griffiths, R. R. Persaud, P. J. Tighe, N. J. Mitchell, R. Griffin, *Vaccines* **2021**, *9*, 1453.

[11] N. Mitchell, T. L. Kalber, M. S. Cooper, K. Sunassee, S. L. Chalker, K. P. Shaw, K. L. Ordidge, A. Badar, S. M. Janes, P. J. Blower, M. F. Lythgoe, H. C. Hailes, A. B. Tabor, *Biomaterials* **2013**, *34*, 1179-1192.
